# Characterizing hepatitis C virus epidemiology in Egypt: systematic reviews, meta-analyses, and meta-regressions

**DOI:** 10.1038/s41598-017-17936-4

**Published:** 2018-01-26

**Authors:** Silva P. Kouyoumjian, Hiam Chemaitelly, Laith J. Abu-Raddad

**Affiliations:** 1 Infectious Disease Epidemiology Group, Weill Cornell Medicine-Qatar, Cornell University, Qatar Foundation - Education City, Doha, Qatar; 2 Department of Healthcare Policy & Research, Weill Cornell Medicine, Cornell University, New York, USA

## Abstract

Egypt is the most affected nation by hepatitis C virus (HCV) and needs a comprehensive characterization of HCV epidemiology to inform the scale-up of treatment and prevention programs. Systematic reviews, meta-analyses, and meta-regressions were conducted. A total of 25 incidence, 259 prevalence, and 47 genotype studies were identified. Incidence and prevalence levels were high across all populations. Genotype 4 accounted for 94.1% of infections with a relative Shannon Diversity Index of only 14.4%. Pooled mean HCV prevalence was estimated at 11.9% (95% CI = 11.1–12.6%) among the general population, 55.6% (95% CI = 49.4–61.7%) among populations at high risk, 14.3% (95% CI = 10.3–18.8%) among populations at intermediate risk, 56.0% (95% CI = 50.4–61.6%) among populations with liver-related conditions, and 35.0% (95% CI = 27.3–43.1%) among special clinical populations. Mean HCV viremic rate was estimated at 66.7% (95% CI = 61.7–71.5%). Meta-regression indicated 6% lower odds for HCV prevalence for each one-year increment in publication year (AOR = 0.94; 95% CI = 0.92–0.96). HCV prevalence is high with evidence for ongoing transmission mainly through healthcare. Genotype diversity is low with genotype 4 dominance. Two-thirds of antibody-positive Egyptians are chronically infected and need treatment. Clinical populations should be prioritized for screening. Despite the large-scale epidemic, prevalence appears to be declining rapidly consistent with a contracting epidemic.

## Introduction

Viral hepatitis was estimated to be the 7^th^ leading cause of mortality globally^1^. About half of this mortality is attributed to hepatitis C virus (HCV), a primary cause for liver fibrosis, cirrhosis and cancer^2,3^. The recent development of highly efficacious oral direct-acting antivirals (DAAs) provides opportunities for reducing HCV disease burden and its onward transmission, with the potential for eliminating this blood-borne virus as a public health concern^4–7^. The World Health Organization (WHO) has recently formulated the ‘Global Health Sector Strategy on Viral Hepatitis, 2016–2021’^8^ with service coverage targets to eliminate HCV as a public health threat by 2030^8,9^. Action to combat viral hepatitis has now been integrated into the United Nations’ 2030 Agenda for Sustainable Development^10^.

One of the countries most affected by HCV is Egypt. The Egypt Demographic and Health Surveys (EDHS) measured antibody prevalence among the adult population aged 15–59 years at 14.7%^11^ in 2009 and at 10.0%^12^ in 2015—substantially higher than global levels^2,3,13^. To attend to this challenge, Egypt developed a national strategy for HCV control and established HCV prevention and treatment programs^14–16^. Following successful negotiations for 99% discounted DAA prices^17^, Egypt launched an ambitious national HCV treatment program aiming to treat over 250,000 chronically infected individuals per year, with the goal of achieving a national chronic infection prevalence of <2% by 2025^5,18^. Despite this progress, existing evidence suggests ongoing HCV transmission in Egypt, with higher incidence levels relative to other countries^5,19–21^.

The overarching goal of the present study was to provide a rigorous understanding of HCV epidemiology in Egypt with the ultimate aim of informing the rapidly expanding treatment and prevention national response. We aimed specifically to 1) characterize HCV infection levels across populations and subpopulations at various risks of exposure, 2) assess the diversity in HCV genotype and subtype distributions, 3) estimate the mean anti-HCV prevalence among populations and subpopulations, 4) estimate the mean HCV viremic rate, 5) identify individual-level risk factors for incident HCV infection and factors associated with being infected with HCV, and 6) identify population-level associations with anti-HCV prevalence in the general population adjusting for different sources of between-study heterogeneity.

This study is conducted under the umbrella of the ongoing Middle East and North Africa (MENA) HCV Epidemiology Synthesis Project^21–30^. The project aims to promote an improved understanding of HCV epidemiology and to provide the evidence necessary to guide research, policy, and programmatic efforts in this region.

## Methods

We characterized HCV epidemiology in Egypt through several descriptive, analytical, and quantitative assessments. We first conducted systematic literature reviews to expand and update our previously published review of HCV antibody incidence and prevalence in Egypt^21^ and to describe the diversity in HCV genotype and subtype distributions. We subsequently performed random-effects meta-analyses to estimate the mean anti-HCV prevalence among populations and subpopulations at various risks of exposure to the infection, and the mean HCV viremic rate—that is the mean proportion of individuals chronically infected with HCV (HCV RNA positive) among those antibody positive for HCV. We subsequently constructed and conducted random-effects meta-regression models to identify associations with anti-HCV prevalence among the general population and sources of between-study heterogeneity.

### Systematic review of incidence of HCV infection and anti-HCV prevalence studies

**Figure 1:**
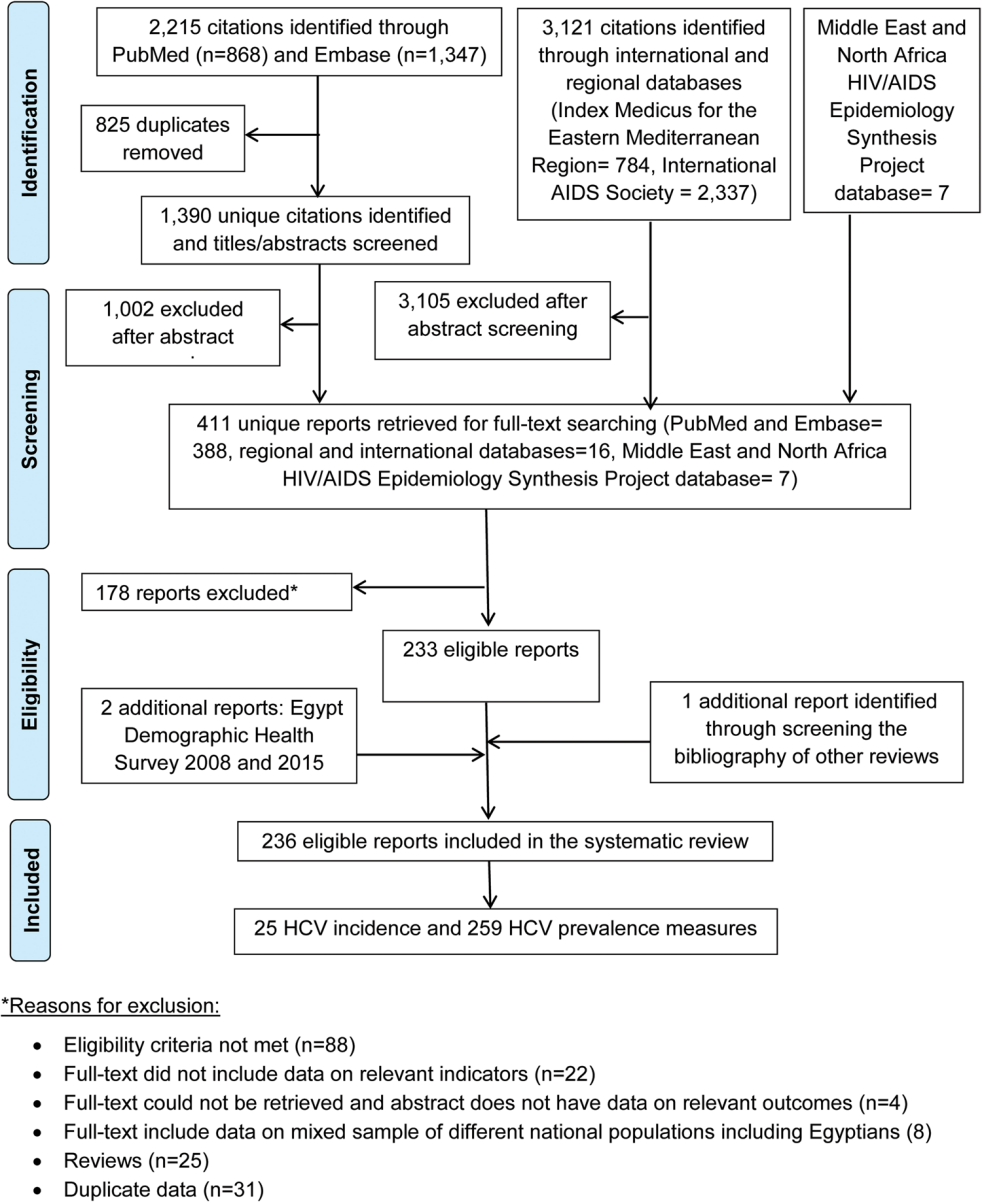
Flow chart of article selection for the systematic review of hepatitis C virus (HCV) antibody incidence and prevalence in Egypt, adapted from the PRISMA 2009 guidelines^32^.

We conducted a systematic review of HCV antibody incidence (Table 1) and prevalence (Supplementary Tables S2–S4) among the Egyptian resident and expatriate populations following the Cochrane Collaboration guidelines^31^ and reported the findings following the PRISMA guidelines^32^ (Fig. 1). The PRISMA checklist is provided in Supplementary Figure S1. We conducted an exhaustive search surveying international and regional databases as well as the grey literature using broad criteria with no language restrictions (Supplementary Figure S2).

**Table 1:** Studies reporting hepatitis C virus (HCV) antibody incidence in Egypt.

**First author, year of publication [citation]**	**Year(s) of data collection**	**Study site**	**Population**	**Sample size at recruitment**	**Lost to follow-up**	**HCV sero-conversion risk (relative to total sample size)**	**HCV incidence rate (per 1,000 person-years)**	**Total person-years**	**Follow-up duration (months)**
**General population (n = 5)**									
Mohamed, 2005^48^	1997–2000	Community	Household members surveyed in Aghour el Soughra village in Nile Delta	2,463	931	—	6.8	4,195	19
Mohamed, 2005^48^	1997–2000	Community	Household members surveyed in Sallam village in Upper Egypt	4,275	2,443	—	0.8	6,720	19
Mostafa, 2010 ^49^	2001–2006	Community	Household members surveyed in 3 villages in Menoufia governorate in Nile Delta	3,580	396	0.79	2.4	10,578	48
Mikhail, 2007^51^	2000–2004	Community	Control subjects surveyed in a village in Nile Delta	149	—	—	10.2	—	10
Saleh, 2008^50^	1997–2006	ANC	Pregnant women surveyed in 3 villages in Menoufia governorate in Nile Delta	2,171	—	—	5.2	4,814	26
**Populations at high risk (n = 7)**									
El-Sherif, 2012^166^	—	Hospital	Hemodialysis patients	14	—	21.4	—	—	4
Goher, 1998^96^	—	Hemodialysis units	Hemodialysis patients on non-reused dialyzers	37	—	21.6	—	—	6
Goher, 1998^96^	—	Hemodialysis units	Hemodialysis patients on reused dialyzers	53	—	20.8	—	—	6
Khodir, 2012^167^	2011	Hemodialysis units	Hemodialysis patients	1,527	—	11	—	—	8
Soliman, 2013^92^	2008–2010	Hospital and hemodialysis units	Hemodialysis patients following strict isolation program	27	—	14.8	—	—	36
Soliman, 2013^92^	2008–2010	Hospital and hemodialysis units	Hemodialysis patients not following strict isolation program	56	—	42.9	—	—	36
Zahran, 2014^89^		Hemodialysis units	Hemodialysis patients	303		14.5	—	—	36.7
**Populations at intermediate risk (n = 4)**									
Abdelwahab, 2013^53^	2008–2011	Hospital	Healthcare workers	717	66	0.3	2.0	—	18
Munier, 2013^52^	2008–2010	Hospital	Healthcare workers	73	—	0	—	—	6
Okasha, 2015^54^	2008	Hospital	Healthcare workers	402	102	1	7.3	551	18
Saleh, 2010^55^	2000–2006	ANC	Children of HCV infected mothers^*^	2,852	353	0.53	2.7	5,573	66
**Populations with liver-related conditions (n = 2)**									
Meky, 2006^168^	2002–2005	Community	Small sub-sample of patients with liver disease biomarkers in a community study	6	—	33.3	—	—	6
Mikhail, 2007^51^	2000–2004	National liver endoscopy unit	Chronic liver disease patients undergoing endoscopy	149	—	2.7	—	—	10
**Special clinical populations (n = 1)**									
Hassan, 2013^56^	—	Hospital	Stem cell transplant patients	50	—	4.0	—	—	—
**Mother-to-child transmission (n = 6)**									
Abdul-Qawi, 2010^57^	2003–2008	Hospital	Infants of HCV Ab+and RNA+mothers	53	—	3.8	—	—	6
Abo Elmagd, 2011^61^	—	—	Infants of HCV Ab+and/or RNA+mothers	8^‡^	—	25	—	—	0-12^ǁ^
El-Sayed Zaki, 2013^62^	2012–2013	Hospital	Infants of HCV Ab+mothers	12	—	8.3	—	—	—
Kassem, 2000^59^	1996	Hospital	Infants of HCV Ab+and RNA+mothers	14	—	36	—	—	0^†^
Kumar, 199^58^	1994–1996	Hospital	Infants of HCV Ab+and RNA+mothers	65	—	24.6	—	—	18
Shebl, 2009^60^	1998–2001	ANC	Infants of HCV Ab+and/or RNA+mothers	232^**^	—	3.4	—	—	24

^*^HCV infection among these children was community-acquired; ^**^7 out of 232 infants were born to mothers who were only HCV RNA positive; ^†^Blood samples collected at birth; ^‡^3 out of 8 infants were born to mothers who were only HCV RNA positive; ^∥^Infants’ ages ranged from 0 to 12 months.

^**^Abbreviations: ANC, antenatal clinic; Ab, antibody; +, positive.

After duplicates’ exclusion, screening of titles and abstracts was performed. Full-text screening of relevant or potentially relevant reports was further conducted. Hand searching of references of literature reviews was also implemented. Any report including primary data for HCV antibody incidence and/or prevalence qualified for inclusion in the review. Case reports, case series, editorials, letters to editors, commentaries, and studies reporting non-A non-B hepatitis were disregarded. Here, a “report” may include one or more outcomes of interest, while a “study” pertains to a specific outcome measure.

Data characterizing the identified HCV measures were extracted by SPK (Supplementary Table S1). Consistency checks for 10% of the extracted reports were performed by HC. Incidence of HCV infection studies, and anti-HCV prevalence studies with at least 50 participants, were synthesized by populations’ risk of exposure to the infection^21,23–30^ as follows:

1. General populations: these include populations at relatively low risk of exposure to HCV such as blood donors, healthy children, antenatal clinic (ANC) attendees, pregnant women, and participants in household-based surveys, among others.
2. Populations at high risk: these include people who inject drugs (PWID), and populations exposed to frequent medical injections and/or blood transfusions such as hemodialysis, thalassemia, hemophilia, and multi-transfused patients, among others.
3. Populations at intermediate risk: these include populations whose risk of exposure is perceived to be higher than the general population, but lower than populations at high risk, such as healthcare workers, household contacts of HCV infected patients, patients with diabetes, and prisoners, among others.
4. Populations with liver-related conditions: these include patients with liver-related conditions of an epidemiological significance to HCV infection such as patients with chronic liver disease, acute viral hepatitis, hepatocellular carcinoma, and liver cirrhosis, among others. This category includes also non-Hodgkin’s lymphoma patients because of potential link to HCV infection^33,34^.
5. Special clinical populations: these include patients whose risk of exposure to HCV is uncertain such as patients with non-liver related malignancies, dermatological manifestations, and rheumatological disorders, among others.
6. Mixed populations: these include samples with a mix of the previously described populations. These were reported, but were excluded from further analyses.

Quality assessment for the identified measures was conducted as informed by the Cochrane Collaboration guidelines^35^ (Supplementary Figure S3 and Supplementary Tables S5–S9).

### Systematic review of HCV genotypes

**Figure 2:**
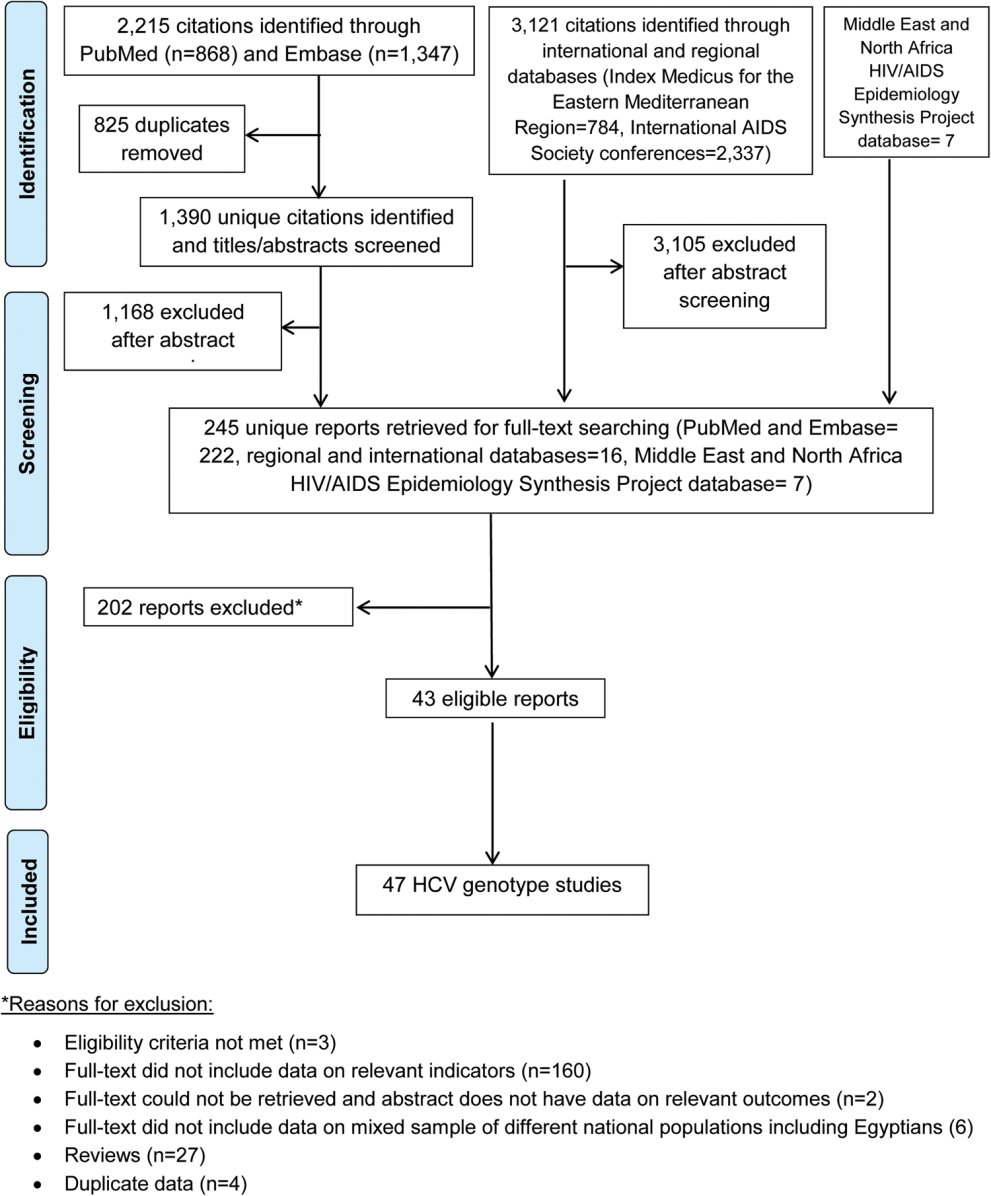
Flow chart of article selection for the systematic review of hepatitis C virus (HCV) genotypes in Egypt, adapted from the PRISMA 2009 guidelines^32^.

**Figure 3:**
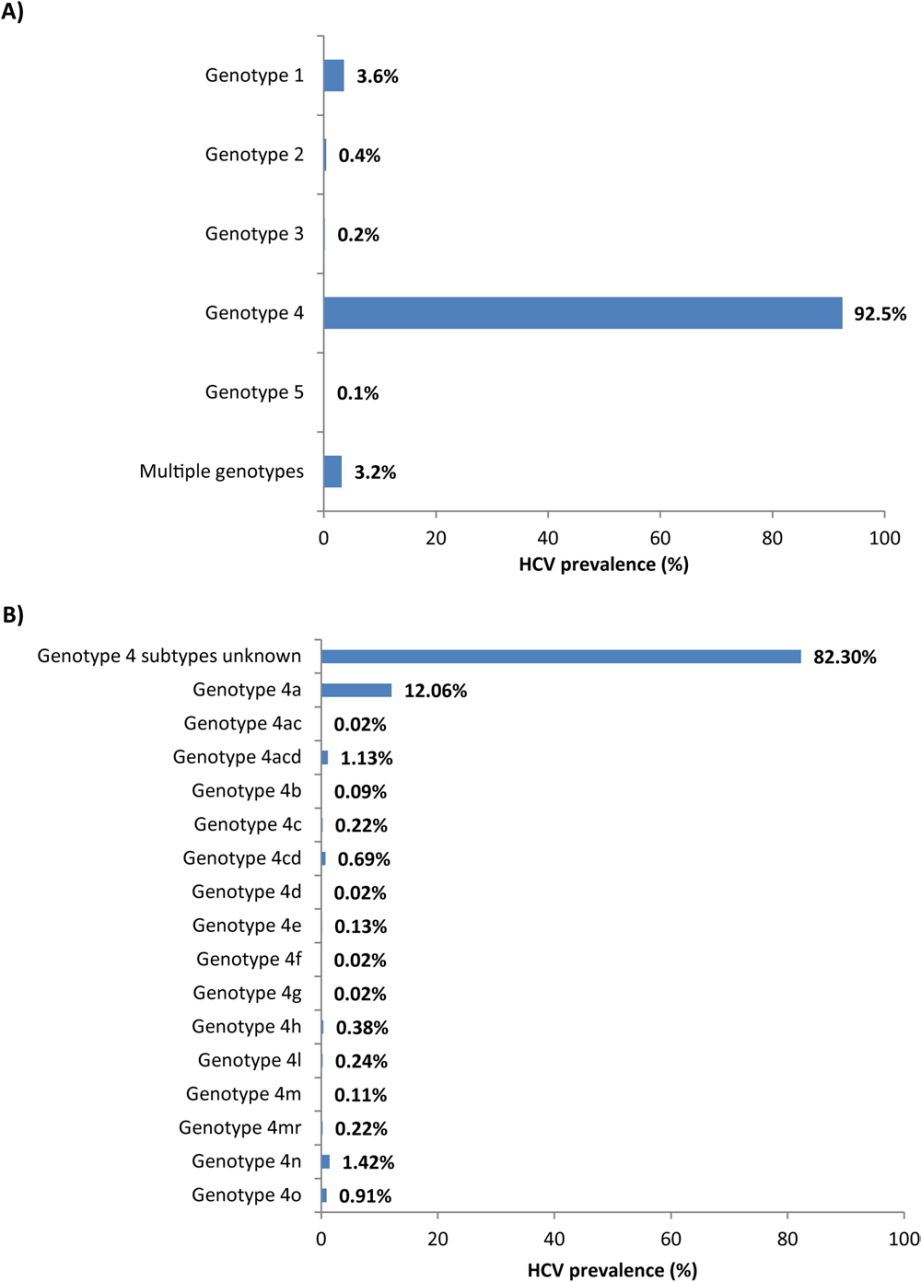
Distribution of hepatitis C virus (HCV) genotypes and subtypes among HCV RNA positive individuals in Egypt. (**A**) Distribution of HCV genotypes among HCV RNA positive individuals. (**B**) Distribution of HCV genotype 4 subtypes as available.

A second independent systematic review for HCV genotypes and subtypes among Egyptians was conducted following the same methodology (Fig. 2) and using the original broad search criteria (Supplementary Figure S2). Genotype, and where available, subtype information were extracted from all relevant studies irrespective of sample size (Supplementary Table S1). Frequency distributions were described (Fig. 3). Genotype diversity was quantified using Shannon Diversity Index^36^. The frequency distribution of genotypes was calculated with individuals testing positive for multiple genotypes contributing separately to each genotype in the distribution^37^ (Table 2).

**Table 2:** Frequency, distribution, and Shannon Diversity Index of identified hepatitis C virus (HCV) genotypes across Egypt.

	n	%
**Genotype 1**	191	4.0
**Genotype 2**	60	1.2
**Genotype 3**	39	0.8
**Genotype 4**	4,735	94.1
**Genotype 5**	6	0.1
**Genotype 6**	—	—
**Genotype 7**	—	—
***Shannon Diversity Index***	0.27	
Index relative to total possible diversity*	14.4%	

^*^The maximum value for Shannon Diversity Index is 1.95 assuming full genotype diversity of seven HCV genotypes^30,37^.

### Meta-analyses of anti-HCV prevalence measures among populations and subpopulations

Meta-analyses were performed to estimate the pooled mean anti-HCV prevalence among populations and subpopulations at various risks of exposure to HCV. Studies including at least 25 individuals qualified for inclusion in meta-analysis. We substituted anti-HCV prevalence for the total sample with stratified measures whenever the required sample size was met at the stratum level. Only one stratification for each study was included in the meta-analysis following a pre-defined sequential order prioritizing: population, sex, year, region, and finally age.

For each meta-analysis, the variances of anti-HCV prevalence measures were first stabilized by implementing Freeman-Tukey type arcsine square-root transformation^38^. The inverse variance was then used to weight anti-HCV prevalence measures which were then pooled into one summary estimate (for mean) using a DerSimonian-Laird random-effects model^39^. Heterogeneity was further assessed^39,40^ (Table 3).

**Table 3:** Pooled mean estimates for hepatitis C virus (HCV) antibody prevalence stratified by populations’ and subpopulations’ risk of exposure to HCV infection in Egypt.

	Studies	Samples	Prevalence	Effect size	Heterogeneity measures			
	Total N	Total N	Range (%)	Mean (%) (95% CI)	Q (p-value)^*^	*τ* ^2**^	I² (confidence limits)^ǂ^	Prediction interval (%)^ǁ^
**All populations**								
General population	264	1,677,404	0.0–57.6	11.9 (11.1–12.6)	38,386.8 (p < 0.0001)	0.0257	99.3% (99.3–99.3%)	3.5–24.0
Populations at high risk	57	7,459	8.8–100	55.6 (49.4–61.7)	1,437.3 (p < 0.0001)	0.2079	96.1% (95.5–96.6%)	13.1–93.6
Populations at intermediate risk	45	9,427	0.0–90.0	14.3 (10.3–18.8)	1,436.2 (p < 0.0001)	0.1553	96.9% (96.4–97.4%)	0–51.0
Populations with liver-related conditions	72	47,214	4.3–100	56.0 (50.4–61.6)	7,777.2 (p < 0.0001)	0.2199	99.1% (99.0–99.2%)	12.8–94.2
Special clinical populations	34	5,542	0.5–96.1	35.0 (27.3–43.1)	1206 (p < 0.0001)	0.2256	97.3% (96.8–97.7%)	1.5–81.8
**General population (populations at low risk)**								
Blood donors	116	1,566,669	0.0–38.0	10.4 (9.6–11.2)	24,513.7 (p < 0.0001)	0.0180	99.5% (99.5–99.6%)	3.6–20.0
Family replacement	15	262,535	3.8–14.6	7.1 (5.9–8.3)	1,931.7 (p < 0.0001)	0.0080	99.3% (99.1–99.4%)	2.8–13.1
Voluntary	27	1,025,535	0.7–27.2	5.4 (4.4–6.5)	9,700.4 (p < 0.0001)	0.0133	99.7% (99.7–99.8%)	1.3–12.2
Pregnant women	14	12,700	2.3–19.0	9.0 (6.0–12.6)	455.0 (p < 0.0001)	0.0405	97.1% (96.2–97.8%)	0.4–26.1
Children	10	3,408	0.0–38.0	6.4 (2.9–11.1)	171.6 (p < 0.0001)	0.0604	94.8% (92.2–96.5%)	0–28.2
Egyptian expatriate workers undergoing mandatory pre-employment screening and Egyptians living abroad	23	9,168	0.0–38.4	14.4 (9.3–20.4)	1137.0 (p < 0.0001)	0.0349	98.1% (97.7–98.4%)	0–50.4
Other general populations	101	85,459	0.0–57.6	14.3 (12.3–16.4)	6472.6 (P < 0.0001)	0.0192	98.5% (98.3–98.6%)	0.9–38.3
**Populations at high risk**								
Hemodialysis patients	26	4,915	10.0–100.0	65.5 (56.5–74.1)	809.1 (p < 0.0001)	0.2119	96.9% (96.2–97.5%)	18.9–98.6
Thalassemia patients	21	1,812	8.8–82.0	46.3 (34.9–57.9)	484.0 (p < 0.0001)	0.2710	95.9% (94.7–96.8%)	2.9–93.9
Multi-transfused patients	6	449	15.2–81.6	42.9 (24.5–62.3)	80.5 (p < 0.0001)	0.2144	93.8% (89.1–96.5%)	0–98.3
Other populations at high risk	4	283	13.0–63.0	57.5 (36.8–76.9)	34.6 (p < 0.0001)	0.0393	91.3% (80.9–96.1%)	0.0–100
**Populations at intermediate risk**								
Healthcare workers	10	3,402	0.0–42.1	8.4 (3.7–14.8)	274.4 (p < 0.0001)	0.0891	96.7% (95.4–97.7%)	0–37.9
Diabetic patients	6	1,384	12–60.3	24.7 (6.0–50.4)	415.1 (p < 0.0001)	0.4330	98.8% (98.3–99.1%)	0–100
Household contacts of HCV infected persons	13	2,339	0–46.0	13.7 (7.6–21.1)	260.2 (p < 0.0001)	0.1197	95.4% (93.6–96.7%)	0–49.3
Hospitalized patients	6	401	0–90.0	15.9 (0–57.8)	411.1 (p < 0.0001)	1.221	98.8% (98.3–99.1%)	0–100
Other populations at intermediate risk	10	1,547	8.4–41.4	15.2 (11.3–19.4)	39.5 (p < 0.0001)	0.0234	77.2% (58.1–87.6%)	4.2–31.0
**Populations with liver-related conditions**								
Hepatocellular carcinoma patients	22	5,553	30.0–95.2	74.0 (67.1–80.3)	506.2 (p < 0.0001)	0.1071	95.9% (94.7–96.8%)	39.4–97.2
Liver disease patients	28	34,727	16.4–100	65.6 (60.9–70.2)	1121.2 (p < 0.0001)	0.0582	97.6% (97.1–98.0%)	40.3–87.0
Liver cirrhosis patients	2	130	56.0–75.4	66.0 (45.9–83.6)	5.50 (p = 0.0190)	0.0172	81.8% (23.1–95.7%)	0.0–100
Viral hepatitis patients	14	5,992	4.3–78.7	18.1 (11.4–25.8)	360.8 (p < 0.0001)	0.1152	96.4% (95.1–97.3%)	0–54.2
Non-Hodgkin’s lymphoma patients	6	812	7.4–50.6	35.3 (17.5–55.4)	149.6 (p < 0.0001)	0.2378	96.7% (94.7–97.9%)	0–96.8
**Special clinical populations**								
Special clinical populations	34	5,542	0.5–96.1	35.0 (27.3–43.1)	1,206 (p < 0.0001)	0.2256	97.3% (96.8–97.7%)	1.5–81.8

^*^Q: the Cochran’s Q statistic, a measure assessing the existence of heterogeneity in effect size. ^**^τ^2^: the estimated between-study variance in the double arcsine transformed proportions of the true effect sizes. The back-transformed *τ*^2^ was not calculated as the methodology to do so is not currently available. ^ǂ^I²: a measure assessing the magnitude of between-study variation that is due to differences in effect size across studies rather than chance. ^∥^Prediction interval: estimates the 95% interval in which the true effect size in a new HCV study will lie.

^**^Abbreviation: CI, confidence interval.

### Meta-analysis of HCV viremic rates

**Figure 4:**
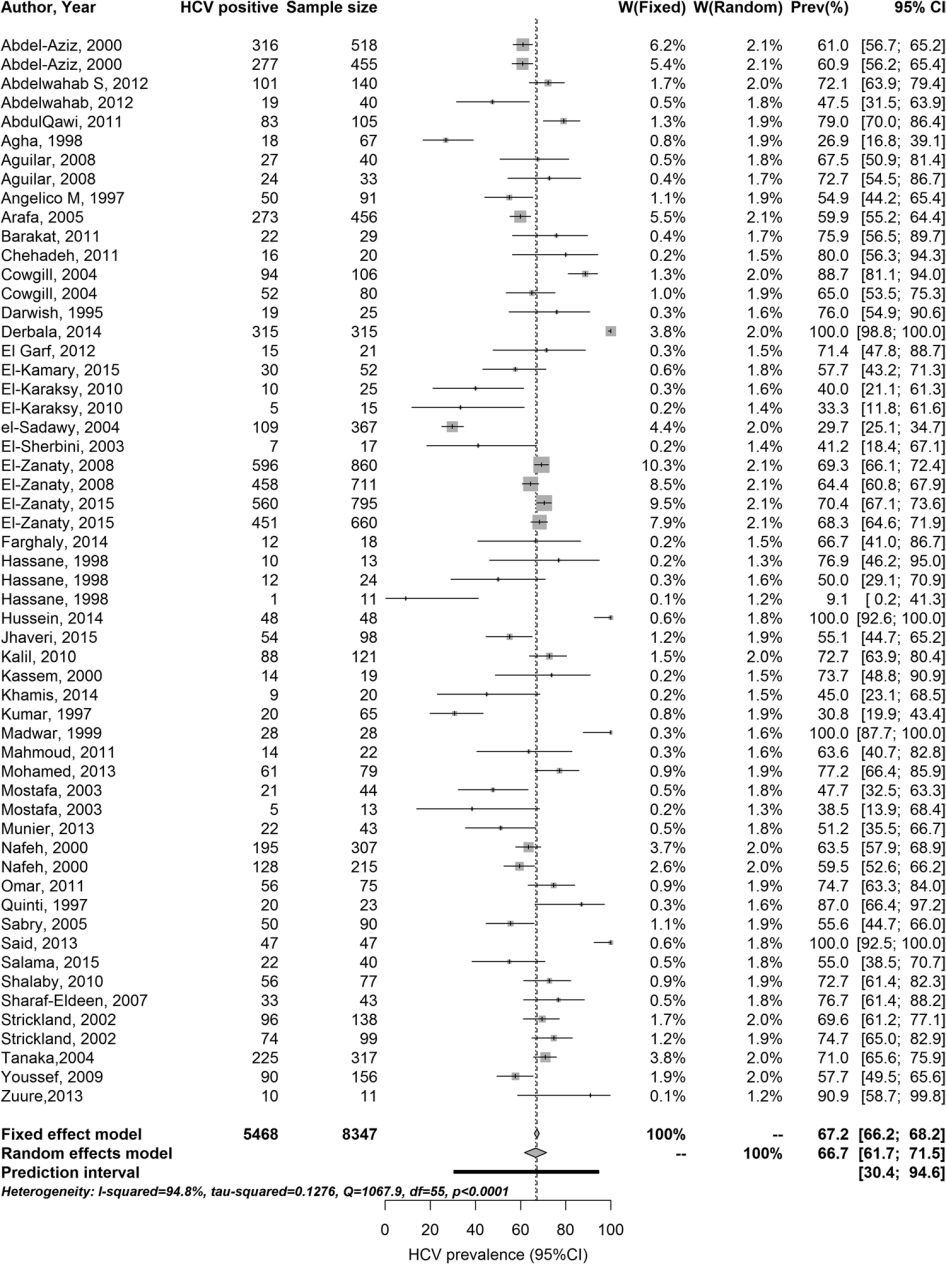
Meta-analysis forest plot for hepatitis C virus (HCV) viremic rate in Egypt. HCV viremic rate is the proportion of individuals chronically infected with HCV (HCV RNA positive) among those antibody positive for HCV.

HCV RNA prevalence measures, identified through the HCV antibody incidence and prevalence systematic review, were synthesized by population’s risk of exposure to HCV (Supplementary Tables S1–S3). An estimate for the mean HCV viremic rate was subsequently generated by pooling measures of HCV RNA positivity among anti-HCV positive persons using a random-effects meta-analysis, following the same protocol applied for the anti-HCV prevalence meta-analyses (Fig. 4).

Meta-analyses were conducted in R version 3.2.2^41^ using the package meta^42^.

### Meta-regression analyses

Meta-regression analyses were performed to identify associations with anti-HCV prevalence among the general population and sources of between-study heterogeneity (Table 4). Potential predictors were specified a priori and included: subpopulation type (blood donors; pregnant women/ANC attendees; children; Egyptian expatriate workers mandatory pre-employment screening; other general population groups), region (Upper Egypt; Middle Egypt; Lower Egypt; Canal and Sinai; national; mixed regions; regions outside Egypt; unspecified), study site (blood bank; national; clinical; others; unspecified), sampling methodology (probability-based; non-probability based), sample size (<100; >=100), publication year, and median year of data collection. Associations were described using odds ratios (ORs), 95% confidence intervals (CIs) and p-values. Factors with a p-value < 0.1 in univariable analyses were eligible for inclusion in the initial multivariable model. Factors with a p-value < 0.05 were retained in the final model.

**Table 4:** Univariable and multivariable meta-regression models for HCV antibody prevalence among the general population in Egypt.

		Number of studies	Univariable analyses		Multivariable analysis^*^	
			OR (95% CI)	p-value^**^	AOR (95% CI)	p-value^‡^
Subpopulation type (among the general population)	Blood donors	111	1		1	
	Pregnant women/ANC attendees	13	0.86 (0.46–1.63)	0.651	1.11 (0.62–1.96)	0.729
	**Children**	16	0.40 (0.22–0.71)	0.002^**^	0.47 (0.27–0.79)	0.005^‡^
	Egyptian expatriate workers undergoing mandatory pre-employment screening and Egyptians living abroad	23	1.45 (0.88–2.37)	0.141	0.98 (0.43–2.21)	0.956
	**Other general population groups**	99	1.47 (1.09–1.98)	0.012^**^	1.63 (1.17–2.28)	0.004^‡^
Region	Upper Egypt	9	1		1	
	Middle Egypt	54	1.69 (0.78–3.67)	0.186	1.57 (0.79–3.12)	0.199
	Lower Egypt	89	2.42 (1.14–5.14)	0.022^**^	1.92 (0.98–3.75)	0.057
	Canal & Sinai	16	1.65 (0.67–4.06)	0.271	0.62 (0.27–1.40)	0.247
	National	24	1.48 (0.64–3.45)	0.357	0.86 (0.36–2.06)	0.735
	Mixed regions	39	4.00 (1.80–8.87)	0.001^**^	1.85 (0.80–4.27)	0.150
	Regions outside Egypt	15	2.58 (1.04–6.41)	0.041^**^	2.41 (0.75–7.71)	0.139
	Unspecified	16	3.24 (1.32–7.96)	0.011^**^	1.62 (0.73–3.63)	0.236
Study site	Blood bank	77	1			
	National	24	0.68 (0.40–1.14)	0.143	—	—
	Clinical	72	1.04 (0.72–1.49)	0.844	—	—
	Others	74	1.08 (0.75–1.56)	0.663	—	—
	Unspecified	15	1.56 (0.83–2.92)	0.167	—	—
Sampling methodology	Probability-based	66	1			
	Non-probability based	196	0.72 (0.53–0.99)	0.044^**^	0.70 (0.46–1.05)	0.086
Sample size	<100	63	1			
	>=100	199	0.73 (0.53–1.00)	0.052^**^	1.14 (0.85–1.54)	0.385
Publication year		262	0.93 (0.92–0.95)	0.000^**^	0.94 (0.92–0.96)	0.000^‡^
Median year of data collection		197	1.0	0.458	—	—

^*^Total between-study variation explained by final multivariable model: 29.0%.

^**^Factors with p-value < 0.1 were eligible for inclusion in the multivariable model.

^‡^Factors with p-value < 0.05 were considered statistically significant.

^†^Abbreviations: AOR, adjusted odds ratio; OR, odds ratio; CI, confidence interval.

Meta-regressions were conducted in STATA version 12^43^ using the package metareg^44^.

## Results

### Systematic review of HCV antibody incidence and prevalence: study selection process

Figure 1 shows the selection process for HCV antibody incidence and prevalence studies following PRISMA guidelines^32^. A total of 5,343 citations were identified (868 using PubMed, 1,347 using Embase, 3,121 using other international and regional databases, and 7 using the MENA HIV/AIDS Epidemiology Synthesis Project database^45,46^). Out of these, 411 reports were considered relevant or potentially relevant after duplicates’ removal and titles and abstracts’ screening. A final of 233 reports qualified for inclusion in the systematic review after full-texts’ screening. The rest were excluded for different reasons outlined in Fig. 1. Two EDHS reports^11,12^, and an additional report^47^ identified through hand-searching other reviews’ bibliography, were also included.

To sum, the systematic review included 236 eligible reports reporting a total of 25 incidence of HCV infection studies and 259 anti-HCV prevalence studies. We report the decimal places of the prevalence and incidence figures as in the original report, but we rounded the prevalence and incidence figures with more than one decimal places to one decimal place.

### Overview of incidence of HCV infection

All of the 25 incidence of HCV infection studies were based on a prospective cohort design (Table 1). Only a third reported HCV incidence rate, while the rest reported seroconversion risk for anti-HCV.

Five studies were conducted among the general population. These reported incidence rates ranging between 0.8 per 1,000 person-years among residents of a village in Upper Egypt, and 10.2 per 1,000 person-years among residents of a village in the Nile Delta^48–51^. The seven studies among populations at high risk, all among hemodialysis patients, reported seroconversion risks for anti-HCV ranging between 11% and 42.9%. Four studies were conducted among populations at intermediate risk, three of which were among healthcare workers^52–54^ and one was among children of HCV infected mothers^55^. Among healthcare workers, the first study reported no incidence of HCV infection^52^, while the other two reported rates of 2.0^53^ and 7.3^54^ per 1,000 person-years. The study among children who were HCV negative at birth but had HCV infected mothers reported a rate of 2.7 per 1,000 person-years^55^. A single incidence study was conducted among a special clinical population and reported a seroconversion risk for anti-HCV of 4% among stem cell transplant patients^56^.

Six incidence studies documented mother-to-child transmission. Out of these, three were conducted among infants born to anti-HCV positive and RNA positive mothers and reported a seroconversion risk for anti-HCV of 3.8%^57^, 24.6%^58^, and 36%^59^. Two other studies among infants born to anti-HCV positive and/or HCV RNA positive mothers reported a seroconversion risk for anti-HCV of 3.4%^60^ and 25%^61^. One study among infants born to anti-HCV positive mothers assessed the seroconversion risk for anti-HCV at 8.3%^62^.

### Overview of anti-HCV prevalence

Of the 259 anti-HCV prevalence studies, 117 were among the general population (Supplementary Table S2), 35 among populations at high risk (Supplementary Table S3), 32 among populations at intermediate risk (Supplementary Table S4), 45 among populations with liver-related conditions (Supplementary Table S4), 27 among special clinical populations (Supplementary Table S4), and 3 among mixed populations (Supplementary Table S4).

Among the general population (Supplementary Table S2), anti-HCV prevalence varied from 0–51% with a median of 13.0%. High prevalence levels were reported among subpopulations including blood donors (range = 1.6–34%; median = 9.2%), participants in household-based surveys and village residents (range = 0–51%; median = 14.3%), children (range = 0–38%; median = 5.8%), pregnant women and ANC attendees (range = 3.9–19%; median = 10.8%), study controls (range = 0–49.3%; median = 30.2%), and other general population groups (range = 0–50%; median = 18.9%).

Among populations at high risk (Supplementary Table S3), anti-HCV prevalence was high ranging from 8.9–98% with a median of 51.7%. High prevalence levels were reported among subpopulations such as hemodialysis patients (range = 27.1–98%; median = 67.5%), multi-transfused patients (range = 17.6–54.9%; median = 38.2%), and thalassemia patients (range = 8.9–82%; median = 50.0%). A single study among hemophilic children reported anti-HCV prevalence at 40.0%^63^. The only study among PWID assessed anti-HCV prevalence at 63%^64^.

Among populations at intermediate risk (Supplementary Table S4), anti-HCV prevalence varied from 0–90% with a median of 12.6%. The prevalence also varied among subpopulations including healthcare workers (range = 0–16.6%; median = 7.5%), household contacts of index patients (range = 0–35.5%; median = 13.7%), and diabetic patients (range = 3.6–60.3%; median = 18.0%). Anti-HCV prevalence was further measured at 15.8%^65^ and 31.4%^66^ among prisoners, at 8.4%^67^ and 10%^68^ among patients with sexually transmitted infections (STIs), at 0%^69,70^ and 90%^71^ among hospitalized patients, and at 12.5%^72^ among barbers and their clients.

Among populations with liver-related conditions (Supplementary Table S4), studies reported anti-HCV prevalence in the range of 4.3–100% with a median of 58.2%. Anti-HCV prevalence was measured among acute viral hepatitis patients (range = 4.3–29%; median = 12.0%), chronic liver disease patients (range = 38.6–100%; median = 69.2%), and hepatocellular carcinoma patients (range = 30–95.2%; median = 76.0%). Among special clinical populations (Supplementary Table S4), anti-HCV prevalence ranged from 6.7–96.1% with a median of 38.0%.

### Quality appraisal for HCV antibody incidence and prevalence studies

Summary findings of our quality appraisal for HCV antibody incidence and prevalence studies can be found in Supplementary Table S5. Details of the quality appraisal for each individual study are included in Supplementary Tables S6–S9.

Briefly, about half of incidence of HCV infection (44.0%) and the majority of anti-HCV prevalence (76.4%) studies included at least 100 participants, and hence were considered as having high precision. Almost all incidence of HCV infection and anti-HCV prevalence measures were based on convenience sampling, and hence were considered as having high risk of bias (ROB) for this domain. The majority of incidence of HCV infection (92.0%) and anti-HCV prevalence (88.0%) studies specified the type of assays used for infection ascertainment. The majority of incidence of HCV infection studies (75.0%) with information on infection ascertainment relied on recent ELISA tests with higher sensitivity and specificity (3^rd^ generation or more). Meanwhile, only 27.6% of anti-HCV prevalence studies with such information reported using 3^rd^ or 4^th^ generation assays. Nearly two thirds of studies had missing information on the response rate domain.

Incidence of HCV infection and anti-HCV prevalence studies were overall of acceptable quality. The majority of studies (88.0%) were classified as having low ROB in one or more quality domain, and almost half (48.6%) as having low ROB in two or more quality domains. High ROB in two or more quality domains was found in only 6% of studies.

### Risk factors for incident HCV infection and factors associated with being infected with HCV infection

A total of 53 studies assessed risk factors for incidence of HCV infection or factors associated with being infected with HCV infection, using multivariable logistic regression analyses. Commonly reported were mass parenteral antischistosomal treatment (PAT) campaign exposure^54,66,73–88^ and other healthcare exposures such as dialysis^89–97^, transfusion^54,57,59,70,77–80,85,86,91,93,95,98–107^, surgery^47,79,84,101–103,108^, frequent injections^78,86,103,109^, and dental procedures^65,78,86,88,101,102,108,110^. Exposures at the workplace were reported for healthcare workers^54,111^. Studies further linked HCV infection to unsafe practices in the community such as using contaminated needles or sharp objects during tattooing^65,101^, ear piercing^65,101^, male circumcision by traditional healers^101^, female circumcision (female genital mutilation)^112^, *hijama* (cupping)^74^, or while shaving at community barbers^47,79,113^. Other documented risk factors include inter-familial contacts^48,57,89,108^ and mother-to-child (vertical) transmission^57–62^.

### Systematic review of HCV genotypes

Figure 2 describes the selection process for HCV genotype studies following PRISMA guidelines^32^. Out of the total 5,343 citations identified through screening international and regional data sources, 245 reports were considered relevant or potentially relevant after duplicates’ removal and titles and abstracts’ screening. Following full-text screening, 43 reports qualified for inclusion in the systematic review yielding a total of 47 genotype studies on 4,783 HCV RNA positive individuals.

A single HCV strain was isolated from the vast majority of infected individuals; only 3.2% were infected with multiple genotypes (Fig. 3A). Individuals were predominantly infected with genotype 4 regardless of population type. Genotype 4 was found among blood donors^114–117^, pregnant women^118^, village residents^76^, outpatients^119^, Egyptian expatriate general populations^75,120^, thalassemia patients^121^, hemodialysis patients^122^, healthcare workers^123,124^, children of HCV infected mothers^60^, household contacts of HCV infected individuals^52^, hospitalized populations^125^, diabetic patients^126^, hepatocellular carcinoma patients^127^, chronic liver disease patients^83,125,128,129^, acute viral hepatitis patients^130–132^, special clinical populations^133–139^, and mixed populations^140–142^. Studies also identified a wide array of subtypes for genotype 4 (Fig. 3B), with subtype 4a being the most frequently reported (12.1%).

HCV genotype distribution (Table 2) indicated the following frequency order: genotype 4 (94.1%), genotype 1 (4.0%), genotype 2 (1.3%), genotype 3 (0.8%), and genotype 5 (0.1%). A single study reported a case of chronic active hepatitis with genotype 10a^133^—a rare finding^37^ that was excluded from further analyses. Genotype diversity was low with a relative Shannon Diversity Index of only 14.4% (score: 0.27 out of a maximum of 1.95).

### Estimates for the pooled mean anti-HCV prevalence among populations and subpopulations

Results of our meta-analyses for anti-HCV prevalence (Table 3), based on pooling available measures, indicated a mean prevalence of 11.9% (95% CI = 11.1–12.6%) among general populations, 55.6% (95% CI = 49.4–61.7%) among populations at high risk, 14.3% (95% CI = 10.3–18.8%) among populations at intermediate risk, 56.0% (95% CI = 50.4–61.6%) among populations with liver-related conditions, and 35.0% (95% CI = 27.3–43.1%) among special clinical populations.

Pooled mean anti-HCV prevalence was estimated for subpopulations of the general population at 10.4% (95% CI = 9.6–11.2%) among all blood donors, 7.1% (95% CI = 5.9–8.3%) among family replacement donors, 5.4% (95% CI = 4.4–6.5%) among voluntary blood donors, 9.0% (95% CI = 6.0–12.6%) among pregnant women, 6.4% (95% CI = 2.9–11.1%) among children, 14.4% (95% CI = 9.3–20.4%) among Egyptian expatriate workers undergoing mandatory pre-employment screening and Egyptians living abroad, and 14.3% (95% CI = 12.3–16.4%) among other general populations.

Anti-HCV prevalence was high across subpopulations at high risk with a pooled mean of 65.5% (95% CI = 56.5–74.1%) among hemodialysis patients, 46.3% (95% CI = 34.9–57.9%) among thalassemia patients, 42.9% (95% CI = 24.5–62.3%) among multi-transfused patients, and 57.5% (95% CI = 36.8–76.9%) among other high-risk subpopulations.

Pooled mean anti-HCV prevalence among subpopulations at intermediate risk was estimated at 8.4% (95% CI = 3.7–14.8%) among healthcare workers, 24.7% (95% CI = 6.0–50.4%) among diabetic patients, 13.7% (95% CI = 7.6–21.1%) among household contacts of HCV infected individuals, 15.9% (95% CI = 0–57.8%) among hospitalized patients, and 15.2% (95% CI = 11.3–19.4%) among the remaining intermediate-risk subpopulations.

Pooled mean anti-HCV prevalence among subpopulations with liver-related conditions was estimated at 74.0% (95% CI = 67.1–80.3%) among hepatocellular carcinoma patients, 65.6% (95% CI = 60.9–70.2%) among liver disease patients, 66.0% (95% CI = 45.9–83.6%) among liver cirrhosis patients, 18.1% (95% CI = 11.4–25.8%) among viral hepatitis patients, and 35.3% (95% CI = 17.5–55.4%) among non-Hodgkin’s lymphoma patients (included as “liver-related” because of potential link to HCV infection^33,34^).

Considerable heterogeneity in effect size was observed across all meta-analyses (Cochrane’s Q statistic’s p-value < 0.0001), with the effect size (HCV prevalence) prediction intervals generally being wide. Most of the variability across studies was due to true differences across studies rather than chance (I^2^ > 77.2%; Table 3).

### HCV RNA prevalence overview and pooled mean estimate for HCV viremic rate

Our search identified 77 HCV RNA measures, of which 53 were among anti-HCV positive individuals (Supplementary Tables S2–S4). HCV viremic rate (HCV RNA prevalence among anti-HCV positive individuals) ranged from 9.1–100% with a median of 66.7%. Meanwhile, HCV RNA prevalence in samples including individuals irrespective of their antibody status ranged from 0–86.6% with a median of 24.9%.

HCV viremic rate was reported among the general population (range = 26.9–100%; median = 65.3%; Supplementary Table S2) and its subpopulations including children (range = 33.3–75.9%; median = 41.0%), pregnant women (range = 26.9–79.0%; median = 55.1%), and other general populations (range = 29.7–100%; median = 69.5%). Among populations at high risk (Supplementary Table S3), HCV viremic rate was reported among thalassemia patients (range = 55–100%; median = 74.3%), and was measured at 100%^143^ among multi-transfused children, and at 47.5%^63^ among hemophilic children.

HCV viremic rate was reported among patients with liver-related conditions in the range of 55.0–88.7% with a median of 69.6% (Supplementary Table S4), such as patients with acute viral hepatitis (87.0%)^132^, chronic liver disease (55.0%^83^ and 69.6%^80^), non-Hodgkin’s lymphoma (88.7%)^144^, and liver complaints (57.7%)^139^. It was also reported in the range of 9.1–100% with a median of 69.4% among populations at intermediate risk, and in the range of 38.5–76.8% with a median of 59.6% among special clinical populations (Supplementary Table S4).

Figure 4 shows the forest plot for the meta-analysis of HCV viremic rate in Egypt. The pooled mean viremic rate was estimated at 66.7% (95% CI = 61.7–71.5%). Substantial heterogeneity was detected across studies (I^2^ = 94.8%; Cochrane’s Q statistic’s p-value < 0.0001), with an effect size (HCV viremic rate) prediction interval ranging from 30.4–94.6%.

### Associations with anti-HCV prevalence among the general population and sources of heterogeneity across studies

Table 4 shows the results of our meta-regression models assessing the predictors of anti-HCV prevalence among the general population. Our univariable meta-regression analyses identified subpopulation type, region, sampling methodology, sample size, and publication year as significant and eligible for inclusion in the multivariable meta-regression model. The latter showed that, compared to blood donors, children had 53% lower odds for anti-HCV prevalence (adjusted odds ratio (AOR) = 0.47; 95% CI = 0.27–0.79), while other general population groups had 63% higher odds (AOR = 1.63; 95% CI = 1.17–2.28). Our results further indicated 6% lower odds for anti-HCV prevalence for each one year increment in publication year (AOR = 0.94; 95% CI = 0.92–0.96). All other examined predictors were not retained in the final multivariable model, though Lower Egypt as a region and non-probability sampling as a study sampling methodology were, with borderline statistical significance, positively and negatively associated, respectively, with anti-HCV prevalence.

## Discussion

We presented a comprehensive characterization of HCV epidemiology in Egypt by, first, updating and expanding our earlier systematic review^21^ of HCV antibody incidence and prevalence. The updated and expanded review affirmed high HCV infection levels across all populations (Supplementary Tables S2–S4) with evidence for ongoing transmission (Table 1). We also conducted a systematic review of HCV genotypes that highlighted the limited diversity of genotypes in Egypt and the predominance of genotype 4, which alone accounted for 94% of HCV infections (Table 2 and Fig. 3). Our meta-analyses for the pooled mean anti-HCV prevalence among populations and subpopulations at various risks of exposure quantified prevalence levels across diverse population strata and suggested that most HCV exposures are linked to medical care (Table 3). Our meta-analysis for HCV viremic rate indicated that 67% of those anti-HCV positive in Egypt are chronically infected and in need of treatment (Fig. 4). Lastly, our meta-regression analyses highlighted a declining trend for anti-HCV prevalence in Egypt suggesting a rapidly contracting HCV epidemic in this country (Table 4).

One of the key public health questions in Egypt today is whether incidence of HCV infection is ongoing and at what level^21^. Our updated and expanded systematic review identified 25 HCV incidence measures (Table 1), out of which only five were identified in our previous review^21^. Incidence levels of HCV infection were relatively high, even among the general population, confirming endemic transmission of HCV in Egypt. High seroconversion risks for anti-HCV reaching up to 100% were found among clinical populations, namely hemodialysis patients, indicating exposure through medical care. However, some of these incidence studies may have been outdated, while others were conducted in specific locations thus potentially limiting their representativeness of current HCV incidence in the population at large.

This being said, a main finding of our study is the statistically significant decline in anti-HCV prevalence in the general population year by year (Table 4). The decrease in the AOR of 6% per year suggests a rapid decline in incidence of HCV infection, consistent with a contracting HCV epidemic in Egypt. Recent work modeling the Egypt epidemic also supports this finding^5^. Here, HCV incidence rate was estimated at 110 per 100,000 person-years in 2015, and was projected to decline by 33% over the next decade (even assuming no interventions) to reach 74 per 100,000 person-years in 2030^5^. These findings suggest that the improvements in blood screening and infection control over the last two decades have made an impact on reducing HCV transmission in Egypt. In a context of a rapidly scaled-up DAA treatment program^18^, and its impact as treatment for prevention^5^, Egypt will likely progress robustly towards HCV elimination by 2030, after enduring an epidemic of historic proportion.

Another key public health question in Egypt today is determining anti-HCV prevalence among different populations and identifying who should be tested for HCV in Egypt. A total of 259 anti-HCV prevalence measures were identified in the present review (Supplementary Tables S2-S4), compared to only 103 in our earlier review^21^. Despite the sizable addition, findings of both reviews, as well as the meta-analyses (Table 3), converged to the same conclusion of a generalized HCV epidemic in Egypt. HCV prevalence levels were considerably higher than global levels^2,3,13,145^ across all population groups, even those conventionally at low risk of exposure such as pregnant women (Table 3). High mean anti-HCV prevalence was also found among children confirming recent ongoing transmission (Table 3). Pooled estimates for mean anti-HCV prevalence among the different clinical populations were substantial, with about half of these populations being HCV antibody positive (Table 3).

These findings suggest that clinical populations should be prioritized for HCV screening and treatment. Screening programs targeting other populations such as healthcare workers, household contacts of HCV infected patients, and populations at lower risk such as blood donors, pregnant women, and children, may not be as cost-effective and should have lower priority for the time being. As resources are freed and available, screening programs for these populations should be gradually considered.

Our review identified risk factors for incidence of HCV infection and factors associated with being infected with HCV infection. These factors supported healthcare exposures as drivers of HCV transmission such as dialysis^89–97^, blood transfusion^54,57,59,70,77–80,85,86,91,93,95,98–107^, surgery^47,79,84,101–103,108^, and even minor procedures such as dental work^65,78,86,88,101,102,108,110^. Our review further suggested a role for community-related exposures^47,65,74,79,101,112,113^ including informal healthcare practices such as male circumcision by traditional healers^101^, female circumcision^112^, and *hijama* (cupping)^74^. Mother-to-child transmission has been also documented^57–62^—a finding in line with a recent estimate of 3,000-5,000 HCV infections per year through vertical transmission in Egypt^146^.

Among other key public health issues in Egypt today is the contribution of HCV infection to liver disease. Our results show that two thirds of patients with liver-related conditions are infected with HCV (Table 3). The high pooled estimates for the mean anti-HCV prevalence among different populations with liver-related conditions such as hepatocellular carcinoma, liver cirrhosis, and liver disease patients (Table 3) support the prominent role that HCV plays in liver disease incidence in Egypt.

Our pooled estimate of 12% for the mean anti-HCV prevalence among the general population (Table 3) is in line with EDHS findings of a national prevalence of 14.7%^11^ in 2008 and 10.0%^12^ in 2015. Of notice, however, that these EDHS estimates include only the adult population aged 15–59 years of age and our pooled estimate encompass all HCV prevalence data from all years and ages—including those older than 60 years of age. The latter age group is the most affected by the HCV epidemic in Egypt^21,147–149^.

The diversity of HCV genotypes was limited compared to international levels^37,145,150^, quantified at only 14.4% (Table 2) using Shannon’s Diversity Index^36^. Genotype 4 was by far the most dominant accounting for >90% of infections, with limited presence of other genotypes (Fig. 3A and Table 2). Some diversity was observed across subtypes of genotype 4, with subtype 4a being the most frequently reported (Fig. 3B). These findings are consistent with recent global reviews of HCV genotypes that reported on Egypt^37,145,150^, but our findings are based on a much larger sample size pooled through 47 studies. The rather limited diversity in HCV genotypes in Egypt contrasts with what is seen in other MENA countries, including neighboring ones, such as in the Fertile Crescent and Iran where diversity ranged from as low as 35% in Jordan to as high as 66% in Lebanon^29,30^.

The dominance of genotype 4 affirms the special nature of the Egyptian epidemic and its link to a cohort effect^148,149^ and specific events, such as PAT campaigns and more generally healthcare practices before the discovery of the virus^20,149,151,152^. The presence however of other genotypes, and some diversity in genotype 4 subtypes, may suggest the existence of different transmission networks in Egypt that need to be elucidated.

HCV viremic rate can inform HCV estimates and likelihood of identifying chronic infection in different populations. Importantly, it can also play an important role over the next 13 years in monitoring scale-up of HCV treatment coverage and progress towards HCV elimination by 2030. Our findings showed that 67% of anti-HCV positive Egyptians are chronically infected (Fig. 4). This estimate is lower than what is found in the USA, where the viremic rate was estimated at 74% (Chemaitelly, unpublished work) by pooling several rounds of the National Health and Nutrition Examination Survey (NHANES), a nationally representative population-based survey^153^. It is not likely that this difference in viremic rate is explained by differential treatment coverage, since mass HCV treatment has been launched very recently^5^. It is possible, however, that this difference may reflect differential modes of exposure—HCV transmission in the USA is driven largely by injecting drug use^2,3,13^, while the epidemic in Egypt is generalized and is driven mainly by medical care. Other possible explanations include differences in HCV spontaneous clearance rates resulting from differences in HCV circulating genotypes^154^, other host and virus factors^155–159^, and female sex^154,160,161^. The latter is likely to be under-represented in epidemics driven primarily by injecting drug use^162^.

Our study is limited by the quantity and quality of available evidence for specific populations. In particular, only one study was identified for PWID^64^. With a PWID population proportion of 0.16% in Egypt^22,162,163^, injecting drug use could be an important driver of current HCV incidence. Its relative contribution is likely also to grow as the generalized epidemic contracts further over the coming years and becomes a concentrated epidemic in specific populations. Increasing evidence supports a growing role for injecting drug use as a driver of new HCV infections in Egypt^164,165^.

For some of the incidence studies, only seroconversion risk for anti-HCV was reported. Such studies, especially the older ones, may have used less sensitive assays to assess HCV exposure. This may bias the estimated risk of seroconversion in these studies—in such high HCV prevalence settings false negatives may have been identified erroneously as seroconverters.

In conclusion, Egypt is challenged with a high anti-HCV prevalence in virtually all population groups and strata, with evidence for some ongoing HCV transmission. Medical care seems to be the primary source of past and present HCV transmission, with about half of individuals belonging to the clinical populations at high risk being infected. Genotype diversity is low with Genotype 4 being (by far) the dominant genotype. Two thirds of anti-HCV positive Egyptians are chronically infected and in need of treatment. Despite the large scale epidemic in Egypt, HCV antibody incidence and prevalence appear to be declining rapidly consistent with a contracting epidemic. With the recent progress in scaling up Egypt’s DAA treatment program, Egypt is likely to make ambitious strides towards HCV elimination by 2030 after enduring an epidemic of historic proportion.

## Electronic supplementary material

Supplementary information
