# Supplementary material for: Characterizing hepatitis C virus epidemiology in Egypt: systematic reviews, meta-analyses, and meta-regressions
Source: Sci Rep. 2018 Jan 26;8:1661. doi: 10.1038/s41598-017-17936-4 (PMC5785953; doi:10.1038/s41598-017-17936-4)
Supplement: Supplementary file 1 — Supplementary information [file 41598_2017_17936_MOESM1_ESM.pdf]

SUPPLEMENTARY INFORMATION

Characterizing hepatitis C virus epidemiology in Egypt: systematic reviews, meta-analyses, and meta-regressions

Silva P. Kouyoumjian,[1] Hiam Chemaitelly,[1] and Laith J. Abu-Raddad[1, 2]

[1]*Infectious Disease Epidemiology Group, Weill Cornell Medicine-Qatar, Cornell University, Qatar Foundation - Education City, Doha, Qatar*

[2]*Department of Healthcare Policy & Research, Weill Cornell Medicine, Cornell University, New York, USA*

Reprints or correspondence: Prof. Laith J. Abu-Raddad, Infectious Disease Epidemiology Group, Weill Cornell Medicine-Qatar, Qatar Foundation - Education City, P.O. Box 24144, Doha, Qatar. Telephone: + (974) 4492-8321. Fax: + (974) 4492-8333. E-mail: lja2002@qatar-med.cornell.edu

Supplementary Figure S1. Preferred Reporting Items for Systematic Reviews and Meta-analyses (PRISMA) checklist[1].

Section/topic	#	Checklist item	Reported in main text on
**TITLE**			
Title	1	Identify the report as a systematic review, meta-analysis, or both.	p.1
**ABSTRACT**			
Structured summary	2	Provide a structured summary including, as applicable: background; objectives; data sources; study eligibility criteria, participants, and interventions; study appraisal and synthesis methods; results; limitations; conclusions and implications of key findings; systematic review registration number.	p.2
**INTRODUCTION**			
Rationale	3	Describe the rationale for the review in the context of what is already known.	p.3-4
Objectives	4	Provide an explicit statement of questions being addressed with reference to participants, interventions, comparisons, outcomes, and study design (PICOS).	p.3-4
**METHODS**			
Protocol and registration	5	Indicate if a review protocol exists, if and where it can be accessed (e.g., Web address), and, if available, provide registration information including registration number.	p.4
Eligibility criteria	6	Specify study characteristics (e.g., PICOS, length of follow-up) and report characteristics (e.g., years considered, language, publication status) used as criteria for eligibility, giving rationale.	p.5
Information sources	7	Describe all information sources (e.g., databases with dates of coverage, contact with study authors to identify additional studies) in the search and date last searched.	p.5 and Supplementary Figure S2
Search	8	Present full electronic search strategy for at least one database, including any limits used, such that it could be repeated.	Supplementary Figure S2
Study selection	9	State the process for selecting studies (i.e., screening, eligibility, included in systematic review, and, if applicable, included in the meta-analysis).	p.5-7
Data collection process	10	Describe method of data extraction from reports (e.g., piloted forms, independently, in duplicate) and any processes for obtaining and confirming data from investigators.	p.5-6 and Supplementary Table S1
Data items	11	List and define all variables for which data were sought (e.g., PICOS, funding sources) and any assumptions and simplifications made.	p.5-6 and Supplementary Table S1
Risk of bias in individual studies	12	Describe methods used for assessing risk of bias of individual studies (including specification of whether this was done at the study or outcome level), and how this information is to be used in any data synthesis.	p.6 and Supplementary Figure S3
Summary measures	13	State the principal summary measures (e.g., risk ratio, difference in means).	p.7-8
Synthesis of results	14	Describe the methods of handling data and combining results of studies, if done, including measures of consistency (e.g., I^2) for each meta-analysis.	p.5-8
Risk of bias across studies	15	Specify any assessment of risk of bias that may affect the cumulative evidence (e.g., publication bias, selective reporting within studies).	p.6 and Supplementary Figure S3
Additional analyses	16	Describe methods of additional analyses (e.g., sensitivity or subgroup analyses, meta-regression), if done, indicating which were pre-specified.	p.8
**RESULTS**			

Study selection	17	Give numbers of studies screened, assessed for eligibility, and included in the review, with reasons for exclusions at each stage, ideally with a flow diagram.	p.8-9, 13 and Figures 1 and 2
Study characteristics	18	For each study, present characteristics for which data were extracted (e.g., study size, PICOS, follow-up period) and provide the citations.	Table 1 and Supplementary Tables S2-S4
Risk of bias within studies	19	Present data on risk of bias of each study and, if available, any outcome level assessment (see item 12).	Supplementary Tables S5-S9
Results of individual studies	20	For all outcomes considered (benefits or harms), present, for each study: (a) simple summary data for each intervention group (b) effect estimates and confidence intervals, ideally with a forest plot.	p 9-11, p. 13-16, Table 2-3, and Figures 3 and 4
Synthesis of results	21	Present results of each meta-analysis done, including confidence intervals and measures of consistency.	p.14-16 and Table 3
Risk of bias across studies	22	Present results of any assessment of risk of bias across studies (see Item 15).	p.11-12 and Supplementary Table S5
Additional analysis	23	Give results of additional analyses, if done (e.g., sensitivity or subgroup analyses, meta-regression [see Item 16]).	p.16-17 and Table 4
**DISCUSSION**			
Summary of evidence	24	Summarize the main findings including the strength of evidence for each main outcome; consider their relevance to key groups (e.g., healthcare providers, users, and policy makers).	p.17-21
Limitations	25	Discuss limitations at study and outcome level (e.g., risk of bias), and at review-level (e.g., incomplete retrieval of identified research, reporting bias).	p. 22
Conclusions	26	Provide a general interpretation of the results in the context of other evidence, and implications for future research.	p.22-23
**FUNDING**			
Funding	27	Describe sources of funding for the systematic review and other support (e.g., supply of data); role of funders for the systematic review.	p.37

*Abbreviations: NA, not applicable; p, page.

Supplementary Figure S2. Data sources and search criteria for systematically reviewing hepatitis C virus (HCV) antibody incidence and prevalence in Egypt. Same data sources and search criteria were used for systematically reviewing HCV genotypes in Egypt.

PubMed (last searched: August 3, 2015)

("Hepatitis C"[Mesh] OR "Hepacivirus"[Mesh] OR "Hepatitis C, Chronic"[Mesh] OR "Hepatitis C Antibodies"[Mesh] OR "Hepatitis C Antigens"[Mesh] OR "HCV"[Text] OR "Hepatitis C"[Text]) AND ("Egypt" [Text] OR "Egyptian" [Text] OR "Egyptians" [Text])

Embase (last searched: August 3, 2015)

(exp hepatitis C/ or exp Hepatitis C virus/ or hepatitis C.mp. or HCV.mp. or hepacivirus.mp.) And (Egypt.mp. or Egyptian.mp. or Egyptians.mp.)

Abstract archives of the International AIDS Society conferences (last searched: November 11, 2015):

Hepatitis C and Egypt

Index Medicus for the Eastern Mediterranean Region (last searched: November 16, 2014)

Hepatitis AND C AND Egypt

Middle East and North Africa HIV/AIDS Epidemiology Synthesis Project database (last searched: November 24, 2015)

All country-level and international organizations' reports for Egypt available in the database

Supplementary Table S1. Lists of variables extracted from relevant reports with hepatitis C virus (HCV) antibody incidence and/or prevalence information or with HCV genotype information.

**List of extracted variables from relevant reports with:**	
HCV incidence and/or prevalence information	HCV genotype information
Author, year of publication	Author, year of publication
Full citation	Full citation
Year of data collection	Year of data collection
Publication type	Publication type
Country of origin	Country of origin
Country of survey (few studies were conducted among the Egyptian expatriate population)	Country of survey (few studies were conducted among the Egyptian expatriate population)
City	Study population
Study site	Sample size for HCV RNA testing
Study design	Sample size of HCV RNA positive individuals
Study sampling procedure	Prevalence of HCV genotypes
Study population	Prevalence of single and mixed HCV genotypes
Characteristics of study population (sex, age, nationality…)	Subtype information
Response rate	
Sample size	
HCV antibody incidence (and related follow-up time)	
HCV antibody prevalence	
Type of HCV ascertainment	
Type of assay used for HCV ascertainment	
Type of assay used for confirmatory testing	
HCV RNA prevalence	
Risk factors for HCV infection (identified as significant after controlling for confounders through multivariable regression analyses)	

Supplementary Figure S3. Description of quality assessment criteria and studies' risk of bias (ROB) appraisal.

The quality of HCV antibody incidence or prevalence measures identified through our review was determined by assessing:

1. The risk of bias (ROB) based on three quality domains
    a. Rigor of the sampling methodology
    b. Type of HCV ascertainment
    c. Response rate

2. The precision of the reported measures
    Studies were considered as having high precision if the number of HCV tested individuals was at least 100 participants. For an HCV prevalence of 1%, and a sample size of 100, the 95% CI is 0-5%; a reasonable precision for a prevalence measure.

Studies' ROB appraisal

1. Low ROB
    a. Probability-based sampling
    b. HCV ascertainment based on biological assays
    c. Response rate ≥80%

2. High ROB
    a. Non-probability sampling
    b. HCV ascertainment based on self-report
    c. Response rate <80%

3. Unclear ROB
    Studies with missing information for any of the domains were classified as having *unclear* ROB for that specific domain.

Note: HCV measures among individuals presenting voluntarily to facilities where routine blood screening is conducted, or retrieved from patients' medical records, were considered as having low ROB on specifically the response rate domain.

Supplementary Table S2. Studies reporting hepatitis C virus (HCV) antibody prevalence among the general population (populations at low risk) in Egypt.

First author, year of publication [citation]	Year(s) of data collection	City or governorate	Study site	Study design	Sampling	Population	Sample size	Anti-HCV prev	RNA prev among anti-HCV+	RNA prev among whole sample
**General population in Egypt (n=108)**										
El-Kamary, 2015 [2]	2012-13	Cairo	ANC	CS	Conv	Pregnant women	1,250	4.2%	57.7%	2.4%
Badr, 2015 [3]	2013-14	Cairo	Regional blood transfusion center	CS	Conv	Blood donors	33,921	1.8%	-	-
Jhaveri, 2015 [4]	2012-14	Cairo	ANC	Pros cohort	Conv	Pregnant women	2,514	3.9%	55.1%	2.1%
Abd Elrazek, 2014 [5]	2004-13	Rural/urban gov	Medical centers	Pros cohort	Conv	Patients attending medical center	6,660	15.3%	-	15.3%
Abdel Messih, 2014 [6]	2010-11	Cairo	Blood bank in a hospital	CS	Conv	Blood donors	17,118	3.8%	-	-
Edris, 2014 [7]	2011-12	Damietta	Community	CS	MsCS	Household survey residents	2,977	9.3%	-	-
Farghaly, 2014 [8]	-	Assiut	Hospital	CS	Conv	Healthy children	50	6.0%	66.7%	-
Hussein, 2014 [9]	2006-12	Cairo	Blood bank in a hospital	Ret cohort	Conv	Blood donors	308,762	4.3%	-	-
Khamis, 2014 [10]	-	Belal, Abo-elyosr, Elishaa, Soliman, Adam, Abd Elwahed, Abd Alrakeeb, Alemam Malek, Yousef	ANC	CS	Conv	Pregnant women	360	6.1%	45%	-
El-Shanshory, 2013 [11]	2010-11	Tanta	Hospital	CC	Conv	Healthy blood donors	3,756	17.5%	-	-
Badr, 2013 [12]	2012	Menoufia	Regional blood transfusion center	CS	Conv	Family replacement blood donors	2,847	8.1%	-	-
Badr, 2013 [12]	2012	Menoufia	Regional blood transfusion center	CS	Conv	Voluntary blood donors	5,145	4.9%	-	-
Hamed, 2012 [13]	2010-11	Assiut	Blood bank	CS	Conv	Blood donors	7,340	2.3%	-	-
Hussein, 2012 [14]	2006-11	-	Blood bank in a hospital	Ret cohort	Conv	Volunteer and family replacement blood donors	259,620	4.6%	-	-
Maha, 2012 [15]	2011	Menoufia	Regional Blood Transfusion Center	Ret cohort	Conv	Voluntary blood donors	10,598	4.8%	-	-
Maha, 2012 [15]	2011	Menoufia	Regional Blood Transfusion Center	Ret cohort	Conv	Family replacement blood donors	4,626	8.7%	-	-
Mansour, 2012 [16]	-	Mansoura	Hospital	CC	Conv	Healthy controls	100	0.0%	-	-
Schiefelbein, 2012 [17]	2007-09	Tanta and Gharbiyah gov	Hospital	CC	Conv	Healthy controls	148	49.3%	-	12.3%
Awadalla, 2011 [18]	-	Cairo	Blood bank in a hospital	CS	Conv	Blood donors	1,000	16.8%	-	-

Barakat, 2011 [19]	2005	Alexandria	National	CS	MsCS	School children	500	5.8%	75.9%	-
Teruya J, 2011 [20]	2011-unknown	Cairo	Blood bank	CS	Conv	Blood donors	3,425	1.6%	-	-
Wasfi, 2011 [21]	2007-08	Alexandria	Blood bank in a hospital	CS	Conv	Unpaid voluntary donors	3,420	3.5%	-	-
AbdulQawi, 2010 [22]	2003-08	Benha	Hospital	Pros cohort	Conv	Pregnant women	1,224	8.6%	79.0%	-
El-Karaksy, 2010 [23]	2006-07	Cairo	Hospital	CC	Conv	Healthy children controls	1,042	1.4%	33.3%	-
Khattab, 2010 [24]	2000-08	Minia	Central Laboratory	Ret cohort	Conv	Voluntary blood donors	211,772	9.0%	-	-
Mostafa, 2010 [25]	2001-06	Menoufia gov	Community	Pros cohort	MsCS	Village residents : >4 years	4,129	12.3%	-	-
Ashour, 2009 [26]	2006-08	Cairo, Alexandria, El Gharbia, El Dakahleya, El Behera, Ismailia, Minya, Sohag, Aswan	National blood transfusion center	CS	Conv	Voluntary non-remunerated blood donors	515,758	4.8%	-	-
Eita, 2009 [27]	2005-08	Dakahlia gov	blood bank	CS	Conv	Voluntary blood donors	73,431	4.6%	-	-
Eita, 2009 [27]	2005-08	Dakahlia gov	blood bank	CS	Conv	Family replacement blood donors	113,504	5.5%	-	-
Elkareh, 2009 [28]	2008	Menoufia gov	Regional blood transfusion center	Ret cohort	Conv	Family replacement blood donors	4,709	12.7%	-	-
Elkareh, 2009 [28]	2008	Menoufia gov	Regional blood transfusion center	Ret cohort	Conv	Voluntary blood donors	3,569	6.3%	-	-
Elkareh, 2009 [28]	2008	Menoufia gov	Blood bank in a hospital	Ret cohort	Conv	Family replacement blood donors	8,705	14.6%	-	-
Elkareh, 2009 [28]	2008	Menoufia gov	Blood bank in a hospital	Ret cohort	Conv	Voluntary blood donors	414	8.7%	-	-
Goldman, 2009 [29]	1999-04	Cairo	Hospital	CC	Conv	Healthy controls	786	37.4%	-	23.8%
Ismail, 2009 [30]	2000-07	Mansoura	Blood bank in a hospital	CS	Conv	Blood donors	55,922	12.0%	-	-
Rushdy, 2009 [31]	2006-07	Ismailia gov	Regional blood transfusion center	CS	Conv	Blood donors	9,150	2.7%		
Said, 2009 [32]		Cairo	Hospital	CC	Conv	Healthy children controls	50	0%	-	0%
Shebl, 2009 [33]	1997-01	Nile delta	ANC (village)	Pros cohort	Conv	Pregnant women ANC	1,863	15.7%	-	10.9%
Aguilar, 2008 [34]	-	Fakkous	Community	CS	Conv	Rural village healthy residents: males	78	51%	68%	-
Aguilar, 2008 [34]	-	Fakkous	Community	CS	Conv	Rural village healthy residents: females	81	42%	73%	-
Elmagd, 2008 [35]	1976-04	Mansoura, Dakahlia gov	Hospital	CC	Conv	Controls	316	49.1%	-	-
El-Zayadi, 2008 [36]	2005-05	Cairo	National blood transfusion center	CS	Conv	Healthy volunteer blood donors	760	5%	-	-

Eassa, 2007 [37]	2006-07	El-Ghar village, Zagazig	Community	CS	MsCS	Household members	304	10.9%	-	-
Eissa, 2007 [37]	2000-05	Cairo	Blood bank	CS	Conv	Blood donors: 1995-2000	46,747	6.9%	-	-
Eissa, 2007 [37]	2000-05	Cairo	Blood bank	CS	Conv	Blood donors: 2000-2005	99,757	4.0%	-	-
El Damaty, 2007 [38]	1999-00	Cairo	Blood bank	Ret. cohort	Conv	Blood donors	2,845	7.6%	-	-
El Damaty, 2007 [38]	1996-97	Cairo	Ministry of Health	CS	MsCS	Blood donors	861	15.2%	-	-
El-Raziky, 2007 [39]	2004-04	Cairo	Hospital	CC	Conv	Children	1,042	1.4%	33.3%	0.5%
Kandil, 2007 [40]	2004-06	Cairo	Hospital	CC	Conv	Children	100	38%	-	-
Agha, 2006 [41]	-	-	-	CS	Conv	Blood donors	2,400	8%	-	-
El-Gilany, 2006 [42]	2002-03	Mansoura, Dakahlia gov	Community	Ret cohort	Conv	University student voluntary blood donors	2,157	2.7%	-	-
Mohamed, 2006 [43]	2002-02	Menoufia	Community	CS	Conv	Village residents	2,425	18.5%	-	-
Mohamed, 2006 [43]	1997-97	Nile Delta and Assiut	Community	CS	Conv	Household survey members	4,631	4.8%	-	-
Stoszek, 2006 [44]	1997-03	Nile delta	ANC (village)	CS	Conv	Pregnant women	2,587	15.8%	-	10.8%
Arafa, 2005 [45]	2002-03	Menoufia	Community	CS	Conv	Village residents	4,020	11.8%	59.9%	-
Hashish, 2005 [46]	-	Alexandria	Central Laboratory	CS	Conv	Blood donors	95	23.2%	-	-
Sayed, 2005 [47]	-	El Shobak El Sharki village, Guiza gov	Community	CS	SRS	Village residents	84	14.3%	-	-
Ezzat, 2005 [48]	-	Cairo	Hospital	CC	Conv	Male Controls	63	30.2%	-	20.6%
Ezzat, 2005 [48]	-	Cairo	Hospital	CC	Conv	Male Controls	113	45.1%	-	35.4%
Cowgill, 2004 [49]	1999-03	Cairo	Hospital	CC	Conv	Controls	222	36.4%	65%	23.4%
El-Sadawy, 2004 [50]	-	Sharkia gov	Community	CS	MsCS	Urban and rural residents	1,422	25.8%	29.7%	7.7%
Tanaka,2004 [51]	1999-99	13 governorates (Central, North, East, and South)	Community	CS	Conv	Blood donors	3,608	8.8%	71%	-
El-Sherbini, 2003 [52]	1994-94	-	Community	CS	Conv	School children	294	5.8%	41%	-
Hadhoud, 2003 [53]	-	-	-	CS	Conv	Other general population	110	50%	-	-
Strickland, 2002 [54]	-	Shebin El Kom, Menoufia gov	Hospital	CC	Conv	Controls	212	46.7%	-	74.7%
Darwish, 2001 [55]	1994-94	Nile Delta	Community	CC	Conv	Semi-urban village residents aged 10 years and above	796	40%	-	-
Gad, 2001 [56]	1998-98	Ismailia, Suez, Port Said gov	Hospital	CC	Conv	Blood donor controls	50	20%	-	10.0%
Abdel-Aziz, 2000 [57]	1997-97	Qaluobyia gov, Aghour El-Soughra, Nile Delta	Community	CS	Random cluster sampling	Village residents	3,999	24.3%	65.5%	-
Kassem, 2000 [58]	1996-96	Alexandria	Hospital	CS	Conv	Pregnant women	100	19%	73.7%	14.0%
Nafeh, 2000 [59]	-	Assiut	Community	CS	Conv	Village household residents >5 years old	6,031	8.7%	63.0%	-
Yates, 1999 [60]	-	Cairo	Hospital	CC	Conv	Healthy controls	466	15.7%	-	-

Agha, 1998 [61]	1996-97	Mansoura, Dakahlia gov	Hospital	CS	Conv	Pregnant women	767	13.7%	26.9%	-
El-Zayadi, 1998 [62]	-	Cairo	Hospital	CS	Conv	Blood donors	75	16%	-	-
Farghaly, 1998 [63]	-	-	-	CS	Conv	Healthy controls	100	8%	-	-
Arthur, 1997 [64]	1993-93	Mixed gov	Blood bank in a hospital	CS	Proportional sampling	Blood donors	2,644	24.8%	-	-
El-Sayed, 1997 [65]	1993-94	East of Bitter lakes, Sinai peninsula	Community	CS	SRS	Permanent settlers	506	10.3%	-	-
El-Zayadi, 1997 [66]	-	-	-	CC	Conv	Blood donors	320	16.3%	-	-
El-Zayadi, 1997 [66]	-	-	-	CC	Conv	Blood donors	180	9.4%	-	-
Al Omar, 1996 [67]	1993-94	Assiut	Hospital	CS	Conv	Blood donors	143	34%	-	-
Attia, 1996 [68]		Cairo	Hospital	CS	Conv	Paid and unpaid blood donors	156	21.8%	-	-
El-Sayed, 1996 [69]	1994-94	Sinai gov	Community	CS	Conv	Workers supporting the tourist industry	740	14.3%	-	-
Mohamed, 1996 [70]	-	Upper and lower Egypt, seashore and Cairo gov	Ministry of Health	CS	Conv	Egyptians applying work abroad	5,071	31.5%	-	-
Bassily, 1995 [71]	-	Cairo	Transfusion center in a hospital	CC	Conv	Blood donors	188	26.6%	-	-
Darwish, 1995 [72]	-	-	Community	CS	Conv	Healthy villagers and non-professional blood donors	188	21.8%	76%	-
El Gohary, 1995 [73]	1990-92	Suez canal area urban (Ismailia, Suez, Port Said governorates)	Blood bank in a hospital	CS	Conv	Healthy blood donors	1,187	14.5%	-	-
El Gohary, 1995 [73]	1990-92	Suez canal area rural (Ismailia, Suez, Port Said governorates)	Community	CS	Conv	Blood donors	271	14.4%	-	-
El Gohary, 1995 [73]	1990-92	North Sinai rural (Katia village)	Community	CS	Conv	Blood donors	148	15.5%	-	-
El-Nanawy, 1995 [74]	-	Alexandria	Hospital	CC	Conv	Healthy children controls	110	11.8%	-	-
Quinti, 1995 [75]	1992-94	Alexandria	-	CS	Conv	Blood donors	283	20.8%	-	-
Quinti, 1995 [75]	1995	Alexandria	-	CS	Conv	Fire brigade personnel	541	39%	-	-
Abdel-Wahab, 1994 [76]	1992-92	Menoufia gov	Community	CS	Conv	Primary school children, 8-12 year old male students	190	12.1%	-	-
Abdel-Wahab, 1994 [76]	1992-92	Menoufia gov	Community	CS	Conv	Rural community inhabitants: >5 years old	270	18.1%	-	-
Abdel-Wahab, 1994 [76]	1992-92	Nile delta	-	CS	Conv	Army recruits	300	22.1%	-	-
Abdel-Wahab, 1994 [76]	1992-92	Menoufia gov	Community	CS	Conv	Rural community inhabitants: 5-10 years old	58	0%	-	-
Abdel-Wahab, 1994 [76]	1992-92	Menoufia gov	Community	CS	Conv	Rural community inhabitants: 10-20 years old	77	14.3%	-	-
Farghaly, 1994 [77]	-	-	-	CC	Conv	Military conscripts	726	30.4%	-	-

Kamel, 1994 [78]	1992-92	Saada, Kafr El Sheikh	Community	CS	Conv	Village inhabitants	1,259	15.9%	-	-
Nooman, 1993 [79]	-	Sinai gov	Community	CS	Conv	Rural village residents	1,000	0.01%	-	-
Nooman, 1993 [79]	-	Ismailia	Community	CS	Conv	Rural village residents	2,000	0.02%	-	-
Hassan, 1993 [80]	-	-	-	CS	Conv	Pregnant women	1,536	4.3%	-	-
Khalifa, 1993 [81]	1990-91	Cairo	Hospital	CS	Conv	Healthy outpatients	84	0%	-	-
Darwish, 1992 [82]	-	-	-	CS	Conv	Non-professional blood donors	90	14.4%	-	-
Zakaria, 2005 [83]	1999-02	Giza, Cairo	Community	CS	Conv	Village residents	2,305	20.9%	-	-
Hagag, 1998 [84]	-	Sharkia gov	blood bank	CS	SRS	Volunteer blood donors	457	24.9%	-	-
Khedr, 1995 [85]	-	Ismailia, Suez, Port Said gov	Hospital	CC	Conv	Healthy individuals controls	91	18.7%	-	-
Helmy, 1995 [86]	-	-	-	CC	Conv	Control group	110	8.1%	-	-
El-Zanaty, 2008 [87]	2008-08	National	National	CS	MsCS	Household survey members: 15-59 years old	11,126	14.7%	-	9.8%
Ministry of Health and Population, 2015 [88]	2014-15	National	National	CS	MsCS	Household survey members:1-59 years old	26,047	6.3%	-	4.4%
**Egyptian expatriate general population (n=9)**										
Chehadeh, 2011 [89]	-	Kuwait, Jabriya & Kaifan	Hospital	CC	Conv	Egyptian residents of Kuwait-outpatient controls	132	3.0%	75%	2.0%
Derbala, 2014 [90]	2008-10	Qatar	Qatar health centers and the Egyptian embassy	CS	Conv	Male Egyptian residents of Qatar	2,335	13.5%	100%	13.5%
Perumalswami, 2014 [91]	2009-11	New Jersey & New York	Community	CS	Conv	Egyptian residents of USA	192	15.6%	-	-
Zuure, 2013 [92]	2009-10	Greater Amsterdam area	Community	CS	Targeted sampling	First-generation Egyptian migrants	465	2.4%	90.9%	-
Mehdi, 2000 [93]	1995-97	Buraidah, KSA	Hospital	CS	Conv	Blood donors	1,609	16%	-	-
Fakeeh, 1999 [94]	1998-98	Jeddah, KSA	Hospital	CS	SRS	Outpatients (Egyptians in KSA)	284	22.5%	-	-
Kumar, 1997 [95]	1994-96	Al Ain, UAE	Hospital	CS	Conv	Healthy asymptomatic parturient women in the third trimester of pregnancy (Egyptians in UAE)	499	13%	31%	-
Ahmad, 1995 [96]	-	Makkah, KSA	Hospital	CS	Conv	Voluntary blood donors (Egyptians in KSA)	114	27.2%	-	-
Al-Knawy, 1995 [97]	-	Southern part, KSA	Blood bank in a hospital	Ret cohort	Conv	Unpaid male volunteer blood donors (Egyptians in KSA)	204	21.1%	-	-

[*]The table reports only studies whose sample size is greater or equal to 50 participants. For space considerations, the table shows the overall HCV measure of each study rather than stratifications within population subgroups.

[**]The decimal places of the prevalence figures are as reported in the original report, but prevalence figures with more than one decimal were rounded to one decimal place, with the exception of those below 0.1%.

[†]Abbreviations: ANC, antenatal clinic; CC, case-control; Conv, convenience; CS, cross-sectional; Gov, governorate; Prev, prevalence; Ret, retrospective; Pros, prospective; SRS, simple random sampling; MsCS, multi-stage cluster sampling; USA, United States of America; KSA, Kingdom of Saudi Arabia; UAE, United Arab Emirates.

Supplementary Table S3. Studies reporting hepatitis C virus (HCV) antibody prevalence among populations at high risk in Egypt.

First author, year of publication [citation]	Year(s) of data collection	City or governorate	Study site	Study design	Sampling	Population	Sample size	Anti-HCV prev	RNA prev among anti-HCV+	RNA prev among whole sample
**Populations at high risk (n=35)**										
Adly, 2015 [98]	-	Cairo	Hospital	CS	Conv	Thalassemia patients	176	42%	-	-
Helaly, 2015 [99]	2012	Alexandria	Hemodialysis unit	CS	Conv	Hemodialysis patients	100	34%	-	-
Salama, 2015 [100]	-	Cairo	Hematology clinic	CS	Conv	Thalassemia patients	80	50%	55%	-
Hussein, 2014 [101]	2007-08	Cairo	Hematology clinic	CS	Conv	Thalassemia patients	200	24%	100%	24%
Zahran, 2014 [102]	-	Menoufia	Hemodialysis unit	Ret cohort	Conv	Hemodialysis patients	514	49.6%	-	-
El Sayed Zaki, 2013 [103]	-	-	-	CC	Conv	Children with thalassemia	100	15.0%	-	-
Elalfy, 2013 [104]	2009-10	Cairo	Hospital	CS	Conv	Young thalassemia patients	51	82%	-	-
El-Shanshory, 2013 [11]	2010-11	Tanta	Hospital	CC	Conv	Children with thalassemia	125	76.0%	-	40.0%
Said, 2013 [105]	-	-	-	CS	Conv	Thalassemia patients	137	34.4%	-	34.4%
Tantawy, 2013 [106]	-	Cairo	Hospital	CS	Conv	Children and young adults with thalassemia	84	82%	-	-
Kamal, 2013 [107]	2011	Cairo	Hospital	CS	Conv	Hemodialysis patients	170	60.6%	-	-
Abdelwahab, 2012 [108]	-	Cairo & Agouza	Hematology clinic	CS	Conv	Hemophilic children	100	40.0%	47.5%	-
El-Faramawy, 2012 [109]	-	Qena	Blood bank in a hospital	CS	Conv	Multi-transfused children	100	45.0%	-	-
Khodir, 2012 [110]	2011	Al-Gharbiyah gov	Hemodialysis unit	Pros cohort	Conv	Hemodialysis patients	2,351	35.0%	-	-
Mansour, 2012 [16]	-	Mansoura	Hospital	CC	Conv	Thalassemia patients	200	19.5%	-	-
Omar, 2011 [111]	-	Cairo	Hospital	CS	Conv	Thalassemia patients	174	51.7%	74.3%	-
El-Waseef, 2010 [112]	2003	Cairo	National Heart Institute	CC	Conv	Multi-transfused children	80	31.3%	-	-
Ibrahim, 2010 [113]	2007	Cairo	Hospital	Ret cohort	Conv	Hemodialysis patients	100	70.0%	-	-
Attia, 2010 [114]	2008-09	Cairo	Hospital	CC	Conv	Uremic adults and children undergoing hemodialysis	206	46.1%	-	-
Said, 2009 [32]	-	Cairo	Hospital	CC	Conv	Multi-transfused children	51	17.6%	100%	23.5%
Khalifa, 2004 [115]	2000-03	Cairo	Hospital	CC	Conv	Children with thalassemia	56	69.6%	-	-
Shatat, 2000 [116]	1999-99	-	-	CS	Conv	Hemodialysis patients	65	78.5%	-	-
Abdel Hady, 1998 [117]	-	-	-	CS	Conv	Hemodialysis patients	96	27.1%	-	-
El Gohary, 1995 [73]	1990-92	Ismailia, Suez, Port Said gov	Hospital	CS	Conv	Hemodialysis patients	108	70.4%	-	-
El-Ghazzawi, 1995 [118]	-	Alexandria	-	CC	Conv	Intravenous drug addicts	100	63%	-	-
Gohar, 1995 [119]	-		-	CC	Conv	Hemodialysis patients	64	87.5%	-	-
Abdel-Wahab, 1994 [76]	1992-92	Cairo	Hospital	CS	Conv	Multi-transfused children	71	54.9%	-	-
Abdel-Wahab, 1994 [76]	1992-92	Cairo	Hospital	CS	Conv	Hemodialysis patients	78	46.2%	-	-
Hassan, 1993 [120]	1991-93			CS	Conv	Renal dialysis patients	105	67%	-	-

Khalifa, 1993 [81]	1990-91	Cairo	Hospital	CS	Conv	Thalassemia patients	84	55%	-	-
Goher, 1998 [121]	-	Cairo	Hemodialysis center	CS	Conv	Hemodialysis patients	335	71.9%	-	-
Saddik, 1997 [122]	-	Al-Qalyubiya, Cairo, Dakahlia gov	Hospital	CC	Conv	Hemodialysis patients	50	72%	-	-
El Alfy, 1997 [104]	-	Cairo	Hospital	CC	Conv	Thalassemia patients	135	8.9%	-	-
Helmy, 1995 [86]	-	-	-	CC	Conv	Renal dialysis patients	250	68%	-	-
Helmy, 1995 [86]	-	-	-	CC	Conv	Renal dialysis patients	100	98%	-	-

*The table reports only studies whose sample size is greater or equal to 50 participants. For space considerations, the table shows the overall HCV measure of each study rather than stratifications within population subgroups.

**The decimal places of the prevalence figures are as reported in the original report, but prevalence figures with more than one decimal were rounded to one decimal place, with the exception of those below 0.1%.

†Abbreviations: Conv, convenience; CC, Case-control; CS, cross-sectional; Gov, governorate; Prev, prevalence; Ret, retrospective; Pros, prospective.

Supplementary Table S4. Studies reporting hepatitis C virus (HCV) antibody prevalence among populations at intermediate risk, populations with liver-related conditions, special clinical populations, and mixed populations in Egypt.

First author, year of publication [citation]	Year(s) of data collection	City or governorate	Study site	Study design	Sampling	Population	Sample size	Anti-HCV prev	RNA prev- among anti-HCV+	RNA prev- among whole sample
**Populations at intermediate risk (n=31)**										
El-bendary, 2015 [123]	-	-	Community	CS	Conv	Household contacts of index patients	321	13.7%	-	-
Okasha, 2015 [124]	2008	Cairo	Hospital	CS	Conv	Health care workers	1,770	8.0%	-	-
Farghaly, 2014 [8]	-	Assiut	Hospital	CS	CC	Diabetic children	150	12.0%	66.7%	-
Elwan, 2013 [125]	-	Nile Delta	-	CS	Conv	Household contacts of index patients	481	12.7%	-	-
Mohamed, 2013 [126]	-	Minia	Prison	CS	SRS	Prisoners	500	15.8%	77.2%	-
Said, 2013 [105]	-	-	-	CS	Conv	Household contacts of index patients	73	19.2%	-	-
Said, 2013 [105]	-	-	-	CS	Conv	Household contacts of index patients	82	4.9%	-	-
Munier, 2013 [127]	2008-10	Cairo	Hospital	CS	Conv	Health care workers	597	7.2%	51.2%	-
Tantawy, 2013 [128]	2010	Cairo	Hospital	Pros cohort	Conv	Inpatients (neutropenia children)	200	0%	-	-
Abdelwahab S, 2012 [129]	2008-10	Menoufia	National Liver Institute	CS	Conv	Health care workers	842	16.6%	72.1%	-
Morad, 2011 [130]	-	-	Hospital	CS	Conv	Spouses of index patients	200	35.5%	-	-
El-Karaksy, 2010 [23]	2006-07	Cairo	Hospital	CC	Conv	Diabetic children	692	3.6%	40.0%	-
Shalaby, 2010 [131]	2007	Tanta & Mahalla El-Koubra	Community	CS	Block sampling technique and MsRS	Barbers and their clients'	616	12.5%	72.7%	9.1%
Elmagd, 2008 [35]	2008	Mansoura, Dakahlya gov	Hospital	CC	Conv	Diabetic patients	286	60.3%	-	-
Attallah, 2004 [132]	2004	Cairo	Hospital	CC	Conv	Hospitalized patients	73	90%	-	-
Madwar, 1999 [133]	1999	-	-	CS	Conv	Spouses of index patients	200	14%	100%	-
Madwar, 1999 [133]	1999	-	-	CS	Conv	Household contacts of index patients	355	0.0%	-	-
Ali, 1998 [134]	1998	Cairo	Hospital	CS	Conv	Patients attending STD clinic	95	8.4%	-	-
Farghaly, 1998 [63]	1998	-	-	CS	Conv	Patients with periodontal disease	100	13%	-	-
El-Zayadi, 1997 [135]	1997	Cairo	Hospital	CS	Conv	Household contacts of index patients	265	5.7%	-	1.1%
El Gohary, 1995 [73]	1995	Ismailia, Suez, Port Said gov	Hospital	CS	Conv	Health care workers	78	7.7%	-	-
Hindy, 1995 [136]	1995	Cairo	Hospital	CC	Conv	Dentists	105	6.7%	-	-
Quinti, 1995 [75]	1995	Alexandria	-	CS	Conv	Prisoners	124	31.4%	-	-
Hassan, 1993 [120]	1993	Unspecified	-	CS	Conv	Patients attending STD clinic	83	10%	-	-
Khalifa, 1993 [81]	1993	Cairo	Hospital	CS	Conv	Hospitalized population	84	0%	-	-

Hassane, 1998 [137]	1998	Shebin El Kom, Menoufia gov	Community	CS	Conv	Household contacts of index patients	96	17.7%	76.9%	7.3%
Hassane, 1998 [137]	1998	Shebin El Kom, Menoufia gov	Community	CS	Conv	Household contacts of index patients	99	9.1%	33.3%	3.0%
Hassane, 1998 [137]	1998	Shebin El Kom, Menoufia gov	Community	CS	Conv	Household contacts of index patients	86	27.9%	50%	14.0%
Hassane, 1998 [137]	1998	Shebin El Kom, Menoufia gov	Community	CS	Conv	Household contacts of index patients	81	13.6%	9.1%	1.2%
Khedr, 1995 [85]	1995	Ismailia, Suez, Port Said gov	Hospital	CC	Conv	Diabetic patients	109	26.6%	-	-
Ismail,1994 [138]	1994	Minia	Hospital	CC	Conv	Health care workers	50	0%	-	-
**Egyptian expatriate population at intermediate risk (n=1)**										
Chehadeh, 2011 [89]	-	Kuwait, Jabriya & Kaifan	Hospital	CC	Conv	Egyptian residents of Kuwait with type II diabetes mellitus	113	18.0%	80%	14.0%
**Populations with liver-related conditions (n=45)**										
Gad, 2015 [139]	2003-13	Menoufia	Transfusion center in a hospital	Ret cohort	Conv	Liver transplant patients	167	16.8%	-	-
El Azm, 2013 [140]	2009-12	Tanta	Hospital	Ret cohort	Conv	Hepatocellular carcinoma patients	281	66.2%	-	-
Shaker, 2013[141]	2009-11	Cairo	Outpatient clinic of hepatocellular carcinoma unit	Ret cohort	Conv	Hepatocellular carcinoma patients	1,313	91.3%	-	-
Badawy, 2012 [142]	-	-	Hospital	CS	Conv	Acute viral hepatitis patients	99	8.0%	-	-
EL-Meteini, 2012 [143]	2002-11	-	Single center experience	CS	Conv	Hepatocellular carcinoma patients	146	95.2%	-	-
Schiefelbein, 2012 [17]	2007-09	Tanta & Gharbiyah gov	Hospital	CC	Conv	Liver cancer patients	148	89.2%	-	81.1%
Taha, 2012 [144]	2007	-	Outpatient clinic	CS	Conv	Hepatocellular carcinoma patients	1,643	70.0%	-	-
El Sayed Zaki, 2011 [145]	2009-10	Mansoura	Hospital	CS	Conv	Acute on chronic liver failure patients	100	100.0%	-	30.0%
Eldin, 2010 [146]	2007-08	Assiut	Hospital	CS	Conv	Acute hepatitis patients	235	4.3%	-	-
Talaat, 2010 [147]	2001-04	Alexandria, Mahalla, Abassia, Qena, Aswan	Hospital	CS	Conv	Suspected acute viral hepatitis patients	4,189	29.8%	-	-
Goldman, 2009 [29]	1999-04	Cairo	Hospital	CC	Conv	Non-Hodgkin's Lymphoma patients	296	47.0%	-	38.9%
Youssef, 2009 [148]	-	Ismailia	Hospital	CS	Conv	Patients with liver complaints	214	72.9%	57.7%	-
Abdel-Wahab, 2008 [149]	2005-06	Mansoura, Dakahlia gov	Hospital	CC	Conv	Hepatocellular carcinoma patients	80	70%	-	-

Abdel Wahab, 2007 [150]	1992-05	Mansoura, Dakahlia gov	Hospital	Ret cohort	Conv	Hepatocellular carcinoma patients	1,012	79.6%	-	-
Mikhail, 2007 [151]	2000-04	Shebin El Kom, Menoufia gov	National	Pros cohort	Conv	Chronic liver disease patients undergoing endoscopy	859	71%	-	-
Zakaria, 2007 [152]	2001-02	Cairo	Hospital	CS	Conv	Acute viral hepatitis patients	200	13%	-	-
El Gaafary, 2005 [153]	2002	Cairo	Hospital	CS	Conv	Acute viral hepatitis patients	309	11%	-	14.9%
El-Zayadi, 2005 [154]	1993-02	Cairo	Cairo Liver Center	CS	Conv	Chronic liver disease patients	22,450	72.3%	-	-
Ezzat, 2005 [48]	-	Cairo	National	CC	Conv	Hepatocellular carcinoma patients	63	87.3%	-	79.4%
Ezzat, 2005 [48]	-	Cairo	National	CC	Conv	Hepatocellular carcinoma patients	113	90.3%	-	86.6%
Cowgill, 2004 [49]	1999-03	Cairo	Hospital	CC	Conv	Non-Hodgkin's Lymphoma patients	220	48.2%	88.7%	42.7%
Takagi, 2003 [155]	2002	Alexandria	Hospital	CS	Conv	Suspected acute viral hepatitis patients	57	82.5%	-	-
Zaki, 2003 [156]	-	Alexandria	Community	CS	Conv	Patients with clinically detected organomegally (hepatomegally and/or splenomegally)	65	33.8%	-	-
Blanton, 2002 [157]	1999-00	Kalama, semiurban village in Nile Delta	Community	CS	Conv	Hepatic Schistosomiasis	141	39.7%	-	-
Strickland, 2002 [54]	-	Shebin El Kom, Menoufia gov	Hospital	CC	Conv	Chronic liver disease patients	237	58.2%	69.6%	-
Gad, 2001 [56]	1998	Ismailia, Suez, Port Said gov	Hospital	CC	Conv	Chronic liver disease patients	88	76%	-	59.0%
Rahman El-Zayadi, 2001 [158]	1992-95	Cairo	Cairo Liver Center	Ret cohort	Conv	Patients with liver complaints	6,850	71.1%	-	-
Abdel-Wahab, 2000 [159]	1994-99	Mansoura, Dakahlia gov	Hospital	CS	Conv	Hepatocellular carcinoma patients	385	61%	-	-
Yates, 1999 [60]	-	Cairo	Hospital	CC	Conv	Hepatocellular carcinoma patients	131	76%	-	-
Khalifa, 1999 [160]	-	-	-	CC	Conv	Patients with liver cirrhosis	61	56%	-	-
Angelico M, 1997 [161]	1993-95	Alexandria	Hospital	CS	Conv	Chronic liver disease patients	141	67.4%	55.0%	-
Darwish, 1997 [162]	-	-	-	CS	Conv	Hepatocellular carcinoma and patients with liver cirrhosis	94	75.5%	-	-
El-Zayadi, 1997 [66]	-	-	-	CC	Conv	Chronic liver disease patients	612	62.1%	-	-
El-Zayadi, 1997 [66]	-	-	-	CC	Conv	Chronic liver disease patients	316	38.6%	-	-
Madwar, 1997 [163]	-	-	-	Pros cohort	Conv	Suspected acute viral hepatitis patients	120	43.2%	-	-
Quinti, 1997 [164]	-	Cairo	Hospital	Pros cohort	Conv	Acute viral hepatitis patients	110	27.3%	87.0%	-
Gomatos, 1996 [165]	1993-94	Cairo	Hospital	CS	Conv	Acute viral hepatitis patients	143	8.4%	-	-
Waked, 1995 [166]	1992	Menoufia	Hospital	CS	Conv	Chronic liver disease patients	1,023	73.5%	-	-
Abdel-Wahab, 1994 [76]	1992	Cairo	Hospital	CS	Conv	Hepatic schistosomiasis	55	16.4%	-	-
Abdel-Wahab, 1994 [76]	1992	Cairo	Hospital	CS	Conv	Chronic liver disease patients	354	47.2%	-	-
El-Gohary, 1994 [167]	-	Ismailia, Suez, Port Said gov	Hospital	CS	Conv	Acute viral hepatitis patients	140	19.2%	-	-

Darwish, 1993 [168]	-	Cairo	Hospital	CS	Conv	Hepatocellular carcinoma patients	70	30%	-	-
Hassan, 1993 [120]	1991-93	-	-	CS	Conv	Acute viral hepatitis patients	207	29%	-	-
Mokhtar, 1996 [169]	-	Giza, Cairo	Hospital	CC	Conv	Acute viral hepatitis patients	60	6.7%	-	-
Salem, 1992 [170]	-	-	Hospital	CS	Conv	Acute viral hepatitis patients	70	17.1%	-	-
**Special clinical populations (n=27)**										
Mostafa, 2015 [171]	2012-14	Cairo	Oral and dental outpatient clinic	Ret cohort	Conv	Lichen planus patients	64	17.9%	-	-
Gabr, 2014 [172]	2011	Tanta	Hospital	CS	Conv	Patients undergoing endoscopy	103	96.1%	-	-
Mousa, 2014 [173]	-	Cairo	Hematology unit	CS	Conv	Patients undergoing bone marrow examination	100	42.0%	-	-
Taha, 2014 [174]	2011-13	Assiut	Dermatology and Venereology outpatient clinic	CS	Conv	Patients with psoriasis vulgaris	153	13.1%	-	-
Abd-El-Moneim, 2013 [175]	2009-unknown	Alexandria	Hospital	CS	Conv	Breast cancer patients on chemotherapy	102	51.0%	-	-
Azim, 2013 [176]	2000-08	Cairo	Oncology center	Ret cohort	Conv	Diffuse large B cell lymphoma patients	230	7.4%	-	-
El-Khalawany, 2013 [177]	2002-12	Dumyat, Al-Zahraa and Tanta	Hospital	Pros cohort	Conv	Adult patients with Tinea capitis	58	34.4%	-	-
Mattar, 2013 [178]	2003-13	Cairo	Hematology center	CS	Conv	Polycythemia vera patients	90	6.7%	-	-
Aboueisha, 2013 [179]	-	Cairo	Hospital	Ret cohort	Conv	Living liver donors	108	7.4%	-	-
El Garf, 2012 [180]	2009	Cairo	Hospital	CS	Conv	Rheumatoid arthritis patients	157	18.5%	71.4%	-
Youssef, 2012 [181]	2010-11	Cairo	Hospital	CC	Conv	Non-Hodgkin Lymphoma, patients with Hodgkin disease, and patients with chronic lymphocytic leukemia	50	26.0%	-	26.0%
El Husseiny, 2011 [182]	2007-10	Cairo	Hematology unit	Ret cohort	Conv	Myelodysplastic syndrome patients	69	13.0%	-	-
El-Sabah, 2011 [183]	-	Cairo & Gharbiya gov.	Community	CS	Conv	Patients on oral/parenteral anti-schistosomiasis	102	45.1%	-	-
Mahmoud, 2011 [184]	2009-10	Cairo	Hospital	CS	Conv	Rheumatoid arthritis patients	110	20.0%	63.6%	-
Abou-Zeid, 2011 [185]	-	Alexandria	Hospital	CC	Conv	Glomerulonephritis patients	78	59.0%	-	-
Sabry, 2010 [186]	2000-03	Mansoura	Hospital	Ret cohort	Conv	Renal transplant patients	273	62.0%	-	-
Abdel Wahab, 2007 [150]	1995-04	Dakahlia, Gharbia, Kafer El-Sheikh, Sharkia, Damietta, Port Said, Upper Egypt, Menfia, Ismaelia	Hospital	CS	Conv	Hilar cholangiocarcinoma	440	54%	-	-
Sharaf-Eldeen, 2007 [187]		Damietta and Cairo	Hospital	CS	Conv	Lichen planus patients	100	43%	76.8%	-

Sabry, 2005 [188]	-	Mansoura, Dakahlia gov	Clinic	CS	Conv	Glomerulonephritis patients/glomerulopathy	233	38%	55.6%	-
Demian, 2004 [189]	-	Mansoura, Dakahlia gov	Hospital	CS	Conv	Patients undergoing urological procedures	667	45.1%	-	-
Sabry, 2002 [190]	1998-99	Mansoura, Dakahlia gov	Hospital	CS	Conv	Glomerulonephritis patients/glomerulopathy	303	38%	-	-
Meir, 2001 [191]	-	-	Hospital	CS	Conv	Cancer patients on chemotherapy	54	19%	-	-
Yates, 1999 [60]	-	Cairo	Hospital	CC	Conv	Patients with bladder cancer	247	47%	-	-
Attia, 1996 [68]	-	Cairo	Hospital	CS	Conv	Patients with various cancer	429	53.4%	-	-
Hassaballa, 1996 [192]	1989-94	-	-	CC	Conv	Kidney transplant patients	54	79.6%	-	-
Mostafa, 2003 [193]	2000-01	Cairo	Hospital	CS	Conv	Cancer patients on chemotherapy	99	13.1%	38.5%	-
Mostafa, 2003 [193]	2000-01	Cairo	Hospital	CS	Conv	Cancer patients on chemotherapy	111	39.6%	47.7%	-
**Mixed populations (n=3)**										
El-Mougy, 2014 [194]	-	-	-	Pros cohort	Conv	Hepatocellular carcinoma patients, Cirrhosis patients & healthy volunteers	58	63.8%	-	-
Kalil, 2010 [195]	2004-05	Assiut	Hospital	CC	Conv	Acute hepatitis, multi-transfused, non-hepatitis and non-multi transfused children	465	26.0%	72.2%	-
Hammad, 2009 [196]	2008	Mansoura	Hospital	CC	Conv	Chronic renal failure children (Hemodialysis and pre-dialysis)	100	52.0%	-	58.0%

[*]The table reports only studies whose sample size is greater or equal to 50 participants. For space considerations, the table shows the overall HCV measure for each study rather than stratifications within population subgroups.

[**]The decimal places of the prevalence figures are as reported in the original report, but prevalence figures with more than one decimal places were rounded to one decimal place, with the exception of those below 0.1%.

[†]Abbreviations: STD, sexually transmitted diseases; Conv, convenience, CS, cross-sectional; CC, case-control; Gov, governorate; Prev, prevalence; MsRS, multi-stage random sampling; SRS, simple random sampling.

Supplementary Table S5. Summary of precision and risk of bias (ROB) assessment for hepatitis C virus (HCV) antibody incidence and prevalence measures extracted from eligible reports in Egypt.

**Quality assessment**	**HCV antibody incidence measures**		**Anti-HCV prevalence measures**	
	**n**	**%**	**N**	**%**
**Precision of estimates**				
High precision	11	44.0	198	76.4
Low precision	14	56.0	61	23.6
**Risk of bias quality domains**				
**Sampling methodology**				
Low risk of bias	0	0	14	5.4
High risk of bias	24	96.0	245	94.6
Unclear*	1	4.0	0	0
**HCV ascertainment**				
Low risk of bias	23	92.0	228	88.0
High risk of bias	0	0	0	0
Unclear*	2	8.0	31	12.0
**Response rate**				
Low risk of bias	2	8.0	79	30.5
High risk of bias	7	28.0	13	5.0
Unclear*	16	64.0	167	64.5
**Total number of studies where risk of bias assessment was possible**	**25**	**100**	**259**	**100**
**Total number of studies**	**25**	**100**	**259**	**100**
**Summary of risk of bias assessment for HCV antibody incidence and prevalence measures**				
	**n**	**%**		
**Low risk of bias**				
In at least one quality domain	250	88.0		
In at least two quality domains	138	48.6		
In all three quality domains	7	2.5		
**High risk of bias**				
In at least one quality domain	269	94.7		
In at least two quality domains	18	6.3		
In all three quality domains	0	0		
**Total number of studies where risk of bias assessment was possible**	**284**	**100**		
**Total number of studies**	**284**	**100**		

*Studies with missing information for any of the domains were classified as having unclear ROB for that specific domain.

Supplementary Table S6. Precision and risk of bias (ROB) assessment for individual hepatitis C virus (HCV) antibody incidence measures in Egypt.

First author, year of publication [citation]	Year(s) of data collection	Population	Sample size	HCV sero-conversion risk (relative to total sample size)	HCV incidence rate (per 1000 persons years)	Precision	Sampling	HCV ascertainment	Response rate
**General population**									
Mohamed, 2005 [197]	1997-2000	General population- household members surveyed in Aghour el Soughra village in Nile Delta	2,463	-	6.8	High	High ROB	Low ROB	High ROB
Mohamed, 2005 [197]	1997-2000	General population- household members surveyed in Sallam village in Upper Egypt	4,275	-	0.8	High	High ROB	Low ROB	High ROB
Mostafa, 2010 [25]	2001-2006	General population- household members surveyed in 3 villages in Menoufia governorate in Nile Delta	3,184	-	2.4	High	High ROB	Low ROB	Low ROB
Mikhail, 2007 [151]	2000-2004	Control subjects from a village in the Nile Delta	149	-	10.2	High	High ROB	Low ROB	High ROB
Saleh, 2008 [198]	1997-2006	Pregnant women surveyed in 3 villages in Menoufia governorate in Nile Delta	2,171	-	5.2	High	High ROB	Low ROB	Unclear
**Populations at high risk**									
El-Sherif, 2012 [199]	-	Hemodialysis patients	14	21.4	-	Low	High ROB	Low ROB	Unclear
Goher, 1998 [121]	-	Hemodialysis patients on non-reused dialyzers	37	21.6	-	Low	High ROB	Low ROB	Unclear
Goher, 1998 [121]	-	Hemodialysis patients on reused dialyzers	53	20.8	-	Low	High ROB	Low ROB	Unclear
Khodir, 2012 [110]	2011	Hemodialysis patients	1,527	11.0	-	High	High ROB	Low ROB	Unclear
Soliman, 2013 [200]	2008-2010	Hemodialysis patients following strict isolation program	27	14.8	-	Low	High ROB	Low ROB	Unclear
Soliman, 2013 [200]	2008-2010	Hemodialysis patients not following strict isolation program	56	42.9	-	Low	High ROB	Low ROB	Unclear
Zahran, 2014 [102]	-	Hemodialysis patients	303	14.5	-	Low	High ROB	Unclear	Unclear
**Populations at intermediate**									
Abdelwahab, 2013 [201]	2008-2011	Health care workers	651	0.3	2.0	High	High ROB	Low ROB	Low ROB
Munier, 2013 [127]	2008-2010	Health care workers	73	0	-	Low	High ROB	Low ROB	Unclear
Okasha, 2015 [124]	2008	Health care workers	402	-	7.3	High	High ROB	Low ROB	High ROB
Saleh, 2010 [202]	2000-2006	Children of HCV infected mothers	2,852	0.5	2.7	High	Unclear	Low ROB	High ROB
**Populations with liver-related conditions**									
Meky, 2006 [203]	2002-2005	Small sub-sample of patients with liver disease biomarkers in a community study	6	33.3	-	Low	High ROB	Low ROB	Unclear

Mikhail, 2007 [151]	2000-2004	Chronic liver disease patients undergoing endoscopy	149	-	10.2	High	High ROB	Low ROB	High ROB
**Special clinical populations**									
Hassan, 2013 [204]	-	Stem cell transplant patients	50	4.0	-	Low	High ROB	Unclear	Unclear
**Mother-to-child transmission**									
Abdul-Qawi, 2010 [22]	2003-2008	Infants of HCV Ab+ and RNA+ mothers	53	3.8	-	Low	High ROB	Low ROB	Unclear
Abo Elmagd, 2011 [205]	-	Infants of HCV Ab+ and/or RNA+ mothers	8	25	-	Low	High ROB	Low ROB	Unclear
El-Sayed Zaki, 2013 [206]	2012-2013	Infants of HCV Ab+ mothers	12	8.3	-	Low	High ROB	Low ROB	Unclear
Kassem, 2000 [58]	1996	Infants of HCV Ab+ and RNA+ mothers	14	36	-	Low	High ROB	Low ROB	Unclear
Kumar, 1997 [95]	1994-1996	Infants of HCV Ab+ and RNA+ mothers	65	24.6	-	Low	High ROB	Low ROB	Unclear
Shebl, 2009 [33]	1998-2001	Infants of HCV Ab+ and/or RNA+ mothers	232	3.4	-	High	High ROB	Low ROB	High ROB

*Studies with missing information for any of the domains were classified as having unclear ROB for that specific domain.
**Abbreviations: Ab, antibody; +, positive.

Supplementary Table S7. Precision and risk of bias (ROB) assessment for individual hepatitis C virus (HCV) antibody prevalence measures among the general population (populations at low risk) in Egypt.

First author, year of publication [citation]	Year(s) of data collection	Population	Sample size	HCV prev	Precision	Sampling	HCV ascertainment	Response rate
**General population in Egypt (n=108)**								
El-Kamary, 2015 [2]	2012-13	Pregnant women	1,250	4.2%	High	High ROB	Low ROB	Low ROB
Badr, 2015 [3]	2013-14	Blood donors	33,921	1.8%	High	High ROB	Low ROB	Low ROB
Jhaveri, 2015 [4]	2012-14	Pregnant women	2,514	3.9%	High	High ROB	Low ROB	Unclear
Abd Elrazek, 2014 [5]	2004-13	Patients attending medical center	6,660	15.3%	High	High ROB	Low ROB	Unclear
Abdel Messih, 2014 [6]	2010-11	Blood donors	17,118	3.8%	High	High ROB	Low ROB	Low ROB
Edris, 2014 [7]	2011-12	Household survey residents	2,977	9.3%	High	Low ROB	Low ROB	Unclear
Farghaly, 2014 [8]	-	Healthy children	50	6.0%	Low	High ROB	Low ROB	Unclear
Hussein, 2014 [9]	2006-12	Blood donors	308,762	4.3%	High	High ROB	Low ROB	Low ROB
Khamis, 2014 [10]	-	Pregnant women	360	6.1%	High	High ROB	Low ROB	Unclear
El-Shanshory, 2013 [11]	2010-11	Healthy blood donors	3,756	17.5%	High	High ROB	Low ROB	Low ROB
Badr, 2013 [12]	2012	Family replacement blood donors	2,847	8.1%	High	High ROB	Low ROB	Low ROB
Badr, 2013 [12]	2012	Voluntary blood donors	5,145	4.9%	High	High ROB	Low ROB	Low ROB
Hamed, 2012 [13]	2010-11	Blood donors	7,340	2.3%	High	High ROB	Unclear	Low ROB
Hussein, 2012 [14]	2006-11	Volunteer and family replacement blood donors	259,620	4.6%	High	High ROB	Unclear	Low ROB
Maha, 2012 [15]	2011	Voluntary blood donors	10,598	4.8%	High	High ROB	Low ROB	Low ROB
Maha, 2012 [15]	2011	Family replacement blood donors	4,626	8.7%	High	High ROB	Low ROB	Low ROB
Mansour, 2012 [16]	-	Healthy controls	100	0.0%	High	High ROB	Low ROB	Unclear
Schiefelbein, 2012 [17]	2007-09	Healthy controls	148	49.3%	High	High ROB	Low ROB	High ROB
Awadalla, 2011 [18]	-	Blood donors	1,000	16.8%	High	High ROB	Low ROB	Low ROB
Barakat, 2011 [19]	2005	School children	500	5.8%	High	Low ROB	Low ROB	Unclear
Teruya J, 2011 [20]	2011-unknown	Blood donors	3,425	1.6%	High	High ROB	Unclear	Low ROB
Wasfi, 2011 [21]	2007-08	Unpaid voluntary donors	3,420	3.5%	High	High ROB	Low ROB	Low ROB
AbdulQawi, 2010 [22]	2003-08	Pregnant women	1,224	8.6%	High	High ROB	Low ROB	Unclear
El-Karaksy, 2010 [23]	2006-07	Healthy children controls	1,042	1.4%	High	High ROB	Low ROB	Unclear
Khattab, 2010 [24]	2000-08	Voluntary blood donors	211,772	9.0%	High	High ROB	Low ROB	Low ROB
Mostafa, 2010 [25]	2001-06	Village residents: >4 years	4,129	12.3%	High	High ROB	Low ROB	Low ROB
Ashour, 2009 [26]	2006-08	Voluntary non-remunerated blood donors	515,758	4.8%	High	High ROB	Low ROB	Low ROB
Eita, 2009 [27]	2005-08	Voluntary blood donors	73,431	4.6%	High	High ROB	Low ROB	Low ROB
Eita, 2009 [27]	2005-08	Family replacement blood donors	113,504	5.5%	High	High ROB	Low ROB	Low ROB
Elkareh, 2009 [28]	2008	Family replacement blood donors	4,709	12.7%	High	High ROB	Low ROB	Low ROB
Elkareh, 2009 [28]	2008	Voluntary blood donors	3,569	6.3%	High	High ROB	Low ROB	Low ROB
Elkareh, 2009 [28]	2008	Family replacement blood donors	8,705	14.6%	High	High ROB	Low ROB	Low ROB
Elkareh, 2009 [28]	2008	Voluntary blood donors	414	8.7%	High	High ROB	Low ROB	Low ROB
Goldman, 2009 [29]	1999-04	Healthy controls	786	37.4%	High	High ROB	Low ROB	High ROB
Ismail, 2009 [30]	2000-07	Blood donors	55,922	12.0%	High	High ROB	Low ROB	Low ROB
Rushdy, 2009 [31]	2006-07	Blood donors	9,150	2.7%	High	High ROB	Low ROB	Low ROB
Said, 2009 [32]	-	Healthy children controls	50	0%	Low	High ROB	Low ROB	Unclear
Shebl, 2009 [33]	1997-01	Pregnant women ANC	1,863	15.7%	High	High ROB	Low ROB	Unclear
Aguilar, 2008 [34]	-	Rural village healthy residents: males	78	51%	Low	High ROB	Unclear	Unclear
Aguilar, 2008 [34]	-	Rural village healthy residents: females	81	42%	Low	High ROB	Unclear	Unclear

Elmagd, 2008 [35]	1976-04	Controls	316	49.1%	High	High ROB	Low ROB	Unclear
El-Zayadi, 2008 [36]	2005-05	Healthy volunteer blood donors	760	5%	High	High ROB	Low ROB	Low ROB
Eassa, 2007 [37]	2006-07	Household members	304	10.9%	High	High ROB	Low ROB	Unclear
Eissa, 2007 [37]	2000-05	Blood donors: 1995-2000	46,747	6.9%	High	High ROB	Low ROB	Low ROB
Eissa, 2007 [37]	2000-05	Blood donors: 2000-2005	99,757	4.0%	High	High ROB	Low ROB	Low ROB
El Damaty, 2007 [38]	1999-00	Blood donors	2,845	7.6%	High	High ROB	Low ROB	Low ROB
El Damaty, 2007 [38]	1996-97	Blood donors	861	15.2%	High	Low ROB	Low ROB	Low ROB
El-Raziky, 2007 [39]	2004-04	Children	1,042	1.4%	High	High ROB	Low ROB	Unclear
Kandil, 2007 [40]	2004-06	Children	100	38%	High	High ROB	Low ROB	Unclear
Agha, 2006 [41]	-	Blood donors	2,400	8%	High	High ROB	Low ROB	Low ROB
El-Gilany, 2006 [42]	2002-03	University student voluntary blood donors	2,157	2.7%	High	High ROB	Low ROB	Low ROB
Mohamed, 2006 [43]	2002-02	Village residents	2,425	18.5%	High	High ROB	Low ROB	High ROB
Mohamed, 2006 [43]	1997-97	Household survey members	4,631	4.8%	High	High ROB	Low ROB	Low ROB
Stoszek, 2006 [44]	1997-03	Pregnant women	2,587	15.8%	High	High ROB	Low ROB	Unclear
Arafa, 2005 [45]	2002-03	Village residents	4,020	11.8%	High	High ROB	Low ROB	High ROB
Hashish, 2005 [46]	-	Blood donors	95	23.2%	Low	High ROB	Low ROB	Low ROB
Sayed, 2005 [47]	-	Village residents	84	14.3%	Low	Low ROB	Low ROB	Unclear
Ezzat, 2005 [48]	-	Male Controls	63	30.2%	Low	High ROB	Low ROB	High ROB
Ezzat, 2005 [48]	-	Male Controls	113	45.1%	High	High ROB	Low ROB	Low ROB
Cowgill, 2004 [49]	1999-03	Controls	227	37.9%	High	High ROB	Low ROB	High ROB
el-Sadawy, 2004 [50]	-	Urban and rural residents	1,422	25.8%	High	Low ROB	Low ROB	Unclear
Tanaka,2004 [51]	1999-99	Blood donors	3,608	8.8%	High	High ROB	Low ROB	Low ROB
El-Sherbini, 2003 [52]	1994-94	School children	294	5.8%	High	High ROB	Low ROB	Unclear
Hadhoud, 2003 [53]	-	-	110	50%	High	High ROB	Unclear	Unclear
Strickland, 2002 [54]	-	Controls	212	46.7%	High	High ROB	Low ROB	Unclear
Darwish, 2001 [55]	1994-94	Semi-urban village residents aged 10 years and above	796	40%	High	High ROB	Low ROB	Low ROB
Gad, 2001 [56]	1998-98	Blood donor controls	50	20%	Low	High ROB	Low ROB	Low ROB
Abdel-Aziz, 2000 [57]	1997-97	Village residents	3,999	24.3%	High	High ROB	Low ROB	Unclear
Kassem, 2000 [58]	1996-96	Pregnant women	100	19%	High	High ROB	Low ROB	Unclear
Nafeh, 2000 [59]	-	Village household residents >5 years old	6,031	8.7%	High	High ROB	Low ROB	High ROB
Yates, 1999 [60]	-	Healthy controls	466	15.7%	High	High ROB	Low ROB	Unclear
Agha, 1998 [61]	1996-97	Pregnant women	767	13.7%	High	High ROB	Low ROB	Unclear
El-Zayadi, 1998 [62]	-	Blood donors	75	16%	Low	High ROB	Low ROB	Low ROB
Farghaly, 1998 [63]	-	Healthy controls	100	8%	High	High ROB	Low ROB	Unclear
Arthur, 1997 [64]	1993-93	Blood donors	2,644	24.8%	High	Low ROB	Low ROB	Low ROB
el-Sayed, 1997 [65]	1993-94	Permanent settlers	506	10.3%	High	Low ROB	Low ROB	Unclear
el-Zayadi, 1997 [66]	-	Blood donors	320	16.3%	High	High ROB	Low ROB	Low ROB
el-Zayadi, 1997 [66]	-	Blood donors	180	9.4%	High	High ROB	Low ROB	Low ROB
Al Omar, 1996 [67]	1993-94	Blood donors	143	34%	High	High ROB	Low ROB	Low ROB
Attia, 1996 [68]	-	Paid and unpaid blood donors	156	21.8%	High	High ROB	Low ROB	Low ROB
el-Sayed, 1996 [69]	1994-94	Workers supporting the tourist industry	740	14.3%	High	High ROB	Low ROB	Unclear
Mohamed, 1996 [70]	-	Egyptians applying work abroad	5,071	31.5%	High	High ROB	Low ROB	Unclear
Bassily, 1995 [71]	-	Blood donors	188	26.6%	High	High ROB	Low ROB	Low ROB
Darwish, 1995 [72]	-	Healthy villagers and non-professional blood donors	188	21.8%	High	High ROB	Low ROB	Low ROB
el Gohary, 1995 [73]	1990-92	Urban healthy blood donors	1,187	14.5%	High	High ROB	Low ROB	Low ROB

el Gohary, 1995 [73]	1990-92	Blood donors	271	14.4%	High	High ROB	Low ROB	Low ROB
el Gohary, 1995 [73]	1990-92	Blood donors	148	15.5%	High	High ROB	Low ROB	Low ROB
el-Nanawy, 1995 [74]	-	Healthy children controls	110	11.8%	High	High ROB	Low ROB	Unclear
Quinti, 1995 [75]	1992-94	Blood donors	283	20.8%	High	High ROB	Low ROB	Low ROB
Quinti, 1995 [75]	1995	Fire brigade personnel	541	39%	High	High ROB	Low ROB	Unclear
Abdel-Wahab, 1994 [76]	1992-92	Primary school children, 8-12 year old male students	190	12.1%	High	High ROB	Low ROB	Unclear
Abdel-Wahab, 1994 [76]	1992-92	Rural community inhabitants: >5 years old	270	18.1%	High	High ROB	Low ROB	Unclear
Abdel-Wahab, 1994 [76]	1992-92	Army recruits	300	22.1%	High	High ROB	Low ROB	Unclear
Abdel-Wahab, 1994 [76]	1992-92	Rural community inhabitants: 5-10 years old	58	0%	Low	High ROB	Low ROB	Unclear
Abdel-Wahab, 1994 [76]	1992-92	Rural community inhabitants: 10-20 years old	77	14.3%	Low	High ROB	Low ROB	Unclear
Farghaly, 1994 [77]	-	Military conscripts	726	30.4%	High	High ROB	Low ROB	Unclear
Kamel, 1994 [78]	1992-92	Village inhabitants	1,259	15.9%	High	High ROB	Low ROB	Unclear
Nooman, 1993 [79]	-	Rural village residents	1,000	0.01%	High	High ROB	Unclear	Unclear
Nooman, 1993 [79]	-	Rural village residents	2,000	0.02%	High	High ROB	Unclear	Unclear
Hassan, 1993 [80]	-	Pregnant women	1,536	4.3%	High	High ROB	Low ROB	Unclear
Khalifa, 1993 [81]	1990-91	Healthy outpatients	84	0%	Low	High ROB	Low ROB	Unclear
Darwish, 1992 [82]	-	Non-professional blood donors	90	14.4%	Low	High ROB	Low ROB	Low ROB
Zakaria, 2005 [83]	1999-02	Village residents	2,305	20.9%	High	High ROB	Low ROB	Unclear
Hagag, 1998 [84]	-	Volunteer blood donors	457	24.9%	High	Low ROB	Low ROB	Low ROB
Khedr, 1995 [85]	-	Healthy individuals controls	91	18.7%	Low	High ROB	Low ROB	Unclear
Helmy, 1995 [86]	-	Control group	110	8.1%	High	High ROB	Low ROB	Unclear
El-Zanaty, 2008 [87]	2008-08	Household survey members: 15-59 years old	11,126	14.7%	High	Low ROB	Low ROB	Low ROB
El-Zanaty, 2015 [88]	2014-15	Household survey members:1-59 years old	26,047	6.3%	High	Low ROB	Low ROB	Low ROB
**Egyptian expatriate general population (n=8)**								
Chehadeh, 2011 [89]	-	Egyptian residents of Kuwait-outpatient controls	132	3.0%	High	High ROB	Low ROB	Unclear
Derbala, 2014 [90]	2008-10	Male Egyptian residents of Qatar	2,335	13.5%	High	High ROB	Low ROB	Unclear
Perumalswami, 2014 [91]	2009-11	Egyptian residents of USA	192	15.6%	High	High ROB	Low ROB	Low ROB
Zuure, 2013 [92]	2009-10	First-generation Egyptian migrants	465	2.4%	High	High ROB	Low ROB	Low ROB
Mehdi, 2000 [93]	1995-97	Blood donors	1,609	16%	High	High ROB	Low ROB	Low ROB
Fakeeh, 1999 [94]	1998-98	Outpatients (Egyptians in KSA)	284	22.5%	High	Low ROB	Low ROB	Unclear
Kumar, 1997 [95]	1994-96	Healthy asymptomatic parturient women in the third trimester of pregnancy (Egyptians in UAE)	499	13%	High	High ROB	Low ROB	Unclear
Ahmad, 1995 [96]	-	Voluntary blood donors (Egyptians in KSA)	114	27.2%	High	High ROB	Low ROB	Low ROB
Al-Knawy, 1995 [97]	-	Unpaid male volunteer blood donors (Egyptians in KSA)	204	21.1%	High	High ROB	Low ROB	Low ROB

*Studies with missing information for any of the domains were classified as having unclear ROB for that specific domain.
**Abbreviations: ANC, antenatal clinic; Prev, prevalence; USA, United States of America; KSA, Kingdom of Saudi Arabia; UAE, United Arab Emirates.

Supplementary Table S8. Precision and risk of bias (ROB) assessment for individual hepatitis C virus (HCV) antibody prevalence measures among populations at high risk in Egypt**.**

First author, year of publication [citation]	Year(s) of data collection	Population	Sample size	HCV prev	Precision	Sampling	HCV ascertainment	Response rate
**Populations at high risk (n=35)**								
Adly, 2015 [98]	-	Thalassemia patients	176	42%	High	High ROB	Unclear	Low ROB
Helaly, 2015 [99]	2012	Hemodialysis patients	100	34%	High	High ROB	Low ROB	Unclear
Salama, 2015 [100]	-	Thalassemia patients	80	50%	Low	High ROB	Low ROB	Unclear
Hussein, 2014 [101]	2007-08	Thalassemia patients	200	24%	High	High ROB	Low ROB	Unclear
Zahran, 2014 [102]	-	Hemodialysis patients	514	49.6%	High	High ROB	Unclear	Low ROB
El Sayed Zaki, 2013 [103]	-	Children with thalassemia	100	15.0%	High	High ROB	Low ROB	Unclear
Elalfy, 2013 [104]	2009-10	Young thalassemia patients	51	82.0%	Low	High ROB	Low ROB	Unclear
El-Shanshory, 2013 [11]	2010-11	Children with thalassemia	125	76.0%	High	High ROB	Low ROB	Low ROB
Said, 2013 [105]	-	Thalassemia patients	137	34.4%	High	High ROB	Low ROB	Unclear
Tantawy, 2013 [106]	-	Children and young adults with thalassemia	84	82.0%	Low	High ROB	Low ROB	Unclear
Kamal, 2013 [107]	2011	Hemodialysis patients	170	60.6%	High	High ROB	Unclear	Unclear
Abdelwahab, 2012 [108]	-	Hemophiliac children	100	40.0%	High	High ROB	Low ROB	Unclear
El-Faramawy, 2012 [109]	-	Multi-transfused children	100	45.0%	High	High ROB	Low ROB	Unclear
Khodir, 2012 [110]	2011	Hemodialysis patients	2,351	35.0%	High	High ROB	Low ROB	Unclear
Mansour, 2012 [16]	-	Thalassemia patients	200	19.5%	High	High ROB	Low ROB	Unclear
Omar, 2011 [111]	-	Thalassemia patients	174	51.7%	High	High ROB	Low ROB	Unclear
El-Waseef, 2010 [112]	2003	Multi-transfused children	80	31.3%	Low	High ROB	Low ROB	Unclear
Ibrahim, 2010 [113]	2007	Hemodialysis patients	100	70.0%	High	High ROB	Low ROB	Low ROB
Attia, 2010 [114]	2008-09	Uremic adults and children undergoing hemodialysis	206	46.1%*	High	High ROB	Unclear	Unclear
Said, 2009 [32]		Multi-transfused children	51	17.6%	Low	High ROB	Low ROB	Unclear
Khalifa, 2004 [115]	2000-03	Children with thalassemia	56	69.6%	Low	High ROB	Unclear	Unclear
Shatat, 2000 [116]	1999-99	Hemodialysis patients	65	78.5%	Low	High ROB	Unclear	Unclear
Abdel Hady, 1998 [117]	-	Hemodialysis patients	96	27.1%	Low	High ROB	Low ROB	Unclear
el Gohary, 1995 [73]	1990-92	Hemodialysis patients	108	70.4%	High	High ROB	Low ROB	Unclear
el-Ghazzawi, 1995 [118]	-	Intravenous drug addicts	100	63%	High	High ROB	Low ROB	Unclear
Gohar, 1995 [119]	-	Hemodialysis patients	64	87.5%	Low	High ROB	Low ROB	Unclear
Abdel-Wahab, 1994 [76]	1992-92	Multi-transfused children	71	54.9%	Low	High ROB	Low ROB	Unclear
Abdel-Wahab, 1994 [76]	1992-92	Hemodialysis patients	78	46.2%	Low	High ROB	Low ROB	Unclear
Hassan, 1993 [120]	1991-93	Renal dialysis patients	105	67%	High	High ROB	Low ROB	Unclear
Khalifa, 1993 [81]	1990-91	Thalassemia patients	84	55%	Low	High ROB	Low ROB	Unclear
Goher, 1998 [121]	-	Hemodialysis patients	335	71.9%	High	High ROB	Low ROB	Unclear
Saddik, 1997 [122]	-	Hemodialysis patients	50	72%	Low	High ROB	Low ROB	Unclear
El Alfy, 1997 [104]	-	Thalassemia patients	135	8.9%	High	High ROB	Low ROB	Unclear
Helmy, 1995 [86]	-	Renal dialysis patients	250	68%	High	High ROB	Low ROB	Unclear
Helmy, 1995 [86]	-	Renal dialysis patients	100	98%	High	High ROB	Low ROB	Unclear

[*]Studies with missing information for any of the domains were classified as having unclear ROB for that specific domain.
[**]Abbreviation: Prev, prevalence.

Supplementary Table S9. Precision and risk of bias (ROB) assessment for individual hepatitis C virus (HCV) antibody prevalence measures among populations at intermediate risk, populations with liver-related conditions, and special clinical populations in Egypt.

First author, year of publication [citation]	Year(s) of data collection	Population	Sample size	HCV prev	Precision	Sampling	HCV ascertainment	Response rate
**Populations at intermediate risk (n=31)**								
El-bendary, 2015 [123]	-	Household contacts of index patients	321	13.7%	High	High ROB	Unclear	Unclear
Okasha, 2015 [124]	2008	Health care workers	1,770	8.1%	High	High ROB	Low ROB	High ROB
Farghaly, 2014 [8]	-	Diabetic children	150	12.0%	High	High ROB	Low ROB	Unclear
Elwan, 2013 [125]	-	Household contacts of index patients	481	12.7%	High	High ROB	Low ROB	Unclear
Mohamed, 2013 [126]	-	Prisoners	500	15.8%	High	Low ROB	Low ROB	High ROB
Said, 2013 [105]	-	Household contacts of index patients	73	19.2%	Low	High ROB	Low ROB	Unclear
Said, 2013 [105]	-	Household contacts of index patients	82	4.9%	Low	High ROB	Low ROB	Unclear
Munier, 2013 [127]	2008-10	Health care workers	597	7.2%	High	High ROB	Low ROB	Unclear
Tantawy, 2013 [128]	2010	Inpatients (neutropenia children)	200	0.0%	High	High ROB	Low ROB	Unclear
Abdelwahab S, 2012 [129]	2008-10	Health care workers	842	16.6%	High	High ROB	Low ROB	High ROB
Morad, 2011 [130]	-	Spouses of index patients	200	35.5%	High	High ROB	Low ROB	Unclear
El-Karaksy, 2010 [23]	2006-07	Diabetic children	692	3.6%	High	High ROB	Low ROB	Unclear
Shalaby, 2010 [131]	2007	Barbers and their clients'	616	12.5%	High	Low ROB	Low ROB	Low ROB
Elmagd, 2008 [35]	2008	Diabetic patients	286	60.3%	High	High ROB	Low ROB	Unclear
Attallah, 2004 [132]	2004	Hospitalized patients	73	90%	Low	High ROB	Low ROB	Unclear
Madwar, 1999 [133]	1999	Spouses of index patients	200	14%	High	High ROB	Low ROB	Unclear
Madwar, 1999 [133]	1999	Household contacts of index patients	355	0.0%	High	High ROB	Low ROB	Unclear
Ali, 1998 [134]	1998	Patients attending STD clinic	95	8.4%	Low	High ROB	Unclear	Unclear
Farghaly, 1998 [63]	1998	Patients with periodontal disease	100	13%	High	High ROB	Low ROB	Unclear
El-Zayadi, 1997 [135]	1997	Household contacts	265	5.7%	High	High ROB	Low ROB	Unclear
el Gohary, 1995 [73]	1995	Health care workers	78	7.7%	Low	High ROB	Low ROB	Unclear
Hindy, 1995 [136]	1995	Dentists	105	6.7%	High	High ROB	Low ROB	Unclear
Quinti, 1995 [75]	1995	Prisoners	124	31.4%	High	High ROB	Low ROB	Unclear
Hassan, 1993 [120]	1993	Patients attending STD clinic	83	10%	Low	High ROB	Low ROB	Unclear
Khalifa, 1993 [81]	1993	Hospitalized populations	84	0%	Low	High ROB	Low ROB	Unclear
Hassane, 1998 [137]	1998	Household contacts of index patients	96	17.7%	Low	High ROB	Low ROB	Unclear
Hassane, 1998 [137]	1998	Household contacts of index patients	99	9.1%	Low	High ROB	Low ROB	Unclear
Hassane, 1998 [137]	1998	Household contacts of index patients	86	27.9%	Low	High ROB	Low ROB	Unclear
Hassane, 1998 [137]	1998	Household contacts of index patients	81	13.6%	Low	High ROB	Low ROB	Unclear
Khedr, 1995 [85]	1995	Diabetic patients	109	26.6%	High	High ROB	Low ROB	Unclear
Ismail,1994 [138]	1994	Health care workers	50	0%	Low	High ROB	Low ROB	Unclear
**Egyptian expatriate population at intermediate risk (n=2)**								
Chehadeh, 2011 [89]	-	Egyptian residents of Kuwait with type II diabetes mellitus	113	18.0%	High	High ROB	Low ROB	Unclear
**Populations with liver-related conditions (n=45)**								
Gad, 2015 [139]	2011	Patients undergoing endoscopy	103	96.1%	High	High ROB	Unclear	Unclear
El Azm, 2013 [140]	2009-12	Hepatocellular carcinoma patients	281	66.2%	High	High ROB	Low ROB	Low ROB
Shaker, 2013 [141]	2009-11	Hepatocellular carcinoma patients	1,313	91.3%	High	High ROB	Unclear	Low ROB
Badawy, 2012 [142]	-	Acute viral hepatitis patients	99	8.0%	Low	High ROB	Low ROB	Unclear
EL-Meteini, 2012 [143]	2002-11	Hepatocellular carcinoma patients	146	95.2%	High	High ROB	Unclear	Low ROB
Schiefelbein, 2012 [17]	2007-09	Liver cancer patients	148	89.2%	High	High ROB	Low ROB	High ROB
Taha, 2012 [144]	2007	Hepatocellular carcinoma patients	1,643	70.0%	High	High ROB	Unclear	Unclear
El Sayed Zaki, 2011 [145]	2009-10	Acute on chronic liver failure patients	100	100.0%	High	High ROB	Low ROB	Unclear
Eldin, 2010 [146]	2007-08	Acute hepatitis patients	235	4.3%	High	High ROB	Low ROB	Unclear
Talaat, 2010 [147]	2001-04	Suspected acute viral hepatitis patients	4,189	29.8%	High	High ROB	Low ROB	Unclear
Goldman, 2009 [29]	1999-04	Non-Hodgkin's Lymphoma patients	296	47.0%	High	High ROB	Low ROB	Low ROB
Youssef, 2009 [148]	-	Patients with liver complaints	214	72.9%	High	High ROB	Low ROB	Unclear
Abdel-Wahab, 2008 [149]	2005-06	Hepatocellular carcinoma patients	80	70%	Low	High ROB	Low ROB	Unclear
Abdel Wahab, 2007 [150]	1992-05	Hepatocellular carcinoma patients	1,012	79.6%	High	High ROB	Low ROB	Low ROB

Mikhail, 2007 [151]	2000-04	Chronic liver disease patients undergoing endoscopy	859	71%	High	High ROB	Low ROB	High ROB
Zakaria, 2007 [152]	2001-02	Acute viral hepatitis patients	200	13%	High	High ROB	Low ROB	Unclear
El Gaafary, 2005 [153]	2002	Acute viral hepatitis patients	309	11%	High	High ROB	Low ROB	Unclear
el-Zayadi, 2005 [154]	1993-02	Chronic liver disease patients	22,450	72.3%	High	High ROB	Low ROB	Unclear
Ezzat, 2005 [48]	-	Hepatocellular carcinoma patients	63	87.3%	Low	High ROB	Low ROB	Low ROB
Ezzat, 2005 [48]	-	Hepatocellular carcinoma patients	113	90.3%	High	High ROB	Low ROB	Low ROB
Cowgill, 2004 [49]	1999-03	Non-Hodgkin's Lymphoma patients	227	46.7%	High	High ROB	Low ROB	High ROB
Takagi, 2003 [155]	2002	Suspected acute viral hepatitis patients	57	82.5%	Low	High ROB	Low ROB	Unclear
Zaki, 2003 [156]	-	Patients with clinically detected organomegally	65	33.8%	Low	High ROB	Unclear	Unclear
Blanton, 2002 [157]	1999-00	Hepatic Schistosomiasis	141	39.7%	High	High ROB	Low ROB	Unclear
Strickland, 2002 [54]	-	Chronic liver disease patients	237	58.2%	High	High ROB	Low ROB	Unclear
Gad, 2001 [56]	1998	Chronic liver disease patients	88	76%	Low	High ROB	Low ROB	Unclear
Rahman El-Zayadi, 2001 [158]	1992-95	Patients with liver complaints	6,850	71.1%	High	High ROB	Low ROB	Low ROB
Abdel-Wahab, 2000 [159]	1994-99	Hepatocellular carcinoma patients	385	61%	High	High ROB	Unclear	Unclear
Yates, 1999 [60]	-	Hepatocellular carcinoma patients	131	76%	High	High ROB	Low ROB	Unclear
Khalifa, 1999 [160]	-	Patients with liver cirrhosis	61	56%	Low	High ROB	Low ROB	Unclear
Angelico M, 1997 [161]	1993-95	Chronic liver disease patients	141	67.4%	High	Low ROB	Low ROB	Low ROB
Darwish, 1997 [162]	-	Hepatocellular carcinoma and patients with liver cirrhosis	94	75.5%	Low	High ROB	Low ROB	Unclear
el-Zayadi, 1997 [66]	-	Chronic liver disease patients	612	62.1%	High	High ROB	Low ROB	Unclear
el-Zayadi, 1997 [66]	-	Chronic liver disease patients	316	38.6%	High	High ROB	Low ROB	Unclear
Madwar, 1997 [163]	-	Suspected acute viral hepatitis patients	120	43.2%	High	High ROB	Unclear	Unclear
Quinti, 1997 [164]	-	Acute viral hepatitis patients	110	27.3%	High	High ROB	Low ROB	Unclear
Gomatos, 1996 [165]	1993-94	Acute viral hepatitis patients	143	8.4%	High	High ROB	Low ROB	Unclear
Waked, 1995 [166]	1992	Chronic liver disease patients	1,023	73.5%	High	High ROB	Low ROB	Unclear
Abdel-Wahab, 1994 [76]	1992	Hepatic schistosomiasis	55	16.4%	Low	High ROB	Low ROB	Unclear
Abdel-Wahab, 1994 [76]	1992	Chronic liver disease patients	354	47.2%	High	High ROB	Low ROB	Unclear
El-Gohary, 1994 [167]	-	Acute viral hepatitis patients	140	19.2%	High	High ROB	Low ROB	Unclear
Darwish, 1993 [168]	-	Hepatocellular carcinoma patients	70	30%	Low	High ROB	Low ROB	Unclear
Hassan, 1993 [120]	1991-93	Acute viral hepatitis patients	207	29%	High	High ROB	Low ROB	Unclear
Mokhtar, 1996 [169]	-	Acute viral hepatitis patients	60	6.7%	Low	High ROB	Low ROB	Unclear
Salem, 1992 [170]	-	Acute viral hepatitis patients	70	17.1%	Low	High ROB	Low ROB	Unclear
**Special clinical populations (n=27)**								
Mostafa, 2015 [171]	2012-14	Lichen planus patients	64	17.9%	Low	High ROB	Unclear	Low ROB
Gabr, 2014 [172]	2003-13	Liver transplant patients	167	16.8%	High	High ROB	Unclear	Low ROB
Mousa, 2014 [173]	-	Patients undergoing bone marrow examination	100	42.0%	High	High ROB	Low ROB	Unclear
Taha, 2014 [174]	2011-13	Patients with psoriasis vulgaris	153	13.1%	High	High ROB	Low ROB	Unclear
Abd-El-Moneim, 2013 [175]	2009-unknown	Breast cancer patients on chemotherapy	102	51.0%	High	High ROB	Unclear	Unclear
Azim, 2013 [176]	2000-08	Diffuse large B cell lymphoma patients	230	7.4%	High	High ROB	Unclear	Low ROB
El-Khalawany, 2013 [177]	2002-12	Adult patients with Tinea capitis	58	34.4%	Low	High ROB	Unclear	Unclear
Mattar, 2013 [178]	2003-13	Polycythemia vera patients	90	6.7%	Low	High ROB	Unclear	Unclear
Aboueisha, 2013 [179]	-	Living liver donors	108	7.4%	High	High ROB	Unclear	Low ROB
El Garf, 2012 [180]	2009	Rheumatoid arthritis patients	157	18.5%	High	High ROB	Low ROB	Unclear
Youssef, 2012 [181]	2010-11	Patients with non-Hodgkin lymphoma, Hodgkin disease, chronic lymphocytic leukemia	50	26.0%	Low	High ROB	Low ROB	Unclear
El Husseiny, 2011 [182]	2007-10	Myelodysplastic syndrome patients	69	13.0%	Low	High ROB	Unclear	Low ROB
El-Sabah, 2011 [183]	-	Patients on oral/parenteral anti-schistosomiasis	102	45.1%	High	High ROB	Low ROB	Unclear
Mahmoud, 2011 [184]	2009-10	Rheumatoid arthritis patients	110	20.0%	High	High ROB	Low ROB	Unclear
Abou-Zeid, 2011 [185]	-	Glomerulonephritis patients	78	59.0%	Low	High ROB	Low ROB	Unclear
Sabry, 2010 [186]	2000-03	Renal transplant patients	273	62.0%	High	High ROB	Low ROB	Low ROB
Abdel Wahab, 2007 [150]	1995-04	Hilar cholangiocarcinoma patients	440	54%	High	High ROB	Low ROB	Unclear
Sharaf-Eldeen, 2007 [187]		Lichen planus patients	100	43%	High	High ROB	Low ROB	Unclear
Sabry, 2005 [188]	-	Glomerulonephritis patients/glomerulopathy	233	38%	High	High ROB	Low ROB	Unclear
Demian, 2004 [189]	-	Patients undergoing urological procedures	667	45.1%	High	High ROB	Low ROB	Unclear

Sabry, 2002 [190]	1998-99	Glomerulonephritis patients/glomerulopathy	303	38%	High	High ROB	Low ROB	Unclear
Meir, 2001 [191]	-	Cancer patients on chemotherapy	54	19%	Low	High ROB	Low ROB	Unclear
Yates, 1999 [60]	-	Patients with bladder cancer	247	47%	High	High ROB	Low ROB	Unclear
Attia, 1996 [68]	-	Patients with various cancer	429	53.4%	High	High ROB	Low ROB	Unclear
Hassaballa, 1996 [192]	1989-94	Kidney transplant patients	54	79.6%	Low	High ROB	Low ROB	Unclear
Mostafa, 2003 [193]	2000-01	Cancer patients on chemotherapy	99	13.1%	Low	High ROB	Low ROB	Unclear
Mostafa, 2003 [193]	2000-01	Cancer patients on chemotherapy	111	39.6%	High	High ROB	Low ROB	Unclear
**Mixed populations (n=3)**								
El-Mougy, 2014 [194]	-	Hepatocellular carcinoma patients, cirrhosis patients, and healthy volunteers	58	63.8%	Low	High ROB	Low ROB	Unclear
Kalil, 2010 [195]	2004-05	Acute hepatitis, multi-transfused, non-hepatitis and non-multi transfused children	465	26.0%	High	High ROB	Low ROB	Unclear
Hammad, 2009 [196]	2008	Chronic renal failure children (hemodialysis and pre-dialysis)	100	52.0%	High	High ROB	Low ROB	Unclear

[*]Studies with missing information for any of the domains were classified as having unclear ROB for that specific domain.
[**]Abbreviations: STD, sexually transmitted diseases; Prev, prevalence.

References

1 Moher, D., Liberati, A., Tetzlaff, J., Altman, D. G. & Group, P. Preferred reporting items for systematic reviews and meta-analyses: the PRISMA statement. *PLoS medicine* **6**, e1000097, doi:10.1371/journal.pmed.1000097 (2009).

2 El-Kamary, S. S. *et al.* Reliability of risk-based screening for hepatitis C virus infection among pregnant women in Egypt. *The Journal of infection* **70**, 512-519, doi:10.1016/j.jinf.2015.01.009 (2015).

3 Farouk Badr, R., Farouk, R. & Ahmed Ali, A. Evaluation of nucleic acid testing to detect HBV infections in egyption blood donors. *Vox Sanguinis* **109**, 210-211 (2015).

4 Jhaveri, R. *et al.* Hepatitis C Virus (HCV) Vertical Transmission in 12-Month-Old Infants Born to HCV-Infected Women and Assessment of Maternal Risk Factors. *Open forum infectious diseases* **2**, ofv089, doi:10.1093/ofid/ofv089 (2015).

5 Abd Elrazek, A. E., Bilasy, S. E., Elbanna, A. E. & Elsherif, A. E. Prior to the oral therapy, what do we know about HCV-4 in Egypt: a randomized survey of prevalence and risks using data mining computed analysis. *Medicine* **93**, e204, doi:10.1097/md.0000000000000204 (2014).

6 Abdel Messih, I. Y., Ismail, M. A., Saad, A. A. & Azer, M. R. The degree of safety of family replacement donors versus voluntary non-remunerated donors in an Egyptian population: a comparative study. *Blood transfusion = Trasfusione del sangue* **12**, 159-165, doi:10.2450/2012.0115-12 (2014).

7 Edris, A. *et al.* Seroprevalence and risk factors for hepatitis B and C virus infection in Damietta Governorate, Egypt. *Eastern Mediterranean health journal = La revue de sante de la Mediterranee orientale = al-Majallah al-sihhiyah li-sharq al-mutawassit* **20**, 605-613 (2014).

8 Farghaly, H. S., Metwalley, K. A. & El-Hafeez, H. A. Hepatitis C virus infection in Egyptian children with type 1 diabetes mellitus: A single center study. *Indian journal of endocrinology and metabolism* **18**, 197-201, doi:10.4103/2230-8210.129111 (2014).

9 Hussein, E. Blood donor recruitment strategies and their impact on blood safety in Egypt. *Transfusion and apheresis science : official journal of the World Apheresis Association : official journal of the European Society for Haemapheresis* **50**, 63-67, doi:10.1016/j.transci.2013.11.005 (2014).

10 Khamis, H. H., Farghaly, A. G., Shatat, H. Z. & El-Ghitany, E. M. Prevalence of hepatitis C virus infection among pregnant women in a rural district in Egypt. *Tropical doctor*, doi:10.1177/0049475514561330 (2014).

11 El-Shanshory, M. R., Kabbash, I. A., Soliman, H. H., Nagy, H. M. & Abdou, S. H. Prevalence of hepatitis C infection among children with beta-thalassaemia major in Mid Delta, Egypt: a single centre study. *Transactions of the Royal Society of Tropical Medicine and Hygiene* **107**, 224-228, doi:10.1093/trstmh/trs024 (2013).

12 Farouk Badr, R. Safety of vnrbds versus family replacement blood donors in Egypt, evidence based approach. *Vox Sanguinis* **105**, 104, doi:http://dx.doi.org/10.1111/vox.12048 (2013).

13 Hamed, H. Detection of occult hepatitis B virus infection and prevalence of hepatitis B surface antigen and hepatitis c virus antibodies among blood donors in south egypt. *Transfusion Medicine* **22**, 52, doi:http://dx.doi.org/10.1111/cyt.12004 (2012).

14 Hussein, E. A. Seroprevalence and trends of positive infectious markers in egyptian volunteer and family replacement donors: A five year study. *Transfusion* **52**, 103A, doi:http://dx.doi.org/10.1111/j.1537-2995.2012.03833-1.x (2012).

15 Maha, A. R., El Kareh, S., Afaf, A. & Ahmed, A. Evaluation of hcv reactive donors according to type of donor, gender (SEX) and age groups at shebin EL KOM regional blood transfusion center (RBTC) (from january 2011 to december 2011). *Vox Sanguinis* **103**, 171, doi:http://dx.doi.org/10.1111/j.1423-0410.2012.01615-2.x (2012).

16 Mansour, A. K. *et al.* Prevalence of HBV and HCV infection among multi-transfused Egyptian thalassemic patients. *Hematology/oncology and stem cell therapy* **5**, 54-59, doi:10.5144/1658-3876.2012.54 (2012).

17 Schiefelbein, E. *et al.* Hepatitis C virus and other risk factors in hepatocellular carcinoma. *Acta virologica* **56**, 235-240 (2012).

18 Awadalla, H. I., Ragab, M. H., Nassar, N. A. & Osman, M. A. Risk factors of hepatitis C infection among Egyptian blood donors. *Central European journal of public health* **19**, 217-221 (2011).

19 Barakat, S. H. & El-Bashir, N. Hepatitis C virus infection among healthy Egyptian children: prevalence and risk factors. *Journal of viral hepatitis* **18**, 779-784, doi:10.1111/j.1365-2893.2010.01381.x (2011).
20 Teruya, J. & Hussein, E. A. Blood supply operation during egyptian revolution. *Transfusion* **51**, 249A, doi:http://dx.doi.org/10.1111/j.1537-2995.2011.03301_2.x (2011).
21 Wasfi, O. A. & Sadek, N. A. Prevalence of hepatitis B surface antigen and hepatitis C virus antibodies among blood donors in Alexandria, Egypt. *Eastern Mediterranean health journal = La revue de sante de la Mediterranee orientale = al-Majallah al-sihhiyah li-sharq al-mutawassit* **17**, 238-242 (2011).
22 AbdulQawi, K. *et al.* Prospective study of prevalence and risk factors for hepatitis C in pregnant Egyptian women and its transmission to their infants. *Croatian medical journal* **51**, 219-228 (2010).
23 El-Karaksy, H. *et al.* Anti-HCV prevalence among diabetic and non-diabetic Egyptian children. *Current diabetes reviews* **6**, 388-392 (2010).
24 Khattab, M. A., Eslam, M., Sharwae, M. A. & Hamdy, L. Seroprevalence of hepatitis C and B among blood donors in Egypt: Minya Governorate, 2000-2008. *American journal of infection control* **38**, 640-641, doi:10.1016/j.ajic.2009.12.016 (2010).
25 Mostafa, A. *et al.* Is the hepatitis C virus epidemic over in Egypt? Incidence and risk factors of new hepatitis C virus infections. *Liver international : official journal of the International Association for the Study of the Liver* **30**, 560-566, doi:10.1111/j.1478-3231.2009.02204.x (2010).
26 Ashour, D., Moftah, F., Gobran, H. & Ekram, D. Decreasing the risk of transfusion transmitted infections (TTIS) in the Egyptian Blood Transfusion Services. *Vox Sanguinis* **96**, 103-104, doi:http://dx.doi.org/10.1111/j.1423-0410.2009.01156.x (2009).
27 Eita, N. Prevalence of HCV and HBV infections among blood donors in Dakahilia, Egypt. *Vox Sanguinis* **96**, 106-107, doi:http://dx.doi.org/10.1111/j.1423-0410.2009.01156.x (2009).
28 Elkareh, S. HCv screening of donors in Governmental Blood Transfusion Centers at Menoufia Governorate (from Jan. 2008 to Oct. 2008). *Vox Sanguinis* **96**, 89-90, doi:http://dx.doi.org/10.1111/j.1423-0410.2009.01156.x (2009).
29 Goldman, L. *et al.* Viral and non-viral risk factors for non-Hodgkin's lymphoma in Egypt: heterogeneity by histological and immunological subtypes. *Cancer causes & control : CCC* **20**, 981-987, doi:10.1007/s10552-009-9316-0 (2009).
30 Ismail, A. M., Ziada, H. N., Sheashaa, H. A. & Shehab El-Din, A. B. Decline of viral hepatitis prevalence among asymptomatic Egyptian blood donors: a glimmer of hope. *European journal of internal medicine* **20**, 490-493, doi:10.1016/j.ejim.2009.03.005 (2009).
31 Rushdy, O., Moftah, F. & Zakareya, S. Trasmitted transfused viral infections among blood donors during years 2006 and 2007 in suez canal area, Egypt. *Vox Sanguinis* **96**, 86-87, doi:http://dx.doi.org/10.1111/j.1423-0410.2009.01156.x (2009).
32 Said, Z. N. *et al.* High prevalence of occult hepatitis B in hepatitis C-infected Egyptian children with haematological disorders and malignancies. *Liver international : official journal of the International Association for the Study of the Liver* **29**, 518-524, doi:10.1111/j.1478-3231.2009.01975.x (2009).
33 Shebl, F. M. *et al.* Prospective cohort study of mother-to-infant infection and clearance of hepatitis C in rural Egyptian villages. *Journal of medical virology* **81**, 1024-1031, doi:10.1002/jmv.21480 (2009).
34 Aguilar, C. E. *et al.* Androgen profiles among Egyptian adults considering liver status. *Journal of gastroenterology and hepatology* **23**, e137-145, doi:10.1111/j.1440-1746.2007.04949.x (2008).
35 Elmagd, M. M., Bakr, M. A., Metwally, A. H. & Wahab, A. M. Clinicoepidemiologic study of posttransplant diabetes after living-donor renal transplant. *Experimental and clinical transplantation : official journal of the Middle East Society for Organ Transplantation* **6**, 42-47 (2008).
36 El-Zayadi, A. R. *et al.* Anti-HBc screening in Egyptian blood donors reduces the risk of hepatitis B virus transmission. *Transfusion medicine (Oxford, England)* **18**, 55-61, doi:10.1111/j.1365-3148.2007.00806.x (2008).
37 Eassa, S., Eissa, M., Sharaf, S. M., Ibrahim, M. H. & Hassanein, O. M. Prevalence of hepatitis C virus infection and evaluation of a health education program in el-ghar village in zagazig, egypt. *The Journal of the Egyptian Public Health Association* **82**, 379-404 (2007).
38 El Damaty, S. I. *et al.* Surveillance system for HCV infection: testing a model based on blood banks. *The Journal of the Egyptian Public Health Association* **82**, 451-471 (2007).

39 El-Raziky, M. S. *et al.* Prevalence and risk factors of asymptomatic hepatitis C virus infection in Egyptian children. *World journal of gastroenterology : WJG* **13**, 1828-1832 (2007).
40 Kandil, M. E., Rasheed, M. A. & Saad, N. E. Hepatitis C and B viruses among some high risk groups of Egyptian children. *Journal of Medical Sciences* **7**, 1259-1267 (2007).
41 Agha, S. *et al.* Prevalence of low positive anti-HCV antibodies in blood donors: Schistosoma mansoni co-infection and possible role of autoantibodies. *Microbiology and immunology* **50**, 447-452 (2006).
42 El-Gilany, A. H. & El-Fedawy, S. Bloodborne infections among student voluntary blood donors in Mansoura University, Egypt. *Eastern Mediterranean health journal = La revue de sante de la Mediterranee orientale = al-Majallah al-sihhiyah li-sharq al-mutawassit* **12**, 742-748 (2006).
43 Mohamed, M. K. *et al.* HCV-related morbidity in a rural community of Egypt. *Journal of medical virology* **78**, 1185-1189, doi:10.1002/jmv.20679 (2006).
44 Stoszek, S. K. *et al.* Prevalence of and risk factors for hepatitis C in rural pregnant Egyptian women. *Transactions of the Royal Society of Tropical Medicine and Hygiene* **100**, 102-107, doi:10.1016/j.trstmh.2005.05.021 (2006).
45 Arafa, N. *et al.* Changing pattern of hepatitis C virus spread in rural areas of Egypt. *J Hepatol* **43**, 418-424, doi:10.1016/j.jhep.2005.03.021 (2005).
46 Hashish, M. H., El-Barrawy, M. A., Mahmoud, O. A. & Abdel Rahman, N. W. TT virus among blood donors in Alexandria. *The Journal of the Egyptian Public Health Association* **80**, 651-664 (2005).
47 Sayed, H. A. *et al.* A cross sectional study of hepatitis B, C, some trace elements, heavy metals, aflatoxin B1 and schistosomiasis in a rural population, Egypt. *The Journal of the Egyptian Public Health Association* **80**, 355-388 (2005).
48 Ezzat, S. *et al.* Associations of pesticides, HCV, HBV, and hepatocellular carcinoma in Egypt. *International journal of hygiene and environmental health* **208**, 329-339 (2005).
49 Cowgill, K. D. *et al.* Case-control study of non-Hodgkin's lymphoma and hepatitis C virus infection in Egypt. *International journal of epidemiology* **33**, 1034-1039, doi:10.1093/ije/dyh183 (2004).
50 el-Sadawy, M. *et al.* Hepatitis C virus infection at Sharkia Governorate, Egypt: seroprevalence and associated risk factors. *Journal of the Egyptian Society of Parasitology* **34**, 367-384 (2004).
51 Tanaka, Y. *et al.* Exponential spread of hepatitis C virus genotype 4a in Egypt. *Journal of molecular evolution* **58**, 191-195, doi:10.1007/s00239-003-2541-3 (2004).
52 El-Sherbini, A., Hassan, W., Abdel-Hamid, M. & Naeim, A. Natural history of hepatitis C virus among apparently normal schoolchildren: follow-up after 7 years. *Journal of tropical pediatrics* **49**, 384-385 (2003).
53 Hadhoud, A. *et al.* The relationship between HLA typing and HCV infection and outcome of renal transplantation in HCV positive patients. *Experimental and clinical transplantation : official journal of the Middle East Society for Organ Transplantation* **1**, 19-25 (2003).
54 Strickland, G. T. *et al.* Role of hepatitis C infection in chronic liver disease in Egypt. *The American journal of tropical medicine and hygiene* **67**, 436-442 (2002).
55 Darwish, M. A. *et al.* Hepatitis c and cirrhotic liver disease in the Nile delta of Egypt: a community-based study. *The American journal of tropical medicine and hygiene* **64**, 147-153 (2001).
56 Gad, A. *et al.* Relationship between hepatitis C virus infection and schistosomal liver disease: not simply an additive effect. *Journal of gastroenterology* **36**, 753-758 (2001).
57 Abdel-Aziz, F. *et al.* Hepatitis C virus (HCV) infection in a community in the Nile Delta: population description and HCV prevalence. *Hepatology* **32**, 111-115, doi:10.1053/jhep.2000.8438 (2000).
58 Kassem, A. S., el-Nawawy, A. A., Massoud, M. N., el-Nazar, S. Y. & Sobhi, E. M. Prevalence of hepatitis C virus (HCV) infection and its vertical transmission in Egyptian pregnant women and their newborns. *Journal of tropical pediatrics* **46**, 231-233 (2000).
59 Nafeh, M. A. *et al.* Hepatitis C in a community in Upper Egypt: I. Cross-sectional survey. *The American journal of tropical medicine and hygiene* **63**, 236-241 (2000).
60 Yates, S. C. *et al.* Hepatocellular carcinoma in Egyptians with and without a history of hepatitis B virus infection: association with hepatitis C virus (HCV) infection but not with (HCV) RNA level. *The American journal of tropical medicine and hygiene* **60**, 714-720 (1999).
61 Agha, S., Sherif, L. S., Allam, M. A. & Fawzy, M. Transplacental transmission of hepatitis C virus in HIV-negative mothers. *Research in virology* **149**, 229-234 (1998).

62 El-Zayadi, A., Osaima, S., Hess, G., Zdunek, D. & Ahdy, A. Seroprevalence of anti-HGV E2 and HGV RNA among Egyptian blood donors. *LaboratoriumsMedizin* **22**, 363-365 (1998).
63 Farghaly, A. G., Mansour, G. A., Mahdy, N. H. & Yousri, A. Hepatitis B and C virus infections among patients with gingivitis and adult periodontitis: seroprevalence and public health importance. *The Journal of the Egyptian Public Health Association* **73**, 707-735 (1998).
64 Arthur, R. R. *et al.* Hepatitis C antibody prevalence in blood donors in different governorates in Egypt. *Transactions of the Royal Society of Tropical Medicine and Hygiene* **91**, 271-274 (1997).
65 el-Sayed, H. F., Abaza, S. M., Mehanna, S. & Winch, P. J. The prevalence of hepatitis B and C infections among immigrants to a newly reclaimed area endemic for Schistosoma mansoni in Sinai, Egypt. *Acta tropica* **68**, 229-237 (1997).
66 el-Zayadi, A. R. *et al.* Does schistosomiasis play a role in the high sero prevalence of HCV antibody among Egyptians? *Tropical gastroenterology : official journal of the Digestive Diseases Foundation* **18**, 98-100 (1997).
67 Al Omar, A. S. & El Zuebi, F. Disease markers in blood donors at King Fahad Hospital, Al Baha. *Annals of Saudi medicine* **16**, 37-41 (1996).
68 Attia, M. A. *et al.* Diverse patterns of recognition of hepatitis C virus core and nonstructural antigens by antibodies present in Egyptian cancer patients and blood donors. *Journal of clinical microbiology* **34**, 2665-2669 (1996).
69 el-Sayed, N. M. *et al.* Seroprevalence survey of Egyptian tourism workers for hepatitis B virus, hepatitis C virus, human immunodeficiency virus, and Treponema pallidum infections: association of hepatitis C virus infections with specific regions of Egypt. *The American journal of tropical medicine and hygiene* **55**, 179-184 (1996).
70 Mohamed, M. K. *et al.* Study of the risk factors for viral hepatitis C infection among Egyptians applying for work abroad. *The Journal of the Egyptian Public Health Association* **71**, 113-147 (1996).
71 Bassily, S., Hyams, K. C., Fouad, R. A., Samaan, M. D. & Hibbs, R. G. A high risk of hepatitis C infection among Egyptian blood donors: the role of parenteral drug abuse. *The American journal of tropical medicine and hygiene* **52**, 503-505 (1995).
72 Darwish, N. M., Abbas, M. O., Hady, S. I. & Mohammed, T. A. Study of the high prevalence of HCV in Egypt. *The Journal of the Egyptian Public Health Association* **70**, 397-414 (1995).
73 el Gohary, A. *et al.* High prevalence of hepatitis C virus among urban and rural population groups in Egypt. *Acta tropica* **59**, 155-161 (1995).
74 el-Nanawy, A. A. *et al.* Prevalence of hepatitis-C antibody seropositivity in healthy Egyptian children and four high risk groups. *Journal of tropical pediatrics* **41**, 341-343 (1995).
75 Quinti, I. *et al.* Seroprevalence of HIV and HCV infections in Alexandria, Egypt. *Zentralblatt fur Bakteriologie : international journal of medical microbiology* **283**, 239-244 (1995).
76 Abdel-Wahab, M. F. *et al.* High seroprevalence of hepatitis C infection among risk groups in Egypt. *The American journal of tropical medicine and hygiene* **51**, 563-567 (1994).
77 Farghaly, A. G. & Barakat, R. M. Prevalence, impact and risk factors of hepatitis C infection in Egypt. [French]

Prevalence, Impact Et Facteurs De Risques De L'hepatite C En Egypte. *Medecine et Chirurgie Digestives* **23**, 171-173 (1994).
78 Kamel, M. A. *et al.* The epidemiology of Schistosoma mansoni, hepatitis B and hepatitis C infection in Egypt. *Annals of tropical medicine and parasitology* **88**, 501-509 (1994).
79 International Conference on Schistosomiasis. The SRP 1993 - Le Caire, 14-18 Februari 1993. Hepatitis B, Hepatitis C and Schistosomiasis. [French]

International Conference on Schistosomiasis. The Srp 1993 - Le Caire, 14-18 Fevrier 1993. Hepatite B, Hepatite C Et Bilharziose. *Medecine et Chirurgie Digestives* **22**, 441-442 (1993).
80 Hassan, N. F. & Kotkat, A. Prevalence of antibodies to hepatitis C virus in pregnant women in Egypt. *The Journal of infectious diseases* **168**, 248-249 (1993).
81 Khalifa, A. S. *et al.* Prevalence of hepatitis C viral antibody in transfused and nontransfused Egyptian children. *The American journal of tropical medicine and hygiene* **49**, 316-321 (1993).
82 Darwish, N. M., Abbas, M. O., Abdelfattah, F. M. & Darwish, M. A. Hepatitis C virus infection in blood donors in Egypt. *The Journal of the Egyptian Public Health Association* **67**, 223-236 (1992).

83 Zakaria, S. *et al.* A community-based study of viral hepatitis infection in Giza Governorate, Egypt: Seroprevalence, risk factors and associated morbidity. *The Medical Journal of Cairo University* **73**, 899 (2005).
84 Hagag, S. A., Koura, S. K. & Abdel Hameed, M. F. Seroprevalence of hepatitis C virus infection among the volunteer blood donors in Zagazig. *Zagazig University Medical Journal* **4**, 199-209 (1998).
85 Khedr M.S., Abdel-Hamid, A. S., Abdel-Alla, E. M., Abdel-Moneim, S. M. & Ismail, E. F. Liver Injury in Diabetic Patients with Hepatitis C Virus Antibodies. *Scientific Medical Journal* **7**, 105-116 (1995).
86 Helmy, M. F., Abdel Aziz, G. A. W. & Ibrahim, M. S. Hepatitis C Virus Infection Among Hemodialysis Patients. *Egyptian Journal of Medical Microbiology* **4**, 289-292 (1995).
87 El-Zanaty, F. & Way, A. Egypt Demographic and Health Survey 2008., (Ministry of Health, El-Zanaty and Associates, and Macro International. , Cairo, Egypt, 2009).
88 Ministry of Health and Population [Egypt], El-Zanaty and Associates [Egypt] & and ICF International. Egypt Health Issues Survey 2015. (Cairo, Egypt and Rockville, Maryland, USA: Ministry of Health and Population and ICF International, 2015).
89 Chehadeh, W. *et al.* Hepatitis C virus infection in a population with high incidence of type 2 diabetes: impact on diabetes complications. *Journal of infection and public health* **4**, 200-206, doi:10.1016/j.jiph.2011.05.004 (2011).
90 Derbala, M. *et al.* Reexamination of the relationship between the prevalence of hepatitis C virus and parenteral antischistosomal therapy among Egyptians resident in Qatar. *Clinical and experimental gastroenterology* **7**, 427-433, doi:10.2147/ceg.s65369 (2014).
91 Perumalswami, P. V. *et al.* Hepatitis C screening beyond CDC guidelines in an Egyptian immigrant community. *Liver international : official journal of the International Association for the Study of the Liver* **34**, 253-258, doi:10.1111/liv.12259 (2014).
92 Zuure, F. R. *et al.* Screening for hepatitis B and C in first-generation Egyptian migrants living in the Netherlands. *Liver international : official journal of the International Association for the Study of the Liver* **33**, 727-738, doi:10.1111/liv.12131 (2013).
93 Mehdi, S. R., Pophali, A. & Al-Abdul Rahim, K. A. Prevalence of hepatitis B and C and blood donors. *Saudi medical journal* **21**, 942-944 (2000).
94 Fakeeh, M. & Zaki, A. M. Hepatitis C: prevalence and common genotypes among ethnic groups in Jeddah, Saudi Arabia. *The American journal of tropical medicine and hygiene* **61**, 889-892 (1999).
95 Kumar, R. M., Frossad, P. M. & Hughes, P. F. Seroprevalence and mother-to-infant transmission of hepatitis C in asymptomatic Egyptian women. *European journal of obstetrics, gynecology, and reproductive biology* **75**, 177-182 (1997).
96 Ahmad, M. S. *et al.* Prevalence of antibodies against the hepatitis C virus among voluntary blood donors at a makkah hospital. *Saudi journal of kidney diseases and transplantation : an official publication of the Saudi Center for Organ Transplantation, Saudi Arabia* **6**, 122-124 (1995).
97 Al-Knawy, B., El Mekki, A. A., Hamdi, J., Thiga, R. & Sheikha, A. Prevalence of antibody to hepatitis C virus in Saudi blood donors, PREVALENCE DE L'ANTICORPS ANTI-VIRUS DE L'HEPATITE C CHEZ DES DONNEURS DE SANG SAOUDIENS. *Canadian Journal of Gastroenterology* **9**, 141-143 (1995).
98 Adly, A. A. & Ebeid, F. S. Cultural preferences and limited public resources influence the spectrum of thalassemia in Egypt. *Journal of pediatric hematology/oncology* **37**, 281-284, doi:10.1097/mph.0000000000000327 (2015).
99 Helaly, G. F., El Ghazzawi, E. F., Shawky, S. M. & Farag, F. M. Occult hepatitis B virus infection among chronic hemodialysis patients in Alexandria, Egypt. *Journal of infection and public health*, doi:10.1016/j.jiph.2015.04.019 (2015).
100 Salama, K. M. *et al.* Liver enzymes in children with beta-Thalassemia major: Correlation with iron overload and viral hepatitis. *Macedonian Journal of Medical Sciences* **3**, 287-292, doi:http://dx.doi.org/10.3889/oamjms.2015.059 (2015).
101 Hussein, E. Evaluation of infectious disease markers in multitransfused Egyptian children with thalassemia. *Annals of clinical and laboratory science* **44**, 62-66 (2014).
102 Zahran, A. M. Prevalence of Seroconversion of Hepatitis C Virus among Hemodialysis Patients in Menoufia Governorate, Egypt. *Arab journal of nephrology and transplantation* **7**, 133-135 (2014).

103 El Sayed Zaki, M., Magdy Abd El Razek, H. & Magdy Abd El Razek, M. Hepatitis E viral seroprevalence among multiple transfused Egyptian children. *Journal of Viral Hepatitis* **20**, 40-41, doi:http://dx.doi.org/10.1111/jvh.12165 (2013).

104 Elalfy, M. S., Esmat, G., Matter, R. M., Abdel Aziz, H. E. & Massoud, W. A. Liver fibrosis in young Egyptian beta-thalassemia major patients: relation to hepatitis C virus and compliance with chelation. *Annals of hepatology* **12**, 54-61 (2013).

105 Said, F. *et al.* Intrafamilial transmission of hepatitis C infection in Egyptian multitransfused thalassemia patients. *Journal of tropical pediatrics* **59**, 309-313, doi:10.1093/tropej/fmt017 (2013).

106 Tantawy, A., El-Sherif, N. & Ismail, I. A. Autoimmune thyroid dysfunction in young egyptian transfusion-dependent b-thalassemia patients. *Haematologica* **98**, 715 (2013).

107 Kamal, N. N., Kamel, E. G., Eldessouki, K. H. & Ahmed, M. G. Health-related quality of life among hemodialysis patients at El-Minia University Hospital, Egypt. *Journal of Public Health (Germany)* **21**, 193-200, doi:http://dx.doi.org/10.1007/s10389-012-0538-3 (2013).

108 Abdelwahab, M. S., El-Raziky, M. S., Kaddah, N. A. & Abou-Elew, H. H. Prevalence of hepatitis C virus infection and human immunodeficiency virus in a cohort of Egyptian hemophiliac children. *Annals of Saudi medicine* **32**, 200-202 (2012).

109 El-Faramawy, A. A., El-Rashidy, O. F., Tawfik, P. H. & Hussein, G. H. Transfusion transmitted hepatitis: where do we stand now? A one center study in upper egypt. *Hepatitis monthly* **12**, 286-291, doi:10.5812/hepatmon.852 (2012).

110 Khodir, S. A., Alghateb, M., Okasha, K. M. & Shalaby Sel, S. Prevalence of HCV infections among hemodialysis patients in Al Gharbiyah Governorate, Egypt. *Arab journal of nephrology and transplantation* **5**, 145-147 (2012).

111 Omar, N. *et al.* Major risk of blood transfusion in hemolytic anemia patients. *Blood coagulation & fibrinolysis : an international journal in haemostasis and thrombosis* **22**, 280-284, doi:10.1097/MBC.0b013e3283451255 (2011).

112 El-Waseef, M. M., Taha, S. & Elgindi, H. Left ventricular diastolic abnormalities and the impact of hepatitis C virus infection in multitransfused Egyptian children. *Archives of medical science : AMS* **6**, 96-99, doi:10.5114/aoms.2010.13514 (2010).

113 Ibrahim, S. Quality of care assessment and adherence to the international guidelines considering dialysis, water treatment, and protection against transmission of infections in university hospital-based dialysis units in Cairo, Egypt. *Hemodialysis international. International Symposium on Home Hemodialysis* **14**, 61-67, doi:10.1111/j.1542-4758.2009.00398.x (2010).

114 Attia, E. A., Hassan, S. I. & Youssef, N. M. Cutaneous disorders in uremic patients on hemodialysis: an Egyptian case-controlled study. *Int J Dermatol* **49**, 1024-1030 (2010).

115 Khalifa, A. S. *et al.* Abnormal glucose tolerance in Egyptian beta-thalassemic patients: possible association with genotyping. *Pediatr Diabetes* **5**, 126-132, doi:10.1111/j.1399-543X.2004.00051.x (2004).

116 Shatat, H. Z., Kotkat, A. M. & Farghaly, A. G. Immune response to hepatitis B vaccine in haemodialysis patients. *The Journal of the Egyptian Public Health Association* **75**, 257-275 (2000).

117 Abdel Hady, S. I., El-Din, M. S. & El-Din, M. E. A high hepatitis E virus (HEV) seroprevalence among unpaid blood donors and haemodialysis patients in Egypt. *The Journal of the Egyptian Public Health Association* **73**, 165-179 (1998).

118 el-Ghazzawi, E. *et al.* Intravenous drug addicts: a high risk group for infection with human immunodeficiency virus, hepatitis viruses, cytomegalo virus and bacterial infections in Alexandria Egypt. *The Journal of the Egyptian Public Health Association* **70**, 127-150 (1995).

119 Gohar, S. A., Khalil, R. Y., Elaish, N. M., Khedr, E. M. & Ahmed, M. S. Prevalence of antibodies to hepatitis C virus in hemodialysis patients and renal transplant recipients. *The Journal of the Egyptian Public Health Association* **70**, 465-484 (1995).

120 Hassan, N. F. Prevalence of hepatitis C antibodies in patient groups in Egypt. *Transactions of the Royal Society of Tropical Medicine and Hygiene* **87**, 638 (1993).

121 Goher, S. A., Abdel-Ghany M.M., Shaarawy A-B & S.A., S. Dialyzer reuse and hepatitis C virus in hemodialysis population in Egypt. *Scientific Medical Journal* **10** 43-54 (1998).

122 Saddik, Y. & El Azoni, M. Hepatitis C virus [HCV] antibodies in patients with chronic renal failure and treated with regular hemodialysis and those treated with renal transplantation. *Scientific Medical Journal* **9**, 79-99 (1997).

123 El-bendary, M. *et al.* Epidemiological aspects of intrafamilial spread of HCV infection in Egyptian population a pilot study. *Hepatology International* **1)**, S65, doi:http://dx.doi.org/10.1007/s12072-015-9609-1 (2015).
124 Okasha, O. *et al.* Hepatitis C virus infection and risk factors in health-care workers at Ain Shams University Hospitals, Cairo, Egypt. *Eastern Mediterranean health journal = La revue de sante de la Mediterranee orientale = al-Majallah al-sihhiyah li-sharq al-mutawassit* **21**, 199-212 (2015).
125 Abstracts for the Viral Hepatitis Congress 2013. *Journal of Viral Hepatitis* **20** (2013).
126 Mohamed, H. I. *et al.* Hepatitis C, hepatitis B and HIV infection among Egyptian prisoners: seroprevalence, risk factors and related chronic liver diseases. *Journal of infection and public health* **6**, 186-195, doi:10.1016/j.jiph.2012.12.003 (2013).
127 Munier, A. *et al.* Frequent transient hepatitis C viremia without seroconversion among healthcare workers in Cairo, Egypt. *PloS one* **8**, e57835, doi:10.1371/journal.pone.0057835 (2013).
128 Tantawy, A. A. G., Sallam, T. H., Ibrahim, D. M., Sallam, M. T. & Ragab, I. A. Pathogenesis and prognosis of neutropenia in infants and children admitted in a university children hospital in Egypt. *Pediatric Hematology and Oncology* **30**, 51-59, doi:http://dx.doi.org/10.3109/08880018.2012.743199 (2013).
129 Abdelwahab, S. *et al.* Risk factors for hepatitis C virus infection among Egyptian healthcare workers in a national liver diseases referral centre. *Transactions of the Royal Society of Tropical Medicine and Hygiene* **106**, 98-103, doi:10.1016/j.trstmh.2011.10.003 (2012).
130 Morad, W. S. Transmission of hepatitis C between spouses an epidemiological study at national liver institute hospital. *Hepatology International* **5 (1)**, 224, doi:http://dx.doi.org/10.1007/s12072-010-9241-z (2011).
131 Shalaby, S. *et al.* Hepatitis B and C viral infection: prevalence, knowledge, attitude and practice among barbers and clients in Gharbia governorate, Egypt. *Eastern Mediterranean health journal = La revue de sante de la Mediterranee orientale = al-Majallah al-sihhiyah li-sharq al-mutawassit* **16**, 10-17 (2010).
132 Attallah, A. M. & Ibrahim, G. G. Immunodetection of a hepatitis C virus (HCV) antigen and Thl/Th2 cytokines in cerebrospinal fluid of meningitis patients. *Journal of immunoassay & immunochemistry* **25**, 313-320 (2004).
133 Madwar, M. A., El-Gindy, I., Fahmy, H. M., Shoeb, N. M. & Massoud, B. A. Hepatitis C virus transmission in family members of Egyptian patients with HCV related chronic liver disease. *The Journal of the Egyptian Public Health Association* **74**, 313-332 (1999).
134 Ali, F., Abdel-Aziz, A., Helmy, M. F., Abdel-Mobdy, A. & Darwish, M. Prevalence of certain sexually transmitted viruses in Egypt. *The Journal of the Egyptian Public Health Association* **73**, 181-192 (1998).
135 El-Zayadi, A. *et al.* Evaluation of risk factors for intrafamilial transmission of HCV infection in Egypt. *The Journal of the Egyptian Public Health Association* **72**, 33-51 (1997).
136 Hindy, A. M., Abdelhaleem, E. S. & Aly, R. H. Hepatitis B and C viruses among Egyptian dentists. *Egyptian dental journal* **41**, 1217-1226 (1995).
137 Hassane, F. M. *et al.* Horizontal Transmission of Hepatitis C Virus (HCV) in The Families of Children Positive For Hepatitis C. *Alexandria Jouranl of Pediatrics* **12**, 209-215 (1998).
138 Ismail, Z. A., Soliman, H. A., Zahran, A. M. & Kamal El Din, A. M. Prevalence of hepatitis C virus antibodies in hemodialysis patients. *The Medical Journal of Cairo University* **62**, 283-291 (1994).
139 Gad, E. H., Alsebaey, A., Lotfy, M., Eltabbakh, M. & Sherif, A. A. Complications and mortality after adult to adult living donor liver transplantation: A retrospective cohort study. *Annals of medicine and surgery (2012)* **4**, 162-171, doi:10.1016/j.amsu.2015.04.021 (2015).
140 El Azm, A. R. *et al.* Serum anti-P53 antibodies and alpha-fetoprotein in patients with non-B non-C hepatocellular carcinoma. *SpringerPlus* **2**, 69, doi:10.1186/2193-1801-2-69 (2013).
141 Shaker, O. *et al.* Osteopontin gene polymorphisms as predictors for the efficacy of interferon therapy in chronic hepatitis C Egyptian patients with genotype 4. *Cell biochemistry and function* **31**, 620-625, doi:10.1002/cbf.2954 (2013).
142 Badawy, A. A. & Khalil, H. H. The aetiology of acute viral hepatitis among some Egyptian youth. *Journal of the Egyptian Society of Parasitology* **42**, 203-206 (2012).
143 M, E. L.-M. *et al.* Single center experience over decade in living donor liver transplantation for egyptian patients with hepatocellular carcinoma: Stretching the limits. *Liver Transplantation* **18**, S230, doi:http://dx.doi.org/10.1002/lt.23435 (2012).

144 Taha, A. *et al.* Clinicoepidemiological characteristics and response to treatment in patients with hepatocellular carcinoma in Egypt. *Hepatology International* **6 (1)**, 218, doi:http://dx.doi.org/10.1007/s12072-011-9333-4 (2012).
145 El Sayed Zaki, M. & Othman, W. Role of hepatitis E infection in acute on chronic liver failure in Egyptian patients. *Liver international : official journal of the International Association for the Study of the Liver* **31**, 1001-1005, doi:10.1111/j.1478-3231.2011.02521.x (2011).
146 Eldin, S. S. *et al.* Risk factors and immune response to hepatitis E viral infection among acute hepatitis patients in Assiut, Egypt. *The Egyptian journal of immunology / Egyptian Association of Immunologists* **17**, 73-86 (2010).
147 Talaat, M. *et al.* Sentinel surveillance for patients with acute hepatitis in Egypt, 2001-04. *Eastern Mediterranean health journal = La revue de sante de la Mediterranee orientale = al-Majallah al-sihhiyah li-sharq al-mutawassit* **16**, 134-140 (2010).
148 Youssef, A. *et al.* Molecular epidemiological study of hepatitis viruses in Ismailia, Egypt. *Intervirology* **52**, 123-131, doi:10.1159/000219385 (2009).
149 Abdel-Wahab, M., Mostafa, M., Sabry, M., el-Farrash, M. & Yousef, T. Aflatoxins as a risk factor for hepatocellular carcinoma in Egypt, Mansoura Gastroenterology Center study. *Hepato-gastroenterology* **55**, 1754-1759 (2008).
150 Abdel-Wahab, M. *et al.* Epidemiology of hepatocellular carcinoma in lower Egypt, Mansoura Gastroenterology Center. *Hepato-gastroenterology* **54**, 157-162 (2007).
151 Mikhail, N. N. *et al.* Prospective study of cross-infection from upper-GI endoscopy in a hepatitis C-prevalent population. *Gastrointestinal endoscopy* **65**, 584-588, doi:10.1016/j.gie.2006.07.033 (2007).
152 Zakaria, S. *et al.* Changing patterns of acute viral hepatitis at a major urban referral center in Egypt. *Clinical infectious diseases : an official publication of the Infectious Diseases Society of America* **44**, e30-36, doi:10.1086/511074 (2007).
153 El Gaafary, M. M. *et al.* Surveillance of acute hepatitis C in Cairo, Egypt. *Journal of medical virology* **76**, 520-525, doi:10.1002/jmv.20392 (2005).
154 el-Zayadi, A. R. *et al.* Hepatocellular carcinoma in Egypt: a single center study over a decade. *World journal of gastroenterology : WJG* **11**, 5193-5198 (2005).
155 Takagi, H. *et al.* Liver disease in Alexandria, Egypt. *Kitakanto Medical Journal* **53**, 175-177 (2003).
156 Zaki, A. *et al.* Morbidity of schistosomiasis mansoni in rural Alexandria, Egypt. *Journal of the Egyptian Society of Parasitology* **33**, 695-710 (2003).
157 Blanton, R. E. *et al.* Population-based differences in Schistosoma mansoni- and hepatitis C-induced disease. *The Journal of infectious diseases* **185**, 1644-1649, doi:10.1086/340574 (2002).
158 Rahman El-Zayadi, A. *et al.* Prevalence and epidemiological features of hepatocellular carcinoma in Egypt - A single center experience. *Hepatology Research* **19**, 170-179, doi:http://dx.doi.org/10.1016/S1386-6346%2800%2900105-4 (2001).
159 Abdel-Wahab, M. *et al.* Hepatocellular carcinoma in Mansoura-Egypt: experience of 385 patients at a single center. *Hepato-gastroenterology* **47**, 663-668 (2000).
160 Khalifa, A., Mady, E. A., Abadeer, N. & Kamal, A. Differential tumor markers and hepatitis markers profile in liver tumors. *Anticancer Research* **19**, 2495-2500 (1999).
161 Angelico, M. *et al.* Chronic liver disease in the Alexandria governorate, Egypt: contribution of schistosomiasis and hepatitis virus infections. *J Hepatol* **26**, 236-243 (1997).
162 Darwish, M. A., Amer, A. F., El-Moeity, A. A. & Darwish, N. M. Association of hepatitis C virus with liver cirrhosis and hepatocellular carcinoma compared with hepatitis B virus in Egyptian patients. *The Journal of the Egyptian Public Health Association* **72**, 569-589 (1997).
163 Madwar, M. A., Shaker, M. K., Atta, M. A., El Khashaab, T. H. & Mohamed, M. K. A prospective study: prediction of the first variceal haemorrhage in schistosomal and non schistosomal liver disease. *The Journal of the Egyptian Public Health Association* **72**, 395-409 (1997).
164 Quinti, I. *et al.* HCV infection in Egyptian patients with acute hepatitis. *Digestive diseases and sciences* **42**, 2017-2023 (1997).
165 Gomatos, P. J. *et al.* Sporadic acute hepatitis caused by hepatitis E virus in Egyptian adults. *Clinical infectious diseases : an official publication of the Infectious Diseases Society of America* **23**, 195-196 (1996).

166 Waked, I. A. *et al.* High prevalence of hepatitis C in Egyptian patients with chronic liver disease. *Gut* **37**, 105-107 (1995).
167 El-Gohary, A., Hassan, A., Uchida, T., Shikata, T. & Nooman, Z. Prevalence of hepatitis E virus among acute sporadic hepatitis patients in Suez. *International Hepatology Communications* **2**, 218-222, doi:http://dx.doi.org/10.1016/0928-4346%2894%2990074-4 (1994).
168 Darwish, M. A., Issa, S. A., Aziz, A. M., Darwish, N. M. & Soliman, A. H. Hepatitis C and B viruses, and their association with hepatocellular carcinoma in Egypt. *The Journal of the Egyptian Public Health Association* **68**, 1-9 (1993).
169 Mokhtar, G. M., Reda, S. M., Hafez, A. M., Faheem, M. S. & Kamel, A. M. Coxiella Burnetii in Egyptian Children with Hepatitis *Scientific Medical Journal* **8**, 1-12 (1996).
170 Salem, M., Ramadan, L. & El-Ansary, M. Prevalence of Anti-Hepatitis C and Hepatitis B among Patients with Viral Hepatitis in Egypt. *Scientific Medical Journal* **4**, 105-112 (1992).
171 Mostafa, B. & Ahmed, E. Prevalence of oral lichen planus among a sample of the Egyptian population. *Journal of clinical and experimental dentistry* **7**, e7-e12, doi:10.4317/jced.51875 (2015).
172 Gabr, M., Khodeir, S., Elsawy, A. A. & Elraouf, Y. A. Study of upper gastrointestinal bleeding in hepatocellular carcinoma in Nile Delta: Role of portal vein thrombosis. *Hepatology International* **1)**, S249, doi:http://dx.doi.org/10.1007/s12072-014-9519-7 (2014).
173 Mousa, S. M. Hepatitis C among Egyptian Patients Referred for Bone Marrow Examination: Seroprevalence and Analysis of Hematological Findings. *Bone marrow research* **2014**, 549716, doi:10.1155/2014/549716 (2014).
174 Taha, E. A. *et al.* Study of the impact of viral load of hepatitis C on patients with concomitant psoriasis vulgaris. *Arab journal of gastroenterology : the official publication of the Pan-Arab Association of Gastroenterology* **15**, 98-102, doi:10.1016/j.ajg.2014.08.001 (2014).
175 Abd-El-Moneim, S. E., Welaya, K. Y., El-Assal, S., Salama, O. E. & Ghanem, H. M. The effect of HCV serological status on doxorubicin-based chemotherapy-induced toxicity and disease-free survival in breast cancer patients. *Journal of Clinical Oncology* **1)** (2013).
176 Azim, H., Abdelmalek, R., Abdelrhman, O., Abdeltawab, R. & Shahin, M. Diffuse large b cell lymphoma (DLBCL) coexistent with hepatitis c infection; single institutional experience from Egypt. *Haematologica* **98**, 646 (2013).
177 El-Khalawany, M. *et al.* A multicenter clinicomycological study evaluating the spectrum of adult tinea capitis in Egypt. *Acta dermatovenerologica Alpina, Pannonica, et Adriatica* **22**, 77-82 (2013).
178 Mattar, M., Tawfik, N., Elhosseiny, N. & Morad, M. Demographic analysis of 90 polycythemia vera egyptian cases : An egyptian reference center experience. *Blood* **122 (21)** (2013).
179 Aboueisha, H. *et al.* A retrospective evaluation of causes of exempting living liver donors in an Egyptian centre. *Arab journal of gastroenterology : the official publication of the Pan-Arab Association of Gastroenterology* **14**, 10-13, doi:10.1016/j.ajg.2013.01.003 (2013).
180 El Garf, A. *et al.* Prevalence and clinical presentations of hepatitis C virus among patients admitted to the rheumatology ward. *Rheumatology international* **32**, 2691-2695, doi:10.1007/s00296-011-2014-8 (2012).
181 Youssef, S. S., Nasr, A. S., El Zanaty, T., El Rawi, R. S. & Mattar, M. M. Prevalence of occult hepatitis C virus in egyptian patients with chronic lymphoproliferative disorders. *Hepatitis research and treatment* **2012**, 429784, doi:10.1155/2012/429784 (2012).
182 El Husseiny, N., Mohamed, S. & Mattar, M. Myelodysplastic syndrome. Egyptian experience. *Haematologica* **96**, 516-517 (2011).
183 El-Sabah, A. A., El-Metwally, M. T. & Abozinadah, N. Y. Hepatitis C and B virus in schistosomiasis patients on oral or parenteral treatment. *Journal of the Egyptian Society of Parasitology* **41**, 307-314 (2011).
184 Mahmoud, G. A., Zayed, H. S., Sherif, M. M. & Mostafa, M. M. Characteristics of rheumatoid arthritis patients with concomitant hepatitis C virus infection. *Egyptian Rheumatologist* **33**, 139-145, doi:http://dx.doi.org/10.1016/j.ejr.2011.05.003 (2011).
185 Abou-Zeid, A. A. & El-Sayegh, H. K. Toll-like receptor 3 gene expression in Egyptian patients with glomerulonephritis and hepatitis C virus infection. *Scandinavian journal of clinical and laboratory investigation* **71**, 456-461, doi:10.3109/00365513.2011.585660 (2011).

186 Sabry, A. Proteinuria among renal transplant patients and its relation to hepatitis C virus and graft outcome: a single center experience. *Experimental and clinical transplantation : official journal of the Middle East Society for Organ Transplantation* **8**, 91-97 (2010).
187 Sharaf-Eldeen, S., Salama, K., Eldemerdash, S., Hassan, H. M. S. & Semesem, M. Hepatitis B and C Viruses in Egyptian children with malignancy. *Journal of Medical Sciences* **7**, 1003-1008 (2007).
188 Sabry, A. *et al.* HCV associated glomerulopathy in Egyptian patients: clinicopathological analysis. *Virology* **334**, 10-16, doi:10.1016/j.virol.2005.01.013 (2005).
189 Demian, A. D. Prevalence of anaesthetic co-morbid factors among urological patients in a tertiary referral centre in Egypt. *Egyptian Journal of Anaesthesia* **20**, 325-330 (2004).
190 Sabry, A. A. *et al.* A comprehensive study of the association between hepatitis C virus and glomerulopathy. *Nephrology, dialysis, transplantation : official publication of the European Dialysis and Transplant Association - European Renal Association* **17**, 239-245 (2002).
191 Meir, H., Balawi, I., Nayel, H., El Karaksy, H. & El Haddad, A. Hepatic dysfunction in children with acute lymphoblastic leukemia in remission: relation to hepatitis infection. *Medical and pediatric oncology* **36**, 469-473, doi:10.1002/mpo.1111 (2001).
192 Hassaballa, M., Saadi, G., Salama, Z. & Eid, F. Combined Assessment of Liver and Kidney Functions in HCV +ve Kidney Transplant Recipients. *Scientific Medical Journal* **8**, 39-49 (1996).
193 Mostafa, A. *et al.* Seroprevalence of Hepatitis B and C in Pediatric Malignanices. *Journal of the Egyptian Nat Cancer Inst* **15**, 33-42 (2003).
194 El-Mougy, F. A. *et al.* Aberrant p16INK4A methylation: Relation to viral related chronic liver disease and hepatocellular carcinoma. *South Asian journal of cancer* **3**, 1-4, doi:10.4103/2278-330x.126498 (2014).
195 Kalil, K. A., Farghally, H. S., Hassanein, K. M., Abd-Elsayed, A. A. & Hassanein, F. E. Hepatitis C virus infection among paediatric patients attending University of Assiut Hospital, Egypt. *Eastern Mediterranean health journal = La revue de sante de la Mediterranee orientale = al-Majallah al-sihhiyah li-sharq al-mutawassit* **16**, 356-361 (2010).
196 Hammad, A. M. & Zaghloul, M. H. Hepatitis G virus infection in Egyptian children with chronic renal failure (single centre study). *Annals of clinical microbiology and antimicrobials* **8**, 36, doi:10.1186/1476-0711-8-36 (2009).
197 Mohamed, M. K. *et al.* Intrafamilial transmission of hepatitis C in Egypt. *Hepatology* **42**, 683-687, doi:10.1002/hep.20811 (2005).
198 Saleh, D. A. *et al.* Incidence and risk factors for hepatitis C infection in a cohort of women in rural Egypt. *Transactions of the Royal Society of Tropical Medicine and Hygiene* **102**, 921-928, doi:10.1016/j.trstmh.2008.04.011 (2008).
199 El-Sherif, A. *et al.* High false-negative rate of anti-HCV among Egyptian patients on regular hemodialysis. *Hemodialysis international. International Symposium on Home Hemodialysis* **16**, 420-427, doi:10.1111/j.1542-4758.2011.00662.x (2012).
200 Soliman, A. R., Momtaz Abd Elaziz, M. & El Lawindi, M. I. Evaluation of an isolation program of hepatitis C virus infected hemodialysis patients in some hemodialysis centers in egypt. *ISRN nephrology* **2013**, 395467, doi:10.5402/2013/395467 (2013).
201 Abdel-Wahab, M. *et al.* Factors affecting recurrence and survival after living donor liver transplantation for hepatocellular carcinoma. *Hepato-gastroenterology* **60**, 1847-1853 (2013).
202 Saleh, D. A. *et al.* Incidence and risk factors for community-acquired hepatitis C infection from birth to 5 years of age in rural Egyptian children. *Transactions of the Royal Society of Tropical Medicine and Hygiene* **104**, 357-363, doi:10.1016/j.trstmh.2010.01.009 (2010).
203 Meky, F. A. *et al.* Active surveillance for acute viral hepatitis in rural villages in the Nile Delta. *Clinical infectious diseases : an official publication of the Infectious Diseases Society of America* **42**, 628-633, doi:10.1086/500133 (2006).
204 Youssef Hassan, M. & Abdel Mooti Samra, M. HCV sero-conversion in HCV negative recipients of allogenic HSCT. *Vox Sanguinis* **105**, 179, doi:http://dx.doi.org/10.1111/vox.12048 (2013).

205 Abo Elmagd, E. K., Abdel-Wahab, K. S., Alrasheedy, Z. E. & Khalifa, A. S. An Egyptian study of mother to child transmission of hepatitis C virus. *International Journal of Virology* **7**, 100-108, doi:http://dx.doi.org/10.3923/ijv.2011.100.108 (2011).

206 El Sayed Zaki, M., El Aal, A. A., Badawy, A., El-Deeb, D. R. & El-Kheir, N. Y. Clinicolaboratory study of mother-to-neonate transmission of hepatitis E virus in Egypt. *American journal of clinical pathology* **140**, 721-726, doi:10.1309/ajcpt55tdmjnpllv (2013).
